# Genetic information from discordant sibling pairs points to ESRP2 as a candidate trans-acting regulator of the CF modifier gene SCNN1B

**DOI:** 10.1038/s41598-020-79804-y

**Published:** 2020-12-31

**Authors:** Tim Becker, Andreas Pich, Stephanie Tamm, Silke Hedtfeld, Mohammed Ibrahim, Janine Altmüller, Nina Dalibor, Mohammad Reza Toliat, Sabina Janciauskiene, Burkhard Tümmler, Frauke Stanke

**Affiliations:** 1 Institute for Community Medicine, Ernst Moritz Arndt University, Greifswald, Germany; 2 xValue GmbH, Villich, Germany; 3 Research Core Unit Proteomics, Hannover Medical School, Hannover, Germany; 4 Department of Paediatric Pneumology, Allergology and Neonatology, Hannover Medical School, Hannover, Germany; 5 Cologne Center for Genomics, University of Cologne, Cologne, Germany; 6 German Center for Lung Research (DZL), Partner site BREATH, Hannover, Germany; 7 Department of Pneumology, Hannover Medical School, Hannover, Germany

**Keywords:** Gene regulation, Genetic association study

## Abstract

*SCNN1B* encodes the beta subunit of the epithelial sodium channel ENaC. Previously, we reported an association between SNP markers of SCNN1B gene and disease severity in cystic fibrosis-affected sibling pairs. We hypothesized that factors interacting with the SCNN1B genomic sequence are responsible for intrapair discordance. Concordant and discordant pairs differed at six *SCNN1B* markers (Praw = 0.0075, Pcorr = 0.0397 corrected for multiple testing). To identify the factors binding to these six SCNN1B SNPs, we performed an electrophoretic mobility shift assay and captured the DNA–protein complexes. Based on protein mass spectrometry data, the epithelial splicing regulatory protein ESRP2 was identified when using *SCNN1B*-derived probes and the ESRP2-*SCNN1B* interaction was independently confirmed by coimmunoprecipitation assays. We observed an alternative SCNN1B transcript and demonstrated in 16HBE14o− cells that levels of this transcript are decreased upon ESRP2 silencing by siRNA. Furthermore, we confirmed that mildly and severely affected siblings have different ESPR2 genetic backgrounds and that ESRP2 markers are linked to the response of CF patients’ nasal epithelium to amiloride, indicating ENaC involvement (Pbest = 0.0131, Pcorr = 0.068 for multiple testing). Our findings demonstrate that sibling pairs clinically discordant for CF can be used to identify meaningful DNA regulatory elements and interacting factors.

## Introduction

Genetic variation in humans contributes significantly to phenotypic variation. The question of which single nucleotide polymorphism (SNP) determines disease outcome and/or severity, has been addressed in more than 2000 genome-wide association studies (GWAS)^1^. However, the results have revealed that more than 90% of the polymorphisms identified by GWAS do not directly alter the gene’s coding sequence. This has led to the conclusion that clinically relevant variation of the human genome mediates gene regulation, i.e., the transcript expression level or the composition of transcript isoforms^2^. Recent genome and epigenome studies have substantiated this hypothesis^3^. Enrichment of elements known for covalent modifications of DNA bases or their associated nucleosomes^4^ among disease- and trait-associated genetic variants determined by GWAS has also been noted.

The gene affected in cystic fibrosis (CF), cystic fibrosis transmembrane conductance regulator (CFTR), encodes a chloride- and bicarbonate channel of epithelia^5–7^ that localizes with the epithelial sodium channel ENaC at the apical membrane of many^8^, albeit not all^9^, epithelial cells. Both CFTR and ENaC act synergistically to regulate salt and fluid transport across the epithelium^8,10^, and the SCNN1B gene, encoding the beta subunit of ENaC, is a highly plausible modifier gene of CF.

Previously, we focused on the three genes encoding the subunits of ENaC as candidate genes in the European CF twin and sibling study^11^. In an association study on extreme phenotypes, anthropometry and lung function data were used to select patients whose clinical data fell below the 25th centile (severely affected) or above the 75th centile (mildly affected) for both clinical parameters^12^. Three groups of affected patient pairs were defined as follows by a ranking algorithm used to describe the severity of CF: concordant mildly affected sibling pairs, concordant severely affected sibling pairs and discordant sibling pairs. Discordant sibling pairs were composed of one mildly and one severely affected sibling^12^.

When discordant sibling pairs were compared to concordant sibling pairs, one *SCNN1B* haplotype defined by SNPs rs238547–rs152730–rs250563 occurred more frequently among discordant than among concordant siblings^11^. We concluded that this signal cannot be fully explained by a variant observed within *SCNN1B* because discordant siblings have a dissimilar phenotype by definition^12^, and yet these siblings share an *SCNN1B* intragenic haplotype^11^. Our working hypothesis relied on the idea that the association signal in *SCNN1B* delineates a functional regulatory element, whereby a DNA-binding protein encoded *in trans* interacts with this regulatory element, stably binding to the haplotype of the regulatory element that is predominant among discordant siblings. Hence, the genetic variation of the interaction partner can determine the phenotype causing intrapair discordance in affected sibling pairs.

In association studies involving affected patient pairs, interaction between a regulatory element and a DNA-binding protein may result in a paradoxical situation. The regulatory element is recognized through an INTERpair comparison by an association with the phenotype “discordance of sibs”. However, genetic information at the regulatory element is shared by both siblings within a pair, which provides an opportunity to identify the DNA-binding protein encoded *in trans* by an INTRApair comparison. If the phenotype is caused by interaction of the DNA-binding protein with the regulatory element, mildly and severely affected siblings of discordant pairs must carry different genetic information at the locus encoding the DNA-binding protein.

## Results

### Six SNPs within SCNN1B differ between concordant and discordant CF patient pairs

**Figure 1:**
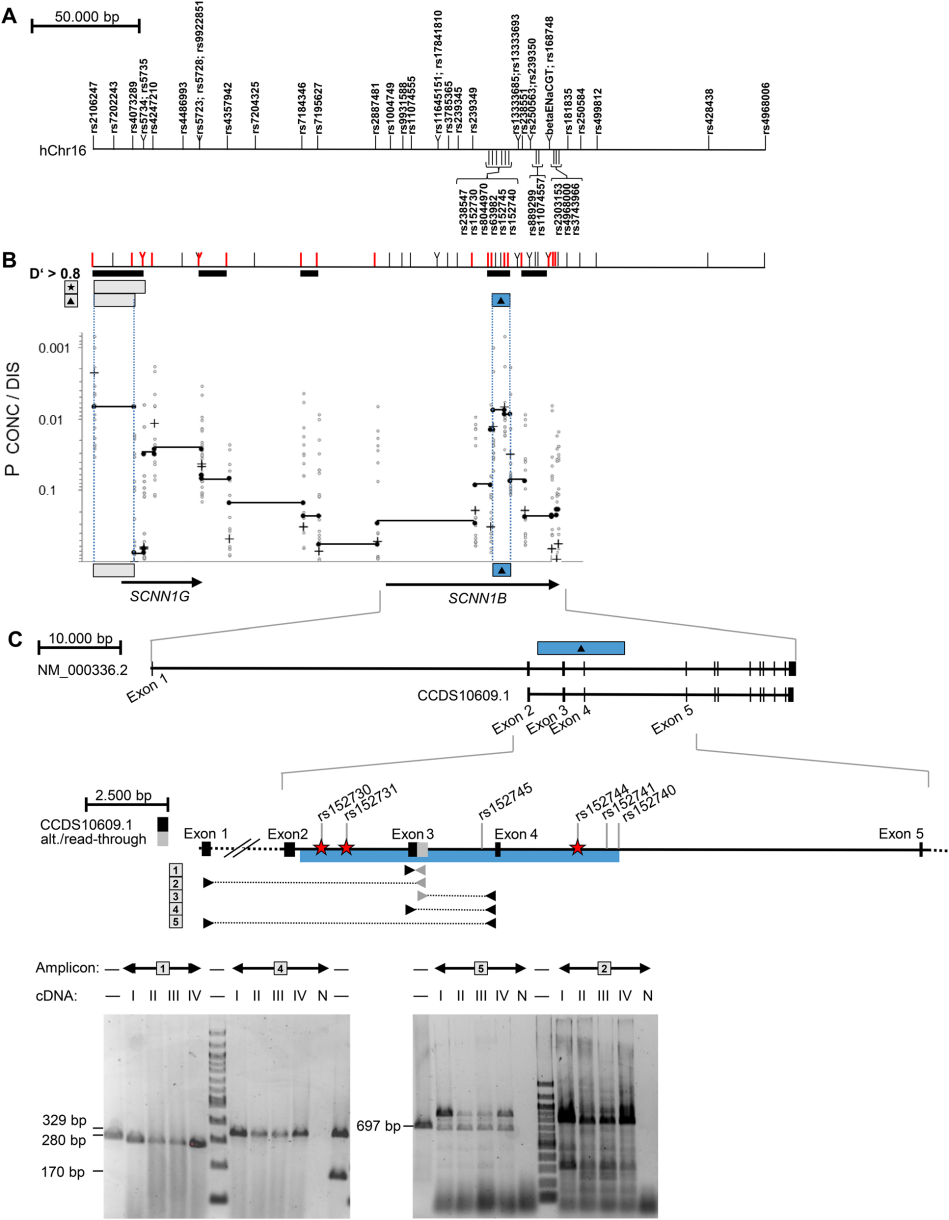
Mapping of the association signal determining intrapair discordance among F508del-CFTR homozygous CF sibling pairs, identification of an alternative SCNN1B transcript and position of SNPs analyzed by EMSA-PSeq. (**A**) Position of SNP and microsatellite markers on the genomic sequence of the *SCNN1G*/*SCNN1B* region. The position of genetic markers was derived from the genomic sequence on NC_000016 (assembly 03-Feb-2014; GRCh38; region 23302270–23381299). Nomenclature of *SCNN1B* exons was derived from Voilley et al. 1995^59^ and Saxena et al. 1998^60^, who have described exon/intron borders and the coding sequence for a 640 amino acid SCNN1B protein encoded by 13 exons (NP_000327.2; GI: 124301196; CCDS10609.1). The position of intragenic primers and expected sizes of amplicons covering exon 1–5 of *SCNN1B* are based on this coding sequence. SNPs rs5735, rs5723, rs1004749, rs238547, rs152730, rs250536 and on microsatellite betaENaCGT were previously typed^11^ and further 49 SNPs were genotyped for fine-mapping (this work). (**B**) Haplotype blocks, informative markers and association signals in the SCNN1B/SCNN1G region. Markers depicted in red are informative (minimal allele frequency > 0.4) and were used to describe haplotype blocks (a measure of linkage disequilibrium D′ > 0.8) and map association signals as previously described^13^. Briefly, to map association signals, we defined genomic segments by adjacent ancestral informative markers that isolate the fragment that carries the causative variant(s)^13^. *p* values were generated by the software package FAMHAP^55–57^. *p* values refer to the best signal observed for a two-marker-combination of the 20-marker-set (Pbest) and the corrected value for multiple testing of 20 markers (Pcorr). Symbols refer to: Star: signal obtained by transmission disequilibrium test in the set of 37 sibling pairs families with extreme phenotypes (Pbest = 0.00101 for rs2106247–rs4073289; Pcorr = 0.0561 corrected for multiple testing of 20 informative markers); Filled triangle: association with intrapair discordance (Pbest = 0.0007 for rs2106247–rs152745; Pcorr = 0.0397 corrected for multiple testing of 20 informative markers) comparing 14 discordant and 23 concordant patient pairs. Uncorrected raw *p* values are shown for single markers (+), 2-marker-haplotypes of adjacent informative markers (filled circles; genomic segments spanned by two adjacent markers are linked by a black line) and 2-marker-haplotypes of non-adjacent markers (open circles). The SCNN1B association signal observed for intrapair discordance (filled triangle) was seen on the neighboring segments rs152730–rs152745 (Praw = 0.0075) and rs152745–rs152740 (Praw = 0.00869). A second independent association signal with intrapair discordance in the SCNN1G locus colocalizes with the previously noted survivor effect at SCNN1G^11^ and was excluded from further analysis because of this confounding factor. (**C**) SNPs targeted by EMSA-PSeq and combinatorial PCR describing an alternative transcript generated by exon-read-through. Interaction partners of SNPs rs152730, rs152731 and rs152744, indicated by red star symbols, have been characterized by EMSA-PSeq. Equivalent amounts of cDNA from T84 (I), 16HBE14o-(II), CFTE29o-(III) and CFBE41o-(IV) or no template (negative control, N) were analyzed by combinatorial PCR with 5 amplicons. The grey intronic sequence (amplicon 1, 2, 3) was derived from ESTs BM694355 and BU730506 (see text and supplement for details). Intron-spanning primers targeting the SCNN1B reference mRNA (amplicons 4 and 5) as well as amplicon 1 targeting the alternative transcript and the 5′ UTR were observed in all cell lines. The size of amplified products from all amplicons was in accordance with the expected length. Size markers are loaded in lanes indicated by “—” and contain either a 100-bp-ladder or PCR products of 697 bp, 329 bp, 280 bp and 170 bp as indicated adjacent to the gel. Sanger sequencing of amplicon 1 confirmed the exon3-read-through and Sanger sequencing of amplicon 2 products verified its identity derived from a spliced mRNA as it showed a joined exon1 and exon2 sequence.

We previously reported that intrapair discordance for CF disease severity is associated with three intragenic markers spanning *SCNN1B* from codon 3 to codon 293. To describe the genomic fragment for which concordant and discordant pairs carry different genetic information, we analyzed 7 previously typed markers^11^ and 49 SNPs genotyped for fine-mapping in the 16p12 region, encompassing the entire *SCNN1G/SCNN1B*-locus (Fig. 1). Next, we employed a haplotype-based fine-mapping strategy previously used to identify causative variants within this cohort^13^. To determine whether concordant and discordant pairs carried the same or different genetic information, we employed the software package FAMHAP to construct two-marker haplotypes composed of two informative markers. By using this approach, we found a significant difference in two-marker-haplotype distributions for two adjacent genomic fragments defined by markers rs152730–rs152745 and rs152745–rs152740 (Praw = 0.0075 and Praw = 0.00869, respectively; corrected for multiple testing of all informative markers at the *SCNN1G/SCNN1B*-locus Pcorr = 0.0397, Fig. 1). We concluded that the variant(s) that determine intrapair discordance are located on the genomic fragment between rs152730 and rs152740. Based on the allele frequency distribution among concordant and discordant pairs, we selected representatives for the contrasting haplotypes for Sanger resequencing of the mapped genomic fragment (Table 1). We chose three homozygotes for the haplotype TTAGA, two homozygotes for the haplotype GGAAT and one homozygote for the haplotype GTCAT for sequencing of the rs152730–rs152740 genomic fragment on contrasting alleles at markers rs152730–rs8044970–rs63982–rs152745–rs152740. We used long-range PCR to amplify an 8269 bp and an 8856 bp product encompassing the sequence of interest (Table 2). Sanger sequencing was performed using internal primers positioned every 500 bp on the forward and reverse strands. Using the software CodonCode Aligner, 476 primary reads with a median length of 737 bp were aligned to the reference sequence, assuring coverage of at least 4 reads per haplotype at each genomic position. Based on this alignment, we identified 6 SNP positions for carriers of the contrasting haplotypes for which concordant and discordant pairs had different genetic information. At the six SNPs rs152730–rs152731–rs152745–rs152744–rs152741–rs152740, alleles associated with intrapair concordance carried the haplotype GCAGTT; in contrast, alleles associated with intrapair discordance carried the haplotype TTGACA. None of these six SNPs reside within the coding sequence of *SCNN1B.* However, according to in silico analyses, they possibly alter the secondary structure of the pre-mRNA (SupplTab. 1, SupplTab. 2, SupplFig. 1).

**Table 1:** Haplotype and diplotype distribution observed among concordant and discordant cystic fibrosis F508del homozygous sibling pairs for markers rs152730, rs8044970, rs63982, rs152745, rs152740.

	Freq. among 14 discordant pairs	Freq. among 23 concordant pairs	
rs152730–rs8044970–rs63982–rs152745–rs152740 haplotype			
TTAGA	0.839	0.599	Pcorr = 0.0397
GTCAT	0.051	0.185	
GGAAT	0.036	0.129	
Other pooled^a^	0.074	0.087	

rs152730–rs8044970–rs63982–rs152745–rs152740 haplotype			
TTAGA/TTAGA^b^	0.714	0.400	Pcorr = 0.049920
TTAGA/GTCAT	0.071	0.201	
TTAGA/GGAAT	0.071	0.127	
GGAAT/GGAAT^b^	0.000	0.020	
GTCAT/GTCAT^b^	0.000	0.059	
Other pooled	0.144	0.193	

^a^Four rare haplotypes (Freq. < 0.05) were observed among discordant pairs, 8 rare haplotypes (Freq. < 0.05) were observed among concordant pairs.

^b^To identify all genetic variants associated with intrapair discordance on the genomic fragment rs12730–rs152740, the entire 8000 bp genomic fragment was compared by Sanger re-sequencing for three homozygotes for TTAGA, two homozygotes for GGAAT and one homozygote for GTCAT (see Table 2).

**Table 2:** Variants observed on contrasting haplotypes identified after Sanger re-sequencing of the 8000 bp genomic fragment defined by rs152730–rs152740 associated with intrapair discordance.

Haplotype at rs152730–rs8044970–rs63982–rs152745–rs152740	Associated with intrapair disease manifestation	rs152730^a^	rs62029384	rs8044970^a^	rs8044984	rs80443907	rs152731	rs152732	rs180878	rs152733
TTAGA	Discordant	T	C	T	T	C	T	C	T	T
GGAAT	Concordant	G	T	G	G	T	C	C	T	T
GTCAT	Concordant	G	C	T	T	C	C	T	G	C
Different between TTAGA and GGAAT as well as GTCAT?		Yes	No	No	No	No	Yes	No	No	No

Haplotype at rs152730–rs8044970–rs63982–rs152745–rs152740	Associated with intrapair disease manifestation	rs63982^a^	rs152745^a^	rs8062922	rs152744	rs62029385	rs152743	rs152741	rs57406669	rs152740^a^
TTAGA	Discordant	A	G	C	A	C	G	C	C	A
GGAAT	Concordant	A	A	T	G	T	G	T	G	T
GTCAT	Concordant	C	A	C	G	C	A	T	C	T
Different between TTAGA and GGAAT as well as GTCAT?		No	Yes	No	Yes	No	No	Yes	No	Yes

^a^rs152730–rs8044970–rs63982–rs152745–rs152740 were used to map the fragment associated with intrapair discordance and define the contrasting haplotypes TTAGA (associated with intrapair discordance) and GGAAT as well as GTCAT (both associated with intrapair concordance); see Table 1.

### An uncommon alternative *SCNN1B* transcript generated by intron retention in epithelial cell lines

Because none of the six identified SNPs affect the amino acid sequence of the SCNN1B protein, we next aimed to determine whether *SCNN1B* undergoes alternative splicing. Alternative transcripts were inferred from mapped expressed sequence tags (ESTs, SupplFig. 2). As a source for polyA + RNA, we used T84 cells, which are derived from colon carcinoma, 16HBE14o-cells, which are virus-transformed non-CF respiratory epithelial cells, and CFBE41o- and CFTE29o-cells, both of which are immortalized respiratory epithelial cells derived from F508del-CFTR homozygous CF patients. Primers for combinatorial reverse-transcription PCR were designed to reflect ESTs reported for SCNN1B in the area of interest defined by SNPs rs152730–rs152740 (SupplFig. 2A). By using primers located in exons 3 and 4 or exons 3 and 5, we detected wild-type SCNN1B in all four epithelial cell lines (Fig. 1C). Additionally, we amplified a 280 bp product using one primer located within exon 3 and one primer located 100 bp 3′ of the splice site at the end of exon 3 (Fig. 1C and SupplFig. 2). We specifically investigated this intronic sequence because it has been reported to be retained in EST clones BM694355 and BU730506, which are generated from a cDNA library prepared from retinal pigment epithelium of a healthy adult male. Primers designed to detect ESTs AW844136, CV337204 and BX485038 did not amplify a product (SupplFig. 2). The 280 bp product, derived from the alternative SCNN1B transcript generated by exon read-through, was reliably amplified from T84 derived cDNA even when the RNA was pretreated with DNAse (SupplFig 2). Moreover, Sanger sequencing of the alternative product confirmed its identity at the exon 3/intron 3 border. Hence, the alternative mRNA was generated by an exon read-through event and matched the genomic sequence by the base (SupplFig 2E). Furthermore, primers placed upstream in exon 1 encoding the 5′ UTR of SCNN1B in combination with a primer placed on the retained intron sequence yielded a product from cDNA (Fig. 1C). Conversely, no signal was observed when using a primer located in the downstream exon 5 in combination with the retained intron sequence (data not shown). To summarize, cancer-derived intestinal epithelial cells as well as virus-immortalized respiratory epithelial cells expressed an alternative *SCNN1B* transcript in which intron 3 was partially retained. If translated, this alternative transcript would preserve the reading frame at the end of exon 3 and would be translated into a protein that terminates prematurely after an 26 additional amino acids derived from the retained intron sequence, producing a severely truncated SCNN1B protein of 221 amino acids.

### The *SCNN1B* haplotype found in discordant pairs is enriched for predicted transcription factor binding sites

**Figure 2:**
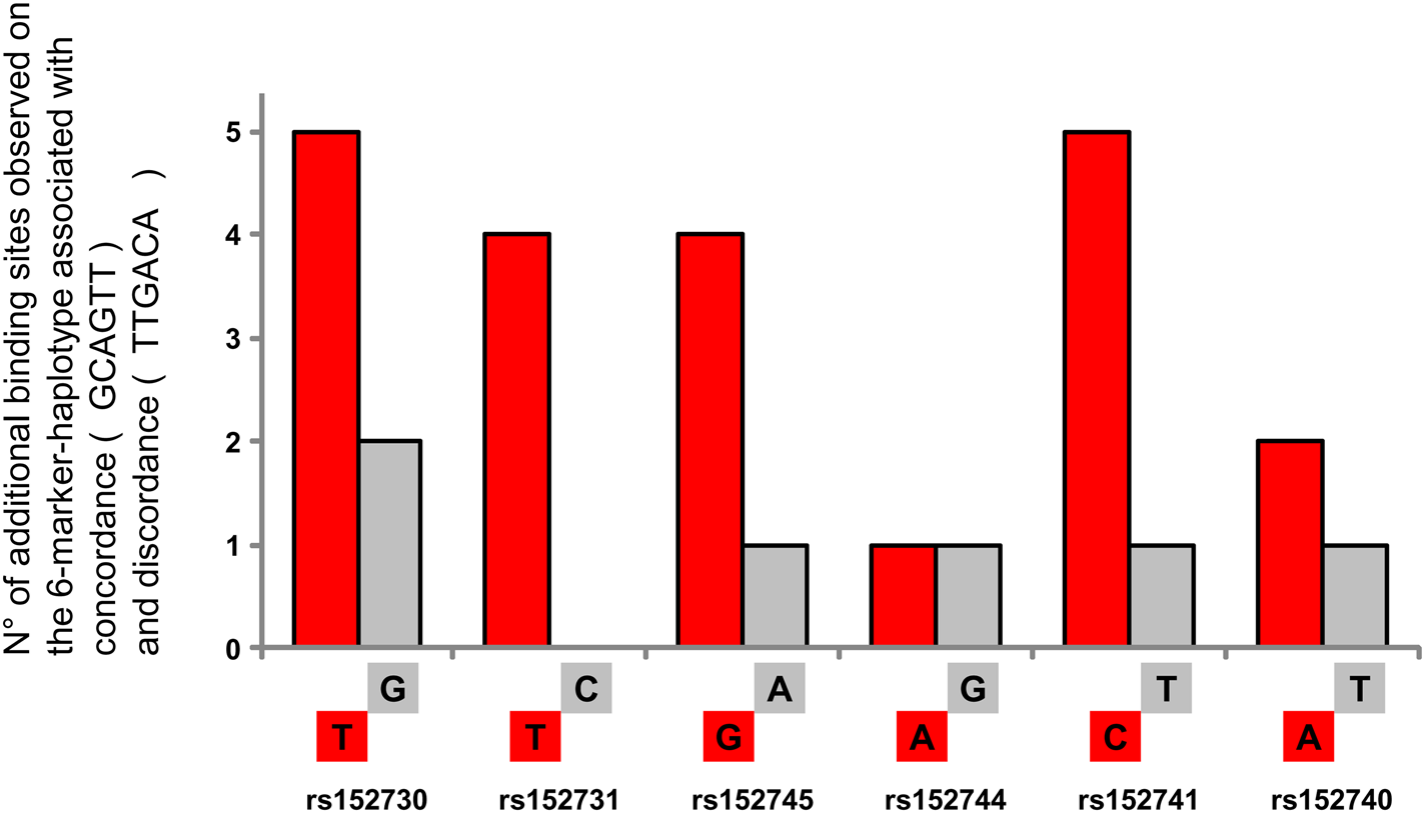
No. of predicted interaction partners unique to the six-marker-haplotype observed among concordant and discordant CF sibling pairs. Using the software Match at http://www.gene-regulation.com/^14^, binding sites for transcription factors were predicted for the two contrasting haplotypes composed of the six SNPs rs152730–rs152731–rs152745–rs152744–rs152741–rs152740. The figure shows the number of additional, allele-specific transcription factor binding sites predicted on the haplotype associated with intrapair concordance (grey) and for intrapair discordance (red). Please note that six allele-specific binding sites were predicted for haplotype GCAGTT while for TTGACA, associated with discordance, a total of 21 additional binding sites for transcription factors are predicted. Binding sites predicted to be shared for both alleles are not shown.

We next assumed that an allelic association with a discordant manifestation of CF severity is mediated by factors that recognize the allele enriched among discordant pairs. To test our hypothesis, we assessed whether the 6-marker-haplotype that is associated with intrapair discordance for CF severity, i.e., whether TTGACA at the six SNPs rs152730–rs152731–rs152745–rs152744–rs152741–rs152740 attracts different DNA-binding proteins compared to the GCAGTT allele that is observed among concordant sibling pairs. To identify potential transcription factor binding sites, we used the tool “Match” (available at http://www.gene-regulation.com/)^14^, which is based on a library of mononucleotide weight matrices from TRANSFAC6.0. The settings were restricted to vertebrate transcription factors and limited to minimize false negatives (estimated error rate of 10% for training data set). As an input sequence, we used both alleles at each of the six divergent SNPs and + /− 20 bp flanking sequences. Next, we compared the list of putative transcription factor binding sites between the input sequences derived from haplotypes associated with concordance and discordance and noted those predicted to interact with only one of the two contrasting alleles at each SNP. Surprisingly, only 6 binding sites were predicted for the haplotype observed among concordant sibling pairs; 21 predicted interactions were exclusively related to the six-marker-haplotype associated with intrapair discordance (Fig. 2, SupplTab 3a). Different from concordant sibling pairs, the haplotype observed among discordant sibling pairs had significantly more opportunities to interact with DNA-binding proteins (*p* = 0.048; in comparison to the expectancy value derived from 26 binding sites distributed equally between both haplotypes).

Next, we evaluated whether predicted interacting proteins of the *SCNN1B* haplotype TTGACA (SupplTab 3a) are associated with the response to amiloride upon superfusion of the nasal epithelium. The function of ENaC in vivo can be assessed based on the potential difference between the nasal epithelium and the subcutaneous space^15^. According to the nasal potential difference with the use of amiloride (indicative of ENaC-mediated sodium transport), except for GATA2Sat (Praw = 0.0456), none of the genes encoding predicted interaction partners showed an association with ENaC function (SupplTab 3b).

### Interaction partners of double-stranded DNA sequences can be captured with an electrophoretic mobility shift assay following protein sequencing (EMSA-PSeq)

**Figure 3:**
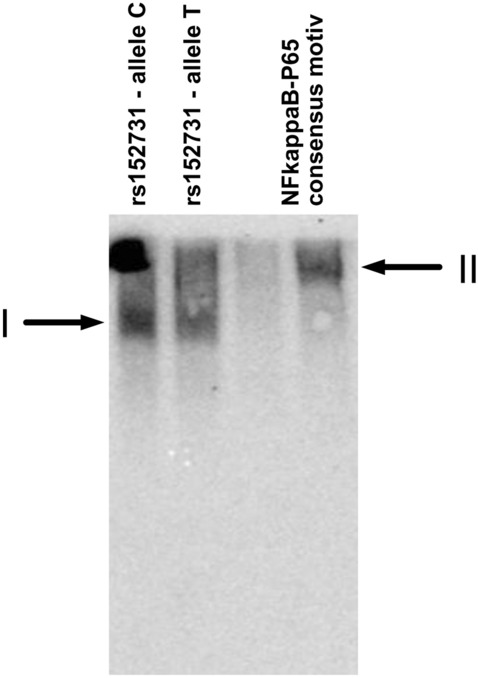
Electrophoretic mobility shift assay and subsequent protein sequencing (EMSA-PSeq). The dsDNA probes for SCNN1B SNP rs152731 allele C, SCNN1B SNP rs152731 allele T and the NFkappaB-P65 consensus motif^16^ were incubated with T84 nuclear extract. After non-denaturing 7% polyacrylamide electrophoresis, the samples were transferred by electrotransfer in a denaturing, SDS-containing buffer onto a membrane assembly whereby the gel was covered with an uncharged nylon membrane (Hybond C, Amersham), followed by two charged nylon membranes (Hybond N+, Amersham). Unbound oligonucleotides were transferred through the uncharged membranes and could be visualized on the charged nylon membranes (SupplFig4, SupplFig5). Probes derived from rs152731 gave rise to a high molecular weight complex I which differs from complex II generated with an NFkappaB-P65 consensus motif. P65, STAT3 and STAT6 were identified in the high molecular weight complex II, whereby for this experiment, the position corresponding to the visualized signal was excised from the second gel half loaded identically without electroblotting and subjected to protein mass spectrometry.

As our in silico analysis did not extend to DNA-binding proteins with unknown binding motifs, we aimed to identify interacting proteins by performing a modified electrophoretic mobility shift assay followed by protein sequencing of the DNA–protein complex (EMSA-PSeq; see supplement for experimental details). Briefly, we used nuclear extracts derived from epithelial cells and biotinylated 35-mer dsDNA probes that centrally carry one of the contrasting alleles of the SNPs as bait. To separate the unbound probe from the probe-protein-complexes, we performed native polyacrylamide gel electrophoresis, and the probe-protein-complexes were visualized after transfer of the separated samples to a membrane. The gel fragment corresponding to the signal generated by the probe-protein-complex was excised, and proteins within the excised gel fragment were identified by protein mass spectrometry. We used the NFkappaB-P65-consensus sequence^16^ for optimization of the experimental setup. Protein mass spectrometry and MASCOT analysis identified several hundred proteins per high-molecular weight complex. To enable the recognition of proteins that incidentally comigrate together with the probe/protein complex and/or that bind to any DNA unspecifically, and/or are introduced to the sample as contaminants during handling of the gel fragment, a set of 25 EMSA-PSeq experiments were performed in parallel for noise filtering (SupplFig 3). In the EMSA-PSeq sample obtained with the NFkappaB-P65-consensus probe (Fig. 3), we detected P65 (score 78, tagged by three specific peptides) as a unique signal within a total of 25 evaluated EMSA-PSeq data sets. Additionally, the EMSA-PSeq sample baited with the NFkappaB-P65-consensus probe uniquely attracted STAT3 and STAT6, both of which were not observed in any other of the 25 EMSA-PSeq datasets.

### Nucleic acid binding proteins attracted by probes of SNPs rs152730, rs152731 and rs152744 and identified as unique by EMSA-PSeq

To detect interaction partners of SCNN1B SNPs associated with intrapair discordance, we used certain experimental conditions to detect NFkappaB-P65 using a probe with a p65 consensus sequence as bait (Fig. 3). Although we varied the conditions for electrophoresis, electrotransfer and detection (see supplement for details), we were not able to obtain a reproducible high-molecular-weight complex for either allele of rs152745, rs152741 and rs152740. However, high-molecular-weight DNA–protein-complexes were observed with probes representing the two contrasting alleles at SNPs rs152730, rs152731 and rs152744. As described in detail within the Supplemental material, we have filtered the raw data set of 25 EMSA-PSeq experiments for low-expressed proteins annotated to have nucleic acid binding capabilities and attracted to only one SNP (SupplFig 3).

**Figure 4:**
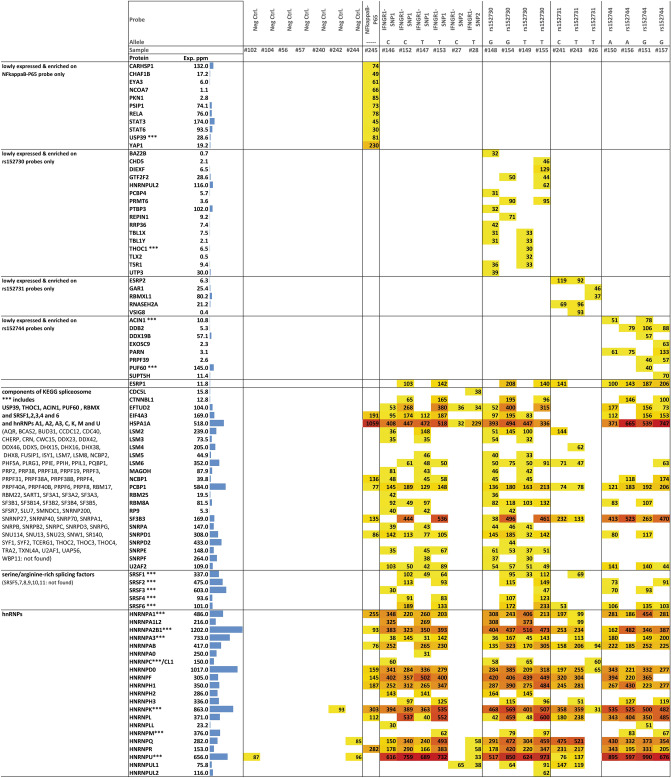
Proteins specific for rs152730, rs152731 and 152744 and abundantly present components of the spliceosome identified by EMSA-PSeq. Numbers correspond to MASCOT scores obtained for protein sequencing (primary data provided in: raw data xls supplement R3) which is a probability score that describes – 10LOG_10_(P), with P being the probability that the detected protein represents a significant match in this data set. ESRP2 was captured on rs152731 probes with a MASCOT score of 119 (C-allele, *p* = 10^–12^) and 92 (T-allele, *p* = 10^–9^), respectively. Data is shown for proteins specific for one SNP (top 40 rows), proteins that exhibit partial specificity (spliceosomal proteins) and proteins that were unspecifically found in the majority of probe-derived samples but were absent from the majority of negative controls (hnRNPs). Proteins specific for one probe or one SNP which were identified from the dataset derived for 25 EMSA-PSeq samples based on 1. Their absence from empty negative control samples 2. Their absence from EMSA-PSeq samples obtained with probes for other analyzed SNPs 3. Their expression levels of less than 200 ppm according to pax-db and 4. Their annotated capabilities to interact with nucleic acids (see SupplFig 3 for details). Data on ESRP1 is provided for comparison as both, ESRP1 and ESRP2 were interrogated in an association study (see Fig. 5). As many RNA-binding proteins were found among the proteins identified as SNP-specific, and as an alternative SCNN1B transcript has been observed, EMSA-PSeq data was systematically screened for constituents of the spliceosome. Exp. ppm denotes expression values retrieved from the Protein Abundances Across Organisms-database pax-db^61^ for the data set “colon integrated”, except for EYA3, BAZ2B, DIEXF, PRMT6, TLX2, UTP3, LSM3, LSM5, RP9 [Whole organism, (PeptideAtlas, Aug 2014)] and TLX2 [Whole organism (Integrated)]. Samples #26, #27 and #28 have been derived from denaturing SDS-polyacrylamide gels, hence these lack the multiprotein complexes observed in all other samples derived from native polyacrylamide electrophoresis. Details on experimental conditions are described in SupplTab 9.

Even when considering the inaccuracy of complex sizes after separation on the native polyacrylamide gel, high-molecular-weight complexes were incompatible with the interaction of a single protein found by EMSA-PSeq (SupplTab 4). Thus, several independent comigrating protein–protein and protein–protein-nucleic acid complexes were likely subjected to protein sequencing. Among the proteins identified as specific for the P65 consensus probe, P65 was one of 11 proteins (Fig. 4, SupplTab 4). Sixteen, five and eight proteins were identified uniquely for SNPs rs152730, rs152731 and rs152744, respectively, by EMSA-PSeq and data mining (Fig. 4, SupplTab 4). Since P65 was found with the same experimental and data evaluation strategy used for the data sets for the SCNN1B SNPs, we assume that our true-positive protein of interest has been captured as well.

### The ESRP2 genetic background is associated with the manifestation of the amiloride-sensitive sodium current in the nasal epithelium

To prove or disprove that a protein identified by EMSA-PSeq can influence ENaC function, we performed a candidate-gene-based association study among CF patients in which one of the studied phenotypes addresses ENaC function in the nasal epithelium in vivo^17^*.* To select a plausible genetic locus based on the list of 29 candidate proteins (Fig. 4, SupplTab 4), we excluded components of the spliceosome multiprotein complex. Among the remaining EMSA-PSeq-derived proteins, the epithelial-specific splicing regulatory protein 2 ESRP2^18,19^ was the most reasonable candidate. First, CF is an epithelial disease and ESRP2 is consistent with this feature^20,21^. Second, an alternative SCNN1B transcript was observed (Fig. 1C, SupplFig 2) and ESRP2 is plausible based on its role in transcript processing.

**Figure 5:**
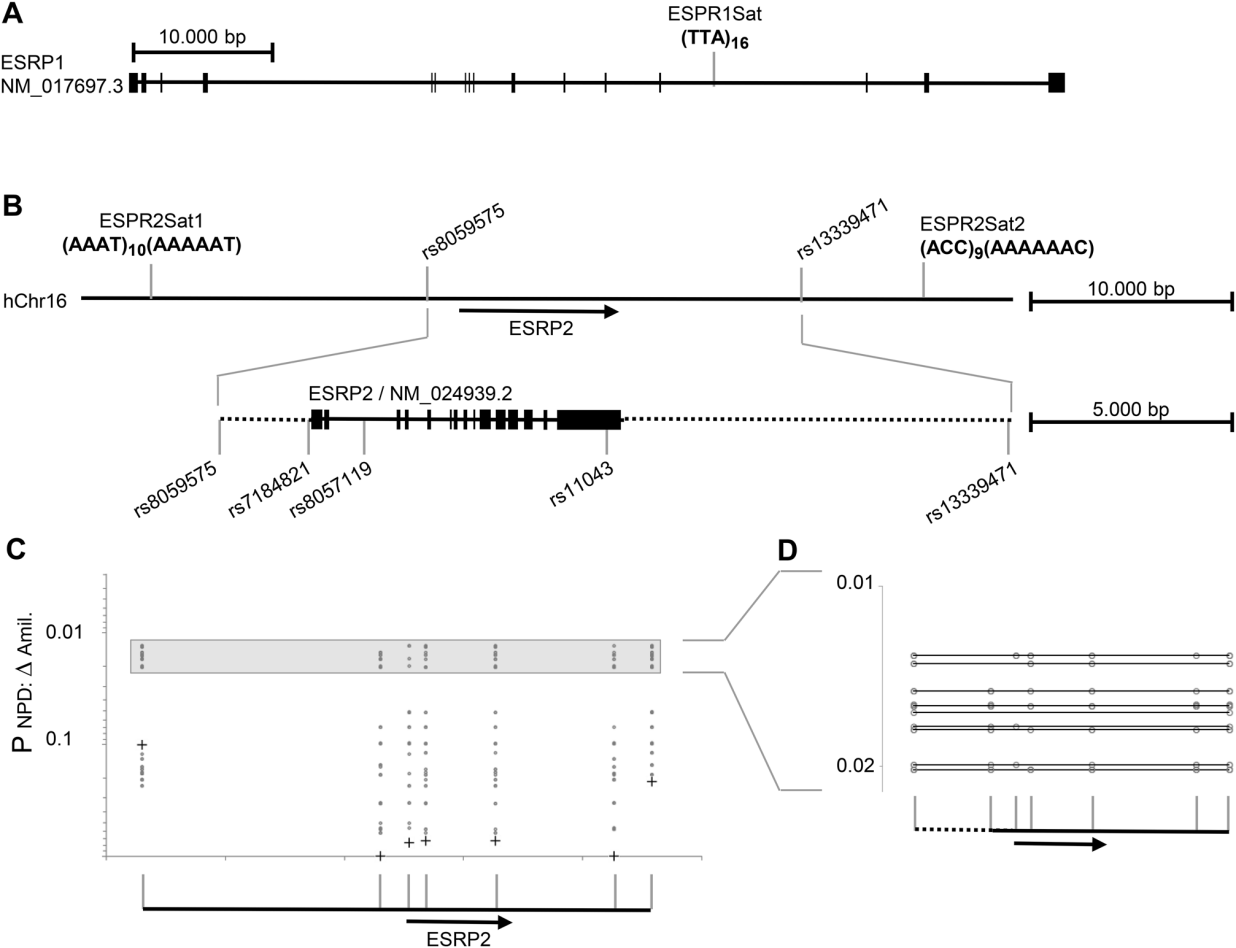
Association study on ESRP1 and ESRP2. (**A**) Position of intragenic microsatellite marker ESRP1Sat within ESRP1. ESRP1 was selected as control as this protein was identified at most probes used for EMSA-PSeq. In other words, no enrichment of ESRP1 with any probe used for EMSA-PSeq has been observed. No association of the ESRP1Sat allele distribution with CF disease severity or manifestation of the basic defect was seen (Praw > 0.2; data not shown). (**B**) Five SNPs and two intergenic microsatellite markers were typed on ESRP2. Markers on the ESRP2 locus were in strong linkage disequilibrium recognized by D′ values of 0.884, 0.918, 0.884 and 0.977 on the four segments between two adjacent SNP markers spanning ESRP2. (**C**,**D**) Analyzing markers in ESRP2, two association signals were observed: intrapair comparison of mildly versus severely affected sibling of discordant pairs was skewed (Praw = 0.1092 for ESRPSat2; Praw = 0.104 for the combination of ESRP2Sat1 and rs8057119; Pcorr = 0.323 corrected for multiple testing of 7 markers, data not shown) and an allelic association with the response to superfusion of the nasal epithelium with amiloride assessed by nasal potential difference measurement was seen (Pbest; raw = 0.0131; Pcorr = 0.068 corrected for multiple testing of 7 markers, data shown in (**C**,**D**). For this case-reference association study, F508del homozygotes with contrasting responses upon superfusion of the nasal epithelium with amiloride were compared in their ESRP2 genetic background. Cases were 16 patients with an amiloride response of 31 mV, references were 15 patients with 21 mV or less^17^. Raw *p* values of single markers are shown as “+” in (**C**), for raw *p* values obtained for multimarker haplotypes, marker positions are shown as “◯” in (**C**,**D**). For 0.02 > Praw > 0.01, multimarker combinations are displayed in (**D**).

We found ESRP2 exclusively on probes representing rs152731 whereas ESRP1 was detected on probes derived from the three EMSA-PSeq SNPs (Fig. 5). In the association study, the *ESRP1* marker ESRP1-Sat1 showed no association with the phenotype or severity of CF (Praw > 0.2). In contrast, ESRP2-Sat1 exhibited an association signal (Pbest = 0.04) that was confirmed with a second microsatellite and 5 SNPs (Pbest = 0.0131, Pcorr = 0.068 for multiple testing of 7 markers; Fig. 5) for the manifestation of amiloride-sensitive sodium conductance, a hallmark of ENaC function.

### The two rs152731 alleles are nonequivalent concerning ESRP2 binding

**Figure 6:**
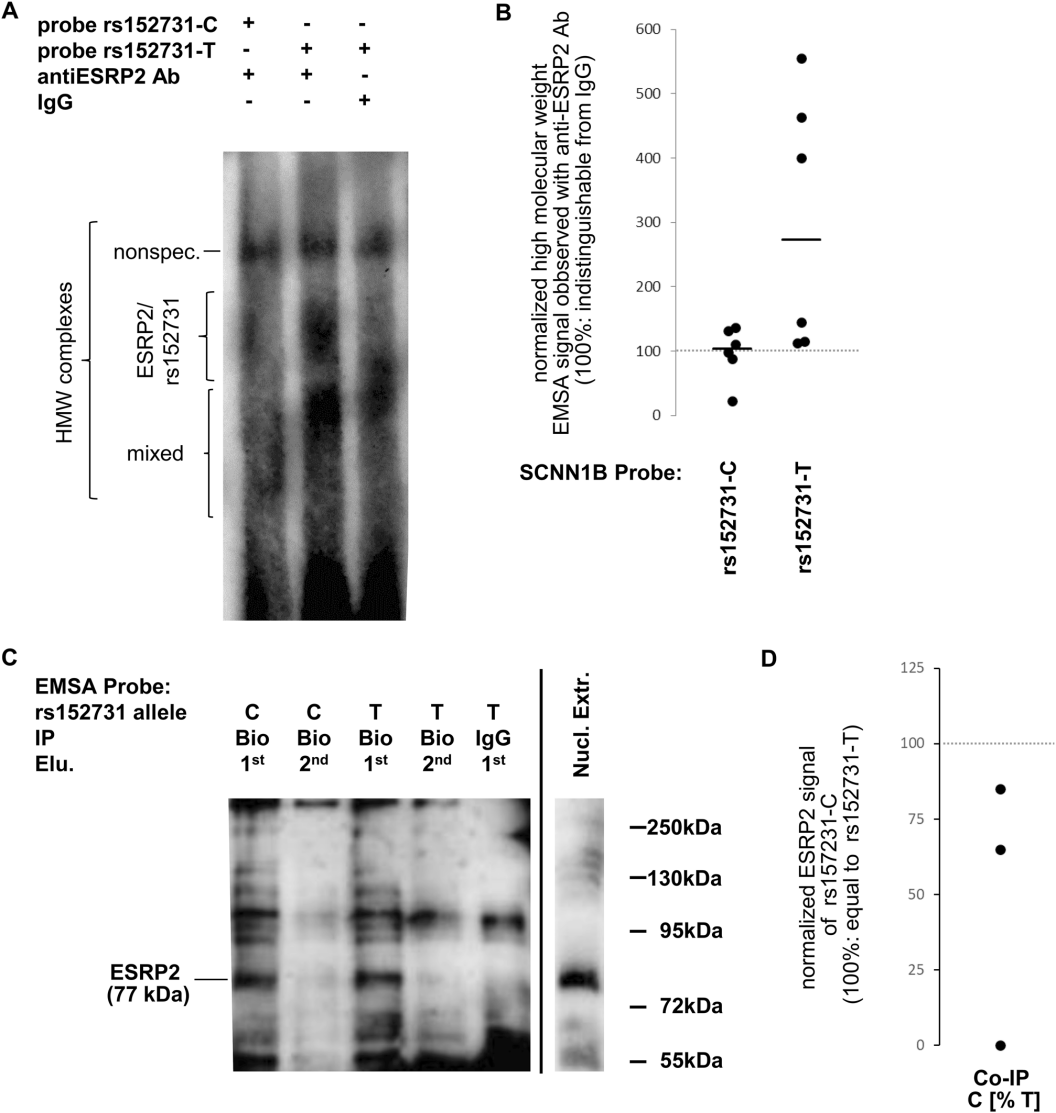
Binding of ESRP2 to rs152731-C and rs152731-T in EMSA and co-immunoprecipitation. (**A**) EMSA experiment using nuclear extract proteins derived from 16HBE14o. Signals marked “HMW” (high molecular weight) encompass different entities that can be differentiated by comparing the lane with anti-ESRP2-Ab to the control lane using IgG. Presence of ESRP2 in these HMW multi-protein complexes is deduced from the increased signal in the presence of an anti-ESRP2-antibody in comparison to the neighboring IgG control lane. Nonspec.: nonspecific signal, observed similarly with both, IgG control and anti-ESRP2-Ab. ESRP2/rs152731: signal observed predominantly in the presence of anti-ESRP2-Ab. Mixed: protein–DNA-complexes partially containing ESRP2 and partially nonspecific. The number of independent experiments is N = 6 whereby nuclear extracts were from 16HBE14o (N = 5) and T84 (N = 1). The blot shown is an exemplificative blot out of the 6 performed replicates (source data is provided with this manuscript). (**B**) Signals obtained with rs152731 probes and an antibody directed against ESRP2 were compared to signals obtained when IgG was used instead of the specific antibody. Data from such paired samples are shown from six independent experiments that differ concerning nuclear extract and electrophoresis conditions. Signals labelled “ESRP2/rs152731” in Fig. 6A. Could be distinguished clearly from the DNA–protein-complex labelled “mixed” in three experiments. Normalized data obtained for rs152731-C and rs152731-T probes were compared by Mann–Whitney U test whereby signals for high molecular weight complexes observed for rs152731-T were stronger (*p* = 0.013). (**C**) 150 µg nuclear extract from T84 cells was incubated with 500 nmol dsDNA probes for SCNN1B SNP rs152731 allele C and SCNN1B SNP rs152731 allele T in an EMSA experiment of a total reaction volume of 40 µl for 3 h at 37 °C. Protein–DNA-complexes were captured with an antibody directed against biotin (ab19221, Abcam, Cambridge, UK), or with IgG as a negative control (indicated by “IP”), and precipitated using protein-G-agarose-beads at 4 °C for 16 h. The supernatant was discarded and proteins were dissociated from agarose beads using Laemmli buffer in two elution steps (indicated by “Elu.” 1st and 2nd) of 30 µl each (10 min, 50 °C), loaded on a denaturing 11% polyacrylamide gel and transferred to a protean supported membrane with 20 µM pore size by tank-blot for 50 min at 100 V. ESRP2 immunoreactive bands were visualized with an antibody directed against ESRP2 (ab113486, Abcam, Cambridge, UK). A lane loaded with nuclear extract only was used as a control to identify ESRP2 (predicted molecular weight: 77 kDa). The number of independent experiments is N = 3 whereby nuclear extracts were from T84. The blot shown is an exemplificative blot out of the 3 performed replicates (source data is provided with this manuscript). (**D**) In the example shown in (**C**), ESRP2 signals derived from the probe with rs152731 allele C correspond to 85% of the signal observed for rs152731 allele T. Two replicate experiments, not shown here, have demonstrated 0% and 65% intensity on the allele rs152731-C, respectively. While the C-to-T-ratios observed in three co-IP experiments were highly variable, these three experiments consistently indicate that rs152731-C binds ESRP2 less tightly than rs152731-T.

ESRP2 was detected as a factor binding to the C to T SNP rs152731 by EMSA-PSeq (Fig. 4) by using polyacrylamide gels with a separating distance of 4 cm. To detect ESRP2-rs152731 binding complexes, we employed EMSAs using polyacrylamide gels with a separating distance of 20 cm (Fig. 6A,B). To exclude nonspecific binding, signals obtained with an antibody directed against ESRP2 were compared to protein-DNA-complexes probed with IgG (isotype control). Signal intensities for rs152731-C were comparable in IgG and anti-ESRP2-Ab lanes. In contrast, signals obtained for rs152731-T were stronger with anti-ESRP2-Ab than with IgG. Normalized signal intensities for rs152731-T were higher than those for rs152731-C (*p* = 0.013).

Next, we addressed whether ESRP2 recognizes rs152731 directly. We used a coimmunoprecipitation (co-IP) experiment using a biotinylated rs152731 EMSA probe as bait with a raw nuclear extract. After precipitation with an anti-biotin-Ab, detection of ESRP2 by Western blotting in co-IP samples verified that all components sufficient for ESRP2 to bind rs152731 are present within the nuclear extract and/or the components used for the EMSA preceding co-IP (Fig. 6C,D). In three independent co-IP experiments comparing the two contrasting rs152731 alleles, ESRP2 signals derived from probes with rs152731-T yielded stronger signals than probes for rs152731-C. In summary, the results from two different techniques confirmed that rs152731-T binds ESRP2 better than does rs152731-C. Thus, the allele rs152731-T associated with intrapair discordance (Tables 1, 2) attracts ESRP2 to a greater extent and is more vulnerable to its genetic variations (Fig. 5).

### ESRP2 knockdown alters global SCNN1B expression in 16HBE14o-cells

**Figure 7:**
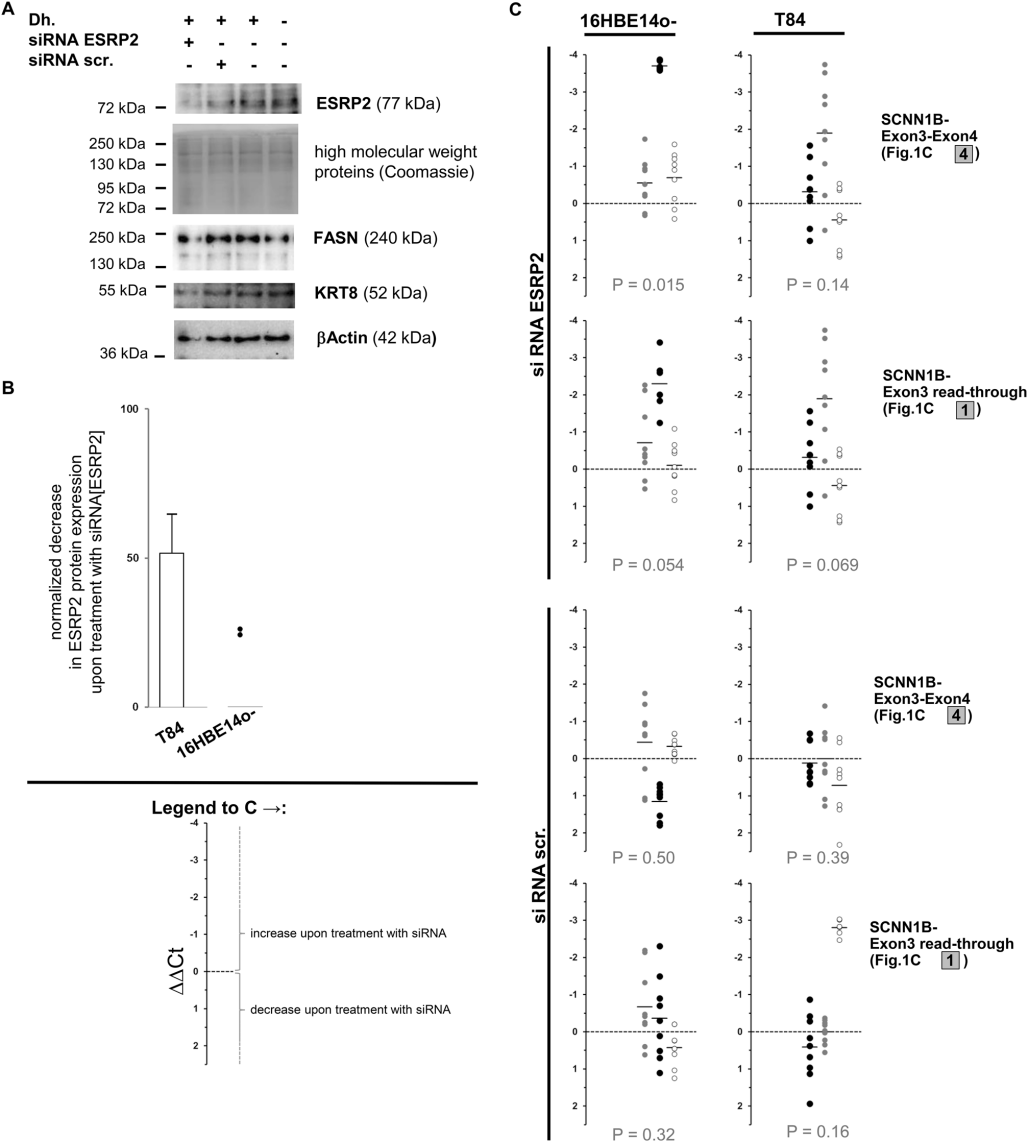
siRNA-mediated downregulation of ESRP2 affects the SCNN1B transcript spectrum in 16HBE14o-cells. siRNA directed against ESRP2 (siRNA ESRP2; mix of four siRNAs, on-target plus pool, GE Healthcare) or scrambled control (siRNA scr., GE Healthcare); was provided to T84 and 16HBE14o-cells. ESRP2 protein expression and SCNN1B mRNA expression levels were compared between cells supplied with the transfection agent Dharmafect 1 (Dh.) only versus cells that received Dharmafect and siRNA directed against ESRP2. Experiments in both cell lines were conducted from two (T84) or three (16HBE14o) different passages as biologically independent replicates whereby in each of these experiments, cell culture plates were treated in parallel for the following conditions: growth control (no Dharmafect transfection agent or siRNA; 8 plates), transfection agent control (with Dharmafect but without siRNA; 8 plates), treated with siRNA directed against ESRP2 (8 plates) and treated with scrambled siRNA (8 plates). Four out of eight plates were harvested at 24 h post-transfection and the remaining four plates were analyzed after 48 h post-transfection. Nuclear extracts were analyzed for ESRP2 protein expression from one plate in these sets of four (**A**,**B**). RNA was isolated from the remaining three plates (**C**). (**A**) Nuclear extracts derived from 16HBE14o-cells treated with 100 pmol siRNA directed against ESRP2 (siRNA ESRP2) or scrambled control (siRNA scr.), transfection control and growth control were analyzed for ESRP2 protein by western blot. The intensity of Coomassie-stained high molecular weight bands was used as a western blot loading control. Signals for fatty-acid synthase (FASN), keratin 8 (KRT8) and βActin are provided as control detections. (**B**) Reduction of protein expression levels of ESRP2 by siRNA directed against ESRP2 was judged from nuclear extracts. All but two sample sets from 16HBE14o-showed very low or absent ESRP2 protein signals and were excluded from quantitative analysis. (**C**) SCNN1B wild-type (exon3–exon4, see Fig. 1C, amplicon 4) and read-through alternative transcript (see Fig. 1C, amplicon 1) were visualized from cDNA derived from control and siRNA-treated cells. Data was evaluated based on the threshold cycle Ct provided for an SCNN1B amplicon in comparison to the housekeeping gene aldolase from technical duplicates for each qPCR reaction. Independent experiments derived from 12 cell culture plates, three each for the conditions growth control (no Dharmafect or siRNA), transfection control (Dharmafect only), siRNA ESRP2 and siRNA scr. were included into the analysis if at least two out of three samples from all conditions gave valid results for all three amplicons (n = 3 for T84 and n = 3 for 16HBE14o). All samples for 16HBE14o 24 h after transfection had to be excluded as SCNN1B transcripts could not be detected in the majority of qPCR reactions. ΔCt values were calculated for each sample as Ct[SCNN1B]-Ct[aldolase]. To compare differential expression between two triplicate sets, ΔΔCt was calculated for all possible nine combinations comparing three case and three reference samples. To assess the influence of Dharmafect, ΔΔCt[Dharmafect] was calculated as ΔΔCt[Dharmafect] = ΔCt[growth control] – ΔCt[transfection control]. The influence of an siRNA ΔΔCt[siRNA ESRP2] and ΔΔCt[siRNA scr.] treatment was calculated as ΔΔCt[siRNA ESRP2] = {ΔCt[siRNA ESRP2] – ΔCt[transfection control] – {mean ΔΔCt[Dharmafect]} and ΔΔCt[siRNA scr.] = {ΔCt[siRNA scr.] – ΔCt[transfection control]} – {mean ΔΔCt[Dharmafect]}. ΔΔCt[siRNA ESRP2] and ΔΔCt[siRNA scr.]. qPCR Data is shown for three independent experiments in each cell line (N = 3) whereby values derived from one biological replicate are displayed using the same color in black, grey or white circles, respectively. *p* values were calculated by Wilcoxon signed-rank test to test against the hypothesis that equal proportions of samples show an increase or decrease of SCNN1B transcript species upon treatment with siRNA. Only unrelated values—i.e. ΔΔCt derived from unshared ΔCt values within biological replicates—were included into the rank test. Significance in this test indicates a systematic effect of siRNA on SCNN1B transcription.

To determine whether ESRP2 influences the transcript species generated from SCNN1B, we downregulated ESRP2 by siRNA in T84 and 16HBE14o-cells and quantified the wild-type and the alternative transcripts by qPCR (Fig. 7). Silencing of ESRP2 in T84 cells resulted in highly variable changes in SCNN1B transcripts. Moreover, the observed changes were comparable to those using scrambled control siRNA. In contrast, no systematic effect of scrambled control siRNA was detected in 16HBE 14o-cells (*p* = 0.50 for wild-type, *p* = 0.32 for alternative SCNN1B transcript; Wilcoxon signed-rank test). Moreover, the amounts of wild-type and alternative SCNN1B were increased in 16HBE14o-cells upon treatment with siRNA directed against ESRP2 (*p* = 0.015 for wild-type; *p* = 0.054 for alternative SCNN1B transcript). Comparison of changes in wild-type and alternative SCNN1B transcript levels assessed by paired ΔΔCt levels for both amplicons indicated that downregulation of ESRP2 induced expression of functional wild-type SCNN1B in 16HBE14o-cells (*p* = 0.081, Wilcoxon signed rank test).

The upper respiratory tract is the origin of 16HBE14o-cells while T84 cells are derived from intestinal cells; 16HBE14o-cells are virus-immortalized and T84 are colon cancer cells. Because ESRP2 plays a prominent role in cancer^18,19^, it is not surprising that these two cell lines behaved differently in the ESRP2 gene silencing assay. Furthermore, the baseline expression of both SCNN1B transcripts was lower in 16HBE14o than in T84 cells. Interestingly, T84 cells carry only one, but 16HBE14o-cells carry two of the ESRP2-receptive alleles T–G–A–C at markers rs152731–rs152745–rs152744–rs152741. This may explain the observed differences between 16HBE14o and T84 cells in response to ESRP2 silencing. Homozygosity for T–G–A–C is associated with intrapair discordance among CF twins and siblings.

## Discussion

The design of the European CF twin and sibling study was inspired by Risch and Zhang^22^ who proposed that the use of sibling pairs with extremely concordant or discordant phenotypes will advance the discovery of quantitative trait loci in humans^22–24^. Due to the high power of this approach, it was estimated that the genotyping load for studies undertaken with an extreme sib-pair design, selecting for patient pairs who exhibit phenotypes below the 30th or above the 70th centile, can be reduced by up to 40-fold^22^. Based on this strategy for patient recruitment, 37 F508del-CFTR homozygous sibling pairs of 318 cystic fibrosis affected patient pairs were selected for the association study by a ranking algorithm^12^. The selected sibling pairs were comparable in terms of their birth cohort^11^. This strategy helped us to minimize the influence of a major nongenetic confounder^25^, i.e., complex therapeutic management which has improved the life expectancy of CF patients by several decades. Pulmonary and gastrointestinal disease manifestations were assessed quantitatively by CF population centiles for the normalized forced expiratory volume in 1 s (FEV1) and by weight as a percentage of predicted weight for height. For these two parameters, we selected sibling pairs with extreme phenotypes in the upper and lower 25%^17,26^. Our selection criteria were in line with recommendations proposed by Risch and Zhang^22^, however, these criteria resulted in a small study population, thus limiting the power of the genetic association study. Moreover, since our study population is of white European descent, a group in which F508del-CFTR is the most common mutation causing CF, we cannot be certain that our findings can be applied to other populations.

**Figure 8:**
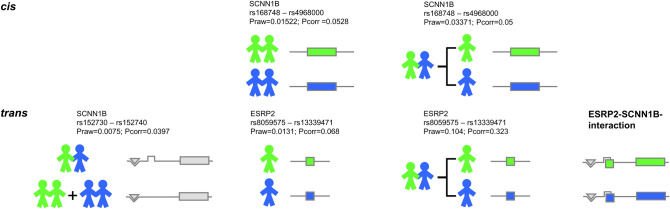
Identifying *cis* and *trans* regulatory elements in SCNN1B. Concordant mildly (two siblings who are shown in green), concordant severely (two siblings who are shown in blue) and discordant siblings (pairs composed of one green and one blue colored sib) have been selected from a total of 318 sibling pairs^12^ and analyzed in a series of case-reference association studies. *Cis*: The genomic segment rs168748–rs4968000 determines SCNN1B functionality directly: the observed interpair and matching intrapair association with CF disease severity can be explained by the SCNN1B variants *in cis* (data not shown). *Trans*: To explain the association with the discordant phenotype, variants at SCNN1B genomic segment rs152730–rs152740 require an interacting partner encoded *in trans* to SCNN1B. The genetic variation at ESRP2 contributes to the manifestation of SCNN1B functionality. In this model, binding sites for DNA interacting partners are shown as a triangular groove (for interaction partners shaped as a triangle) and as a square groove (for interaction partners shaped as a square). In the context of this work, ESRP2 is represented by a square and fits into its binding side at the SCNN1B locus. As a consequence, the phenotype of a sib within a discordant pair is shaped by the interaction of ESRP2 and SCNN1B: the allele rs152731-T, associated with intrapair discordance (Tables 1,2), is more vulnerable to ESRP2 (Fig. 6) and its genetic variations (Fig. 5). Functionally divergent ESRP2 alleles, visualized as green and blue squares, thus determine the phenotype of carriers with ESRP2-receptive SCNN1B alleles that are shown with a square groove. These ESRP2-receptive SCNN1B alleles include rs152731-T and are overrepresented among discordant, but rare among concordant pairs. The five displayed association signals correspond to: SCNN1B, rs168748–rs4968000, Praw = 0.01522; Pcorr = 0.0528: interpair comparison of allele distribution between 11 concordant mildly and 10 concordant severely affected sibling pairs (data not shown). SCNN1B, rs168748–rs4968000, Praw = 0.03371; Pcorr = 0.05: intrapair comparison of allele distribution between mildly affected siblings and severely affected siblings from discordant pairs (data not shown). SCNN1B, rs152730–rs152740, Praw = 0.0075; Pcorr = 0.0397: interpair comparison of allele distribution between 14 discordant sibling pairs and 21 concordant sibling pairs (see Fig. 1B). ESRP2, rs8059575–rs13339471, Praw = 0.0131; Pcorr = 0.068: comparison of allele distribution of unrelated patients stratified for the response of the nasal epithelium upon superfusion with amiloride assessed by nasal potential difference measurement (see Fig. 5). ESRP2, rs8059575–rs13339471, Praw = 0.104; Pcorr = 0.323: intrapair comparison of allele distribution between mildly affected siblings and severely affected siblings from discordant pairs.

Risch and Zhang concluded from their simulation studies that “extremely discordant sibling pairs represent a powerful design for the association studies of candidate genes”^22^, and our findings fully support this idea. The use of sibling pairs with extreme clinical phenotypes has been applied before^27,28^, and our data support the notion that gene–gene interactions mediated by factors encoded *in trans* of the studied locus can be distinguished in an association study when mildly and severely affected siblings of discordant pairs are compared (Fig. 8).

Hence, in this study we identified a haplotype within SCNN1B associated with intrapair discordance in CF sibling pairs (Fig. 1, Tables 1, 2). We investigated in silico (Fig. 2) and experimentally (Figs. 3, 4) the occurrence of DNA-binding proteins interacting differentially with single or multiple SNPs within the haplotype. We were able to recognize ESRP2 as a candidate for validation among nucleic acid binding proteins, which showed an association with SCNN1B/ENaC function (Fig. 5). We further employed EMSA and co-IP as two different experimental approaches to support the allele-dependent interaction between rs152731 in SCNN1B and the nucleic acid binding protein ESRP2 (Fig. 6). Moreover, we demonstrated that siRNA mediated silencing of ESRP2 in respiratory epithelial cells causes an alteration in global SCNN1B expression (Fig. 7). Altogether, our findings consistently support the idea that SCNN1B and ESRP2 are interacting partners and that ESRP2 is capable of altering the SCNN1B transcript repertoire. It is plausible that this interaction can alter ENaC function and has an influence on the manifestation of CF.

Our proof-of-principle study has several limitations. Specifically the findings cannot fully explain whether the regulatory element within *SCNN1B* leads to the alternative SCNN1B transcript, from which a severely truncated, 221 amino acid SCNN1B may be translated. Similar truncated SCNN1B isoforms of 217 and 306 amino acids have been observed in patients with systemic pseudohypoaldosteronism^48^. In addition, heterologous expression of these truncated SCNN1B mutants in *Xenopus* oocytes showed that they can assemble with wild-type alpha- and gamma ENaC subunits^48^. These SCNN1B mutants resulted in lower ENaC activity (by 3–7%) than the wild-type protein^48^. Under physiological conditions, parallel expression of two SCNN1B transcript species, one of which yields a truncated SCNN1B isoform upon translation, can reduce SCNN1B function but the extent remains unclear.

Using EMSA-Pseq for the identification of DNA-binding proteins has previously been conceived^29–31^. To examine whether our EMSA conditions allowed the formation of high-molecular-weight multiprotein complexes with coherent DNA–protein-interactions in vivo, we investigated protein-DNA-complexes using an NFkappaB-P65-consensus as bait. In line with published data^32–35^, this probe attracted NFkappaB-p65 and its known interaction partners, such as STAT3 and STAT6. From the EMSA-Pseq of SCNN1B probes, we selected ESRP2 as a candidate for further validation experiments. This selection was based on the fact that this protein is characteristically expressed in epithelial cells and that similar to other SNP-specific proteins recognized by EMSA-PSeq, ESRP2 has been well-characterized as an RNA-binding protein^18,19^.

The defining border between RNA- and DNA-binding proteins has recently softened because typical DNA-binding proteins have become known to target long noncoding RNAs, defining dual-recognition nucleic acid binding proteins^36,37^. A growing number of nucleic acid binding proteins have been recognized to interact with both nucleic acid species^37–44^ and genomic DNA^45^. The ability to bind to DNA and RNA simultaneously designates a dual recognition protein capable of shuttling between both nucleic acid types during transcription^38^. During transcription, DNA and RNA are physically close, and therefore, cotranscriptional processes, such as pre-mRNA splicing, can be mediated by putative dual-recognition proteins, such as hnRNP splicing regulatory factors^46,47^.

Nevertheless, we cannot exclude that SCNN1B may have other important interacting partners in addition to ESRP2 that were not discovered in this study. In this work, we analyzed only three of six SNPs by EMSA-PSeq. Furthermore, while we filtered our primary protein sequencing data using a positive control (NFkappaB-P65) and several technical controls to recognize contamination, we did not incorporate a protein–probe-interaction with low binding affinity, which might enable the detection of weak interacting partners. In the future, the resolution of the EMSA-Pseq can be improved by using scrambled probes to control for nonspecific binding and by incorporating the false-positives captured in the data evaluation strategy. Moreover, EMSA-PSeq utilizes mass spectrometry to identify proteins. In contrast, nucleic acids such as long noncoding RNAs with the potential of interacting with the DNA sequence cannot be identified in this experimental setting, and thus, their relevance needs to be verified by other methods.

The haplotype associated with intrapair discordance covers a genomic segment of 8 kb, implying that a synergistic relationship of more than one interaction partner is responsible for the selective advantage that underlies the maintenance of linkage disequilibrium over such a distance. Thus, it is unlikely that the SCNN1B function can be fully understood based on studying single SNPs. Regardless, we are convinced that the methodology proposed herein—analysis of clinically discordant sibling pairs in combination with EMSA-PSeq—aids in our understanding of how some of the 10,000 SNPs identified by GWAS as being meaningful (www.genome.gov/gwastudies. Accessed at 03.02.2015) contribute to the manifestation of phenotypes in humans. As gene–gene interactions have been suggested to account for the phenomenon termed “missing heritability”^49^, the discovery of regulatory interactions such as those between ESRP2 and *SCNN1B* might help to annotate existing GWAS data sets that have been performed with sibling pairs^50–52^.

## Methods

Details on the experimental procedures are provided in the supplement.

### Cell culture

Biomaterials were derived from T84 colon cancer cells and immortalized respiratory epithelial 16HBE14o-, CFBE41o- and CFTE29o-cells.

### RNA preparation

For RNA isolation, cells were cultured in plates, grown to confluency, snap-frozen in the gaseous phase of liquid N_2_ and stored at − 80 °C. RNA was extracted using QIAamp RNA Blood Mini Kit (52,304, Qiagen, Hilden, Germany) and RNase-free DNase Set (79,254, Qiagen, Hilden, Germany).

### Oligonucleotides for PCR

Sequences of primers used for genotyping and combinatorial PCR are listed in the supplement (SupplTab 5).

### Extraction of nuclear proteins

Nuclear extracts were prepared according to published standard methods^53^ (SupplTab 6). To prevent carryover of the high-salt buffer used for lysis of nuclei, nuclear proteins were dialyzed against low-salt HEPES buffer. The completeness of dialysis was verified by measuring the conductivity of the nuclear extract with a needle probe (customized, Technische Forschungswerkstätten of the Hannover Medical School). The quality of nuclear proteins was ascertained by verifying the conductivity of the final extract after dialysis and by noting the absence of degradation by SDS-electrophoresis followed by Coomassie staining.

### EMSA-PSeq

To identify proteins that interact with a specific DNA sequence, we performed an EMSA experiment, visualized the shifted band, captured the DNA–protein complexes by excising the corresponding region of the polyacrylamide gel and then performed protein mass spectrometry. The composition of the EMSA binding buffer was adjusted to reflect the nuclear milieu^54^ (SupplTab 7). All experimental details and data analysis methods are provided in the supplement (SupplTab 8, SupplTab 9, SupplFig4, SupplFig5, SupplFig6).

### Coimmunoprecipitation

Biotinylated 35-mer double-stranded DNA probes for the rs152731 allele C and allele T were incubated with nuclear extract in an EMSA experiment. The DNA–protein-complexes were precipitated using an anti-biotin antibody and protein G agarose beads. The protein–DNA-complexes were eluted from the beads in three consecutive steps, and Western blotting with anti-ESRP2 was used to detect ESRP2 in the precipitated protein–DNA-complexes. IgG instead of the anti-biotin antibody served as a negative control in all experiments. ESRP2 was identified using a signal from an unpurified nuclear extract developed in parallel in each Western blot experiment.

### siRNA-mediated downregulation of ESRP2 in epithelial model cell lines and SCNN1B transcript analysis by real-time RT-PCR

siRNA directed against ESRP2 and scrambled control siRNA was purchased from GE Healthcare (mixture of four siRNAs, on-target plus pool, GE Healthcare). T84 and 16HBE14o-cells were transfected for 24 h and 48 h using a protocol supplied by the manufacturer with 10 µl of Dharmafect 1 and 100 pmol siRNA in 2 ml of cell culture medium per well of a 6-well plate. Commercially available kits were used according to the manufacturer’s instructions. RNA was isolated using the RNA-easy-mini-kit (Qiagen), transcribed into cDNA with the High-Capacity cDNA Reverse Transcription kit with RNase inhibitor (Applied Biosystems) and used as a template for real-time PCR with PowerUp SYBR Green Master Mix (Applied Biosystems) to target wild-type and read-through alternative SCNN1B transcripts with the StepOnePlus real-time PCR system (ThermoFisherScientific). The housekeeping gene aldolase was amplified from 5 ng cDNA with 400 nM forward and reverse primers. SCNN1B transcripts were amplified from 30 ng of cDNA with 400 nM (read-through alternative transcript) and 700 nM (wild-type transcript) forward and reverse primers, respectively. Amplification was carried out using annealing at 60 °C. Threshold cycle (Ct) values were retrieved using StepOne-Software (Thermo FisherScientific).

### Genetic markers

Except for 7 previously typed markers^11^, genetic markers were developed de novo for this project. Genotyping was performed by the SNPstream assay (technology by Beckman Coulter, used at Cologne Center of Genomics, Cologne, Germany), by microsatellite genotyping using direct blotting electrophoresis^17^ or by PCR–RFLP (see SupplTab 5a).

### Evaluation of genetic data in the association study on European cystic fibrosis twins and siblings

The work presented here derived data from an association study on European CF twins and siblings^17^. The study was approved by the ethics committee of Hannover Medical School and written informed consent was obtained from all participants or their parental guardians. All methods were performed in accordance with relevant guidelines and regulations. The clinical characteristics of the patients have been described in detail elsewhere^12,15,17^. Briefly, the 12% most informative pairs from the entire sample of 318 CF twin and sibling pairs for whom pulmonary function data and weight and height were available in 1996 were selected by a ranking algorithm^12^. To study genetic modifiers, we aimed to reduce the effect of the disease-causing CFTR gene on the disease phenotype, thus deciding to study only one CFTR mutation genotype. F508del-CFTR, present on 70% of CF chromosomes from white populations of Central and West-European countries, is the only CFTR mutation for which such an approach is feasible. Moreover, patient subsamples were examined to assess the manifestation of the basic defect of impaired ion conductance in the respiratory tissue, as determined in vivo by nasal potential difference measurement^15^, and in intestinal tissue, as determined ex vivo by intestinal current measurement^15^. Genetic information obtained from the case and reference populations with contrasting phenotypes was compared using the software package FAMHAP^55^, which allows family-based analysis^56,57^, accepts data evaluation in association studies on unrelated individuals as well as on affected sibling pairs^55^ and is adapted to handle intrapair comparison of genotype data in sibling pairs^55^. Correction for multiple testing at loci typed with more than one marker was performed by haplotype permutation^56^. For this purpose, the entire data set of cases and references was used to estimate haplotype frequencies^55^. To ensure a consistent assignment of rare haplotypes in small subsamples, the genotype data of 101 families with a total of 171 patients from the European CF twin and sibling study were used as a training set in all comparisons. Haplotype, or, in cases of noninformative phase or haplotype uncertainty, weighted haplotype explanation lists were assigned to each individual whereby the haplotype frequencies of the entire data set were taken into account to compute conditional likelihood weights^55^. Permutation was performed by randomly assigning the affection status to the individuals in each replication^55^. For the comparison of case sibling pairs to reference sibling pairs, the affection status was permuted or not with an equal chance for both siblings simultaneously^55–57^. For all comparisons described herein, the phenotypes and sample sizes of the case and reference populations are detailed within the legends, in Figs. 1, 5 and 8 as well as in Table 1.

### Statistical analyses

The algorithms of Sham and Curtis^58^ were used to compare the observed occupancy of SCNN1B haplotypes associated with concordance vs discordance with unique transcription factors to the expectancy value derived from binding partners distributed equally between both haplotypes.

The EMSA band intensity between rs152731-C and rs152731-T probes was compared using a Mann–Whitney-U-Test in technically (electrophoresis) and biologically (cell culture and preparation of nuclear extract) independent experiments.

Changes in the expression levels of SCNN1B transcripts were judged from threshold cycle Ct values obtained by qPCR using the ΔΔCt method comparing siRNA or treated cells to untreated controls. To test against the hypothesis that no change in the SCNN1B transcript was observed, the Wilcoxon signed rank test was used to assess whether or not equal proportions of samples showed an increase or decrease in SCNN1B transcript species upon treatment with siRNA. For this analysis, technically (qPCR) and biologically (cell culture and experimental intervention) independent Ct values were used. For statistical analysis, ΔΔCt values derived from independent siRNA-treated or control samples were used.

## Supplementary Information

Supplementary information 1.

Supplementary information 2.

Supplementary Data R1.

Supplementary Data R2.

Supplementary Data R3.

## Data Availability

Supplemental Information is provided with this manuscript. Primary data will be shared with interested parties upon reasonable request.
