# Supplementary material for: Genetic information from discordant sibling pairs points to ESRP2 as a candidate trans-acting regulator of the CF modifier gene SCNN1B
Source: Sci Rep. 2020 Dec 31;10:22447. doi: 10.1038/s41598-020-79804-y (PMC7775467; doi:10.1038/s41598-020-79804-y)
Supplement: Supplementary file 1 — Supplementary information 1. [file 41598_2020_79804_MOESM1_ESM.docx]

**Supporting information for:**

**Genetic information from discordant sibling pairs points to ESRP2 as a candidate trans-acting regulator of the CF modifier gene SCNN1B**

Tim Becker, Andreas Pich, Stephanie Tamm, Silke Hedtfeld, Mohammed Ibrahim, Janine Altmüller, Nina Dalibor, Mohammad Toliat, Sabina Janciauskiene, Burkhard Tümmler, Frauke Stanke

**raw data:**

**xls supplement R1** (see Supplementary Figure 5 for description)

**xls supplement R2** (see Supplementary Figure 5 for description)

**xls supplement R3** (see Supplementary Figure 5 for description)

**Source data for Figure 6** is provided separately as pdf.

**Supplementary results & supplementary methods**

**Supplementary results**

**A. In silico analysis of contrasting alleles on SCNN1B genomic fragment rs152730-rs152741**

**B. SCNN1B alternative transcript**

**C. Predicted interaction partners of contrasting haplotypes on SCNN1B genomic fragment rs152730-rs152741**

**D. Electrophoretic mobility shift assay – Protein Sequencing (EMSA-PSeq)**

**Supplementary methods**

**E. Cell culture**

**F. Nucleic acid analysis**

**G. Electrophoretic mobility shift assay – Protein Sequencing (EMSA-PSeq)**

**Supplementary references**

**A. In silico analysis of contrasting alleles on *SCNN1B* genomic fragment rs152730-rs152741**

We wanted to know whether these six variants have an influence on the pre-mRNA structure, and therefore we used the RNAsnp webserver to compare the RNA structures of both alleles at these six single nucleotide variants (available at <http://rth.dk/resources/rnasnp/submit.php#input>, accessed on 01/2016;^1,2^). While SNPs rs152730, rs152731, rs152745 and rs152744 did not alter the predicted secondary structure of the pre-mRNA, both rs152741 and rs152740 have predicted capabilities to change the local RNA secondary structure (SupplTab. 1, SupplTab2). Using RBPmap, embedded in the UCSC browser, we have asked for RNA binding proteins that have predicted binding sites at or on our SNPs of interest. Putative bindings sites for RNA-binding proteins have been predicted for rs152730, rs152731 and rs152744 (SupplFig 1).

**Supplementary Table 1:** Influence of SNPs rs152730, rs152731, rs152744, rs152745, rs152741 and rs152740 on RNA structure by RNAsnp

| SNP | Coordinate on GRCh37.p13 | p-value (comparison of structures between both alleles) |
| --- | --- | --- |
| rs152730 | chr16:T23361021G | 0.5046 |
| rs152731 | chr16:T23361982C | 0.5495 |
| rs152745 | chr16:G23366422A | 0.8912 |
| rs152744 | chr16:A23367580G | 0.5508 |
| rs152741 | chr16:C23368760T | 0.1377 |
| rs152740 | chr16:A23369090T | 0.0834 |

**Supplementary Table 2:** Comparison of local RNA secondary structure for both alleles, displayed in contrasting colours red and green, at rs152730, rs152731, rs152745, rs152744, rs152741 and rs152740 retrieved from RNAsnp

| SNP | Predicted RNA secondary structure for allele A | Predicted RNA secondary structure for allele B |
| --- | --- | --- |
| rs152730 | U23361021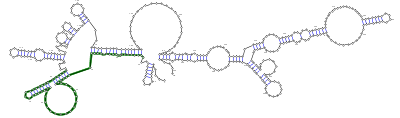 | G23361021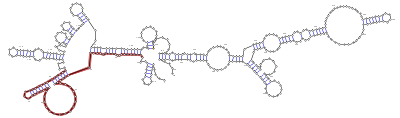 |
| rs152731 | U23361982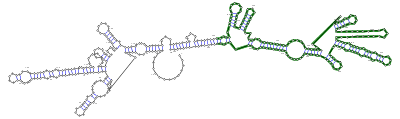 | C23361982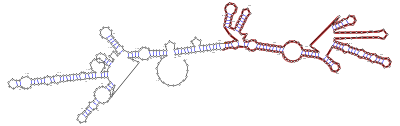 |
| rs152745 | G23366422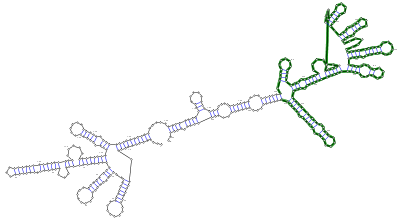 | A23366422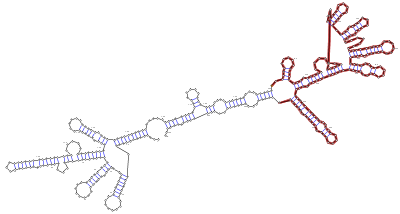 |
| rs152744 | A23367580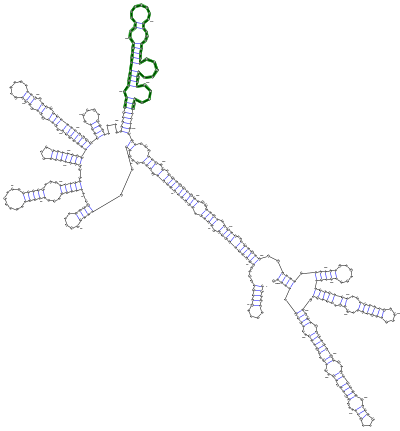 | G23367580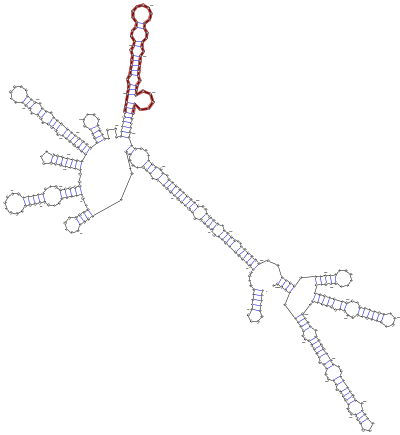 |
| rs152741 | C23368760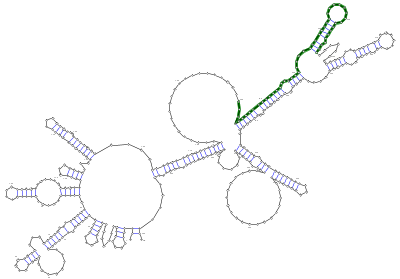 | U23368760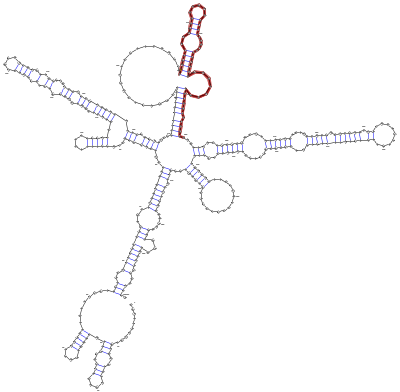 |
| rs152740 | A23369090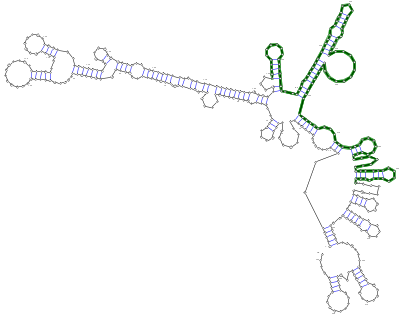 | U23369090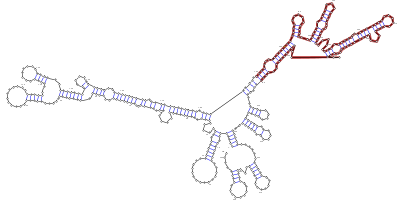 |

**Supplementary Figure 1:** Prediction of interaction sites near RNA binding proteins by RBPMap on rs152730 (A), rs152731 (B) and rs152744 (C), localized centrally on the physical map in each of the genomic segments represented within the subpanels. No interactions were predicted by RBPmap for SNPs rs152745, rs152741 and rs152740.

**B. SCNN1B alternative transcript**

**Supplementary Figure 2:** Combinatorial PCR detects an intronic sequence within an alternative SCNN1B transcript

A: Expressed sequence tags (ESTs) mapped to the region of interest between rs152730 and rs152740 derived from the Ensembl genome browser^3^ (accessed at http://www.ensembl.org/; 10/2013).

B: Position of primers used for combinatorial PCR in exons of the reference sequence (black) or ESTs (red).

C,D: Amplification of an alternative SCNN1B transcript generated by exon read-through of SCNN1B exon 3. Templates are: C – cDNA derived from T84 cells; R: untranscribed RNA derived from T84 cells; D: genomic DNA; H: H_2_O (negative control). DNAse treatment in lane 6 and 11 was performed to eliminate contaminating DNA as specified (see D). In lane 10, indicated by “Y”, DNAse was used to treat a water sample and 50 ng of DNA were provided after DNAse inactivation to demonstrate that the left-over or reconstituted activity of 5 U DNAse does not prevent template amplification when the material is used immediately for PCR after inactivation of DNAse. PCR primers used were: a – see Fig. 1C, there indicated as “amplicon 5”, primers located within exon 1 encoding the 5’ UTR of SCNN1B and in exon 4, expected size for spliced product 946 bp; b – see Fig. 1C, there indicated as “amplicon 1”, primers located within exon 3 and on the following intron segment present in ESTs, expected size of 280 bp; c – control PCR product generated from genomic DNA of similar size than expected for PCR b in lanes 5 and 6. Size markers used for calibration of SCNN1B transcripts in lanes 1, 4 and 9 are PCR products derived from genomic DNA with primers unrelated to the work reported upon in this manuscript.

E: Alignment of the alternative transcript to the genomic reference sequence. The PCR product was derived from T84 cDNA and provided as Query_98509 to NCBI_BLAST. This zoomed view shows that the mRNA, and the cDNA derived thereof, was generated by a direct exon read-through event of SCNN1B exon 3 and contains the genomic reference sequence at the exon 3/intron 3 border by the base.

**C. Predicted interaction partners of contrasting haplotypes on *SCNN1B* genomic fragment rs152730-rs152741**

For each of the 13 predicted interaction partners, one microsatellite motif was tested for informativity on five DNA samples of non-CF controls. Motifs tested for *JUN*, *OCT1*, *MYB*, *ER*, *GATA3*, *FOXJ2* and *SP1* did not show a notable length variation, hence these genes could not be investigated based on the single selected motif. Informative markers that were tested for association with CF disease severity or the manifestation of the basic defect in nasal or intestinal tissue on the samples from the European CF twin and sibling study^4^ were obtained for predicted interaction partners *GFI1*, *GATA2*, *PAX6*, *HNF1*, *FOS* and *MAFF*.

**Supplementary Table 3a:** Transcription factors predicted to specifically interact with TTGACA, associated with discordance, at rs152730-rs152731-rs152745-rs152744-rs152741-rs152740

| Transcrip-tion factor |  | SNP | matrix identifier | pos | strand | core match | matrix match | sequence - (+) strand is shown |
| --- | --- | --- | --- | --- | --- | --- | --- | --- |
| Gfi-1 | growth factor independent 1 transcription repressor | rs152731 | V$GFI1_01 | 9 | (+) | 0.960 | 0.915 | acgcctgtAATCTcagcattttga |
| AP-1 | JUN; avian sarcoma oncogene homolog | rs152745 | V$AP1_Q4 | 16 | (-) | 0.935 | 0.877 | ttgtGGTCAgc |
| Oct-1 | POU2F1; POU class 2 homeobox 1 | rs152730 rs152744 | V$OCT1_Q6  V$OCT1_Q6 | 16  12 | (-)  (+) | 0.792  0.755 | 0.657  0.576 | acagTTAACaaaaca  tctgaaGCAAGaaca |
| GATA-2 | GATA binding protein 2 | rs152731 | V$GATA2_03 | 14 | (-) | 0.871 | 0.896 | tgTAATCtca |
| v-Myb | myeloblastosis viral oncogene homolog | rs152730  rs152730 | V$VMYB_01  V$VMYB_02 | 15  16 | (-)  (-) | 0.938  0.907 | 0.890  0.902 | aaCAGTTaac*  aCAGTTaac* |
| ER | ER-alpha, estrogene receptor | rs152745 | V$ER_Q6 | 17 | (-) | 1.000 | 0.875 | tgtGGTCAgcacacagctg |
| Pax-4 | paired box 4 | rs152740  rs152740 | V$PAX4_01  V$PAX4_01 | 6  14 | (-)  (+) | 0.986  0.881 | 0.726  0.615 | ataccctcttcCTTGAgggcc  ttcctTGAGGgccaccccatg |
| GATA-3 | GATA binding protein 3 | rs152731 | V$GATA3_03 | 14 | (-) | 0.945 | 0.892 | tgTAATCtca |
| Pax-6 | paired box 6 | rs152741 rs152740 | V$PAX6_01  V$PAX6_01 | 10  6 | (-)  (-) | 0.831  0.832 | 0.752  0.601 | caaaaattaacCGGGAgtggt  ataccctcttcCTTGAgggcc |
| FOXJ2 | fork head homologous X | rs152730 | V$FOXJ2_02 | 10 | (+) | 0.819 | 0.791 | aatACAACagttaa |
| SP1 | specifity factor 1 | rs152741  rs152741  rs152741  rs152741 | V$E2_01  V$E2_01  V$E2_Q6  V$E2_Q6 | 17  17  17  17 | (+)  (-)  (+)  (-) | 0.918  0.986  0.902  0.984 | 0.934  0.974  0.924  0.964 | taaccgggagTGGTGg  tAACCGggagtggtgg  taaccgggagTGGTGg  tAACCGggagtggtgg |
| HNF-1 | Hepatocyte Nuclear Factor 1alpha | rs152730 | V$HNF1_C | 9 | (-) | 1.000 | 0.687 | aaatacaacagTTAACa |
| AP-1 | FOS; murine osteosarcoma viral oncogene homolog | rs152745 | V$AP1_Q2 | 16 | (-) | 0.962 | 0.936 | ttgtGGTCAgc |
| v-Maf | musculoaponeurotic fibrosarcoma oncogene homolog F | rs152731 rs152745 | V$VMAF_01  V$VMAF_01 | 11  12 | (-)  (-) | 1.000  1.000 | 0.703  0.740 | gcctgtaatcTCAGCattt  gatcttgtggTCAGCacac |

* these two interactions were valued as a single event for Fig 2.

**Supplementary Table 3b:** Analysis of genes encoding for transcription factors* predicted to specifically interact with alleles composing the haplotype TTGACA, associated with discordance, at rs152730-rs152731-rs152745-rs152744-rs152741-rs152740 as candidate genes in an association study

| Chr. Pos. | Gene | usability of single tested repetitive element for microsatellite genotyping typing | Findings in association study** |
| --- | --- | --- | --- |
| 1p22 | GFI1 | *de novo* marker informative | Pbest,raw = 0.185 / no genotype-phenotype association |
| 1p32-p31 | JUN | not informative | ⎯ |
| 1q24.2 | OCT1 | not informative | ⎯ |
| 3q21.3 | GATA2 | *de novo* marker informative | Pbest, raw = 0.0456 observed for change in nasal potential upon superfusion with amiloride (indicative for ENaC-mediated sodium transport) |
| 6q22-q23 | MYB | not informative | ⎯ |
| 6q25.1 | ER | not informative | ⎯ |
| 10p15 | GATA3 | not informative | ⎯ |
| 11p13 | PAX6 | *de novo* marker informative | Pbest, raw = 0.0172 observed for change in nasal potential upon superfusion with chloride-free solution (indicative of CFTR-mediated residual function) |
| 12p13.31 | FOXJ2 | not informative | ⎯ |
| 12q13.1 | SP1 | not informative | ⎯ |
| 12q24.2 | HNF1 | *de novo* marker informative | Pbest, raw = 0.0731 observed for change in nasal potential upon superfusion with chloride-free solution (indicative of CFTR-mediated residual function) |
| 14q24.3 | FOS | *de novo* marker informative | Pbest, raw = 0.173 / no genotype-phenotype association |
| 22q13.1 | MAFF | *de novo* marker informative | Praw = 0.0821 for association of disease severity comparing mildly and severely affected sibling within discordant pairs  Praw = 0.0023 for association of DIDS-insensitive residual chloride secretion in excised intestinal biopsies, basic defect assessed by intestinal current measurement (indicative of CFTR-mediated residual chloride secretion) |

* PAX4 is in absolute LD to the CF disease-causing gene CFTR and cannot account for differential carriership of transcription factor variants in discordant sib pairs: both siblings have inherited the same CFTR gene by descent and all adjacent sequences including PAX4^5^. As this study aims to identify regulatory interaction partners of SCNN1B for which discordant siblings carry different alleles, PAX4 was excluded from analysis in this association study.

** all phenotypes previously described for the European CF twin and sibling study^4^ were analyzed

**D. Electrophoretic mobility shift assay – Protein Sequencing**

**(EMSA-PSeq)**

**Principle of the assay**

In order to identify proteins that interact with a specific DNA sequence, we decided to perform an EMSA experiment, visualize the shifted band, capture the DNA-protein-complexes by excision of the corresponding part of the polyacrylamide gel and perform protein mass spectrometry. We here provide data on the method, on the primary data and on how signal specific to DNA-protein-complexes and noise was discriminated in our experimental setup.

**EMSA-PSeq samples and negative controls**

Briefly, we evaluated a total of 25 EMSA-PSeq datasets derived from samples captured with ds DNA probes corresponding to the p65 consensus motif (one sample), duplicate samples from both alleles of one SNP within IFNGR1 unrelated to the question addressed within this manuscript^6^, a sample each from both alleles of another SNP within IFNGR1 unrelated to the question addressed within this manuscript^6^, 11 samples derived from both alleles of three SNPs within SCNN1B as specified in SupplTab 9 and seven samples corresponding to negative controls of these experiments. Negative controls refer to excised gel samples processed after electroblotting. These excised negative control gel samples correspond to relative positions of the visualized signals, but are devoid of specific DNA-protein-complexes due to transfer during electroblotting. Specifically, five of the seven control samples were derived from an experimental setup whereby the EMSA was run twice, one half of the samples being transferred onto membranes by electroblotting for visualization of the DNA-protein complexes while the other sample was not subjected to blotting and served as material for protein sequencing.

**Signal-to-noise discrimination in EMSA-PSeq samples**

The mean number of protein groups identified by MASCOT within these samples are 46 [range: 30 – 81] among negative controls and 989 [range: 613 – 1253] among samples derived from high molecular weight DNA-protein complexes. These counts include duplicates such as protein isoforms which are recognized as entities within one protein group. Contaminants such as abundant keratins derived from skin were easily recognized within the datasets as they were present in all samples, including the negative controls. We have also noticed that 14 / 25 EMSA-PSeq datasets (56%) and 17 out of 25 (68%) EMSA-PSeq datasets were positive for the highly expressed double-strand break rejoining DNA repair proteins XRCC5 and XRCC6. XRCC5 and XRCC6 were estimated to occur at 455 ppm and 425 ppm (data accessed at: [www.pax-db.org](http://www.pax-db.org)^7^; derived from data set “colon integrated”), which is just one order of magnitude below keratins which are well-known contaminants in samples analyzed by protein mass spectrometry (reported at 2225 ppm for KRT8). In other words, even though XRCC5 and XRCC6 are DNA-binding proteins and even though these DNA repair proteins are capable to recognize the ends of our dsDNA probes used for the EMSA-PSeq, we cannot be sure that the presence of XRCC5 and XRCC6 in our samples is due to their DNA binding capabilities. More likely, like keratins, they represent an unspecific contamination of highly expressed proteins. With that in mind, we choose to define proteins as contaminants which were

- identified in negative control samples
- identified in the majority of the EMSA-PSeq data sets
- known to be highly expressed (expression levels taken from the protein abundance across organism database paxdb, accessed at [www.pax-db.org](http://www.pax-db.org)^7^)

Criteria were applied hierarchically to the entire dataset (see SupplFig 3): A total of 5622 entries were provided by MASCOT for at least one of the 25 EMSA-PSeq samples. As MASCOT provides data on protein groups, these 5622 entries consist of duplicates (such as multiple isoforms reported upon of one protein). Respectively, the 5622 entries correspond to 1666 proteins and their 3956 isoforms. To remove putative contaminants from this dataset, firstly, all proteins identified in one or more negative control sample were labeled as contaminants (110 out of 1666 proteins; 6%). Next, all remaining entries were substracted for those reported for 4 or more EMSA-PSeq datasets (815 out of 1556 proteins, 52%). Finally, for the remaining 741 proteins, expression levels were retrieved from pax-db. As the nuclear extracts used in the EMSA-PSeq were derived from the colon cancer cell line T84, expression levels were judged based on the dataset “colon integrated” at pax-db (Protein Abundance Across Organisms, available at: www.pax-db.org^7^), reasoning that proteins that are lowly expressed in T84 but have been detected by protein MassSpec in our EMSA-PSeq samples have bound to the dsDNA probe and were enriched by the assay (Raw data: xls supplement R1). To define a threshold for highly expressed and thus putatively contaminating proteins, we relied on findings from our proof-of-principle-experiment with the p65 consensus probe. This sample provided the identification of the expected p65 protein as well as STAT3 and STAT6, both of which are known to recognize DNA in sequence-specific manner REFS and which have been shown to interact with the NFkappaB-p65 protein REFS. These three key proteins are expressed at 76 ppm (RELA alias NFkappaB-p65), 174 ppm (STAT3) and 94 ppm (STAT6, respectively. Proteins reported to be expressed at 200 ppm or more in the set of 741 proteins were excluded from further analysis (146 of 741 proteins; 19%). Findings fulfilling any of these criteria were disregarded irrespectively of the functional annotation of the protein, i.e. regardless of whether or not they are annotated as DNA-binding proteins. In summary, we have employed two consecutive steps to recognize and exclude false-positives within our dataset, removing 110 and 815 entries. Next, we sought to identify true-positives, based on their low expected expression level within T84 cells, and hence nuclear extracts, reducing the complexity of the data set to 595 proteins.

For that purpose, we have looked at the 595 remaining non-abundant proteins, enriched in individual EMSA-PSeq samples, for proteins annotated to bind nucleic acids or to act as a transcription factor. In other words, we have mined the protein sequencing data for proteins that are known to be found within the nucleus, known to bind nucleic acids and known to have low expression levels in order to avoid false-positive calls of interaction partners to the SCNN1B alleles associated with clinical intrapair discordance in cystic fibrosis. Gene ontology terms were extracted from the panther database ([www.pantherdb.org](http://www.pantherdb.org)^8,9^), calling all terms provided for “metabolic function” (MF complete), “biological process” (BP complete) or “cellular compartment” (CC complete). These entries were mined for the presence of the terms "DNA binding", "RNA binding", "regulation of transcription" and "splicing" to identify candidates that might mediate SCNN1B gene regulation. Manual curation of all remaining entries was done to remove proteins that were annotated as being involved by “regulation of transcription” but not annotated as “DNA binding” or “histone binding” or “chromatin binding” as these proteins that elicit their regulatory properties via protein-protein-interaction are unlikely to be responsive to the causative single nucleotide exchanges within SCNN1B. Furthermore, proteins annotated as “RNA binding” but for which the only metabolic function is listed as “translation” were removed. In contrast, we have retained all proteins annotated as “RNA binding” that are involved in pre-mRNA splicing as the intron removal in eukaryotes is a co-transcriptional process whereby the DNA, and hence the SCNN1B SNPs under investigation, are physically close. In the subset of 595 proteins enriched in EMSA-PSeq samples, these ontology-derived criteria were fulfilled by 104 proteins (104 of 595, 17%; raw data: xls supplement R2). Of these 104 probe-specific proteins annotated for nucleic acid binding capabilities, 11 were detected with the p65 consensus probe, and 16, 5 and 8 were attracted by SNPs rs152730, rs152731 and rs152744 within SCNN1B, respectively. These 40 proteins are displayed in SupplTab 4. Primary data obtained from protein sequencing, i.e. the identified peptides, MASCOT score and coverage, are provided in raw data: xls supplement R3.

**Supplementary Figure 3:** Signal and noise discrimination within EMSA-PSeq samples (see text for details).

**Nucleic acid binding proteins attracted by probes derived from SNPs rs152730, rs152731 and rs152744 and identified as unique by EMSA-PSeq**

We have categorized the 40 unique proteins identified by EMSA-PSeq according to their primary interaction partners: i) as true transcription factors that bind DNA in a sequence-specific manner, and ii) as RNA-binding proteins or chromatin-interacting partners that might not directly interact with DNA but are part of a DNA-binding complex through protein-protein interactions (SupplTab 4). We have noticed that the proportion of RNA proteins differs between the P65 and the SCNN1B data sets. Only two out of 11 proteins (P65) but more than half (rs152730), all (rs152731) and most (rs152744) identified proteins were annotated as RNA-interaction partners. Hence, our SCNN1B dsDNA probes attracted primarily proteins known for their RNA-binding capacity. In contrast, we have not found any of the 8 transcription factors predicted to interact specifically with rs152730, rs152731, or rs152744 (SupplTab 3a) in our EMSA-PSeq data.

The P65 consensus probe has attracted P65, STAT3 and STAT6 which are known to interact directly. We thus mined our set of 40 proteins for interaction partners using the BioGrid database (<http://thebiogrid.org/>^10,11^). Among the 1666 interactions listed for the 11 proteins identified to interact specifically with the P65 consensus probe (Fig 4, SupplTab 4), only STAT3, STAT6 and P65 were listed as interaction partners. No interactions were reported in BioGrid between any of the 16 proteins identified for rs152730 (440 pairwise interactions listed in BioGrid), any of the five proteins identified for rs152731 (84 pairwise interactions listed in BioGrid) or any of the eight proteins identified for rs152744 (715 pairwise interactions listed in BioGrid). In contrast, BioGrid interaction partners of these unique 29 proteins such as hnRNPs and SRSFs were found by EMSA-PSeq. However, as these were often attracted to more than one SNP or expressed at levels of more than 200 ppm according to Pax-DB, these were not identified as unique interaction partners by EMSA-PSeq (Fig 4, SupplFig 3).

**Nucleic acid binding proteins identified by EMSA-PSeq on probes for more than one SNP**

The unique proteins attracted to EMSA-PSeq probes were enriched for RNA-binding proteins (SupplTab 4). Additionally, an alternative SCNN1B transcript generated by exon-read-through of SCNN1B exon 3 was observed (Fig 1C, SupplFig 5). Thus, we wanted to know how our EMSA-PSeq samples indicate occupancy of rs152730, rs152731 and rs152744 with splicing-associated RNA-binding proteins such as heterogeneous nuclear ribonucleoprotein (hnRNP) or the serine/arginine-rich splicing factor (SRSF) family. Moreover, we have noticed that 1 and 3 of the proteins identified as candidates for a specific interaction partner of the P65 consensus probe and the SCNN1B SNPs are annotated as components of the spliceosome (USP39, THOC1, ACIN1, PUF60; SupplTab 4 and Fig 4). Consequently, we have systematically compared the occupancy of our EMSA-PSeq samples with other known components of the spliceosome (accessed at <http://www.genome.jp/kegg-bin/show_pathway?map=ko03040&show_description=show> in 01/2016^12^).

As noted before, we are well aware that our PAA gel fragments, excised to identify interaction partners bound to our biotinylated probes, likely contain more than one multiprotein complex migrating similarly on a native PAA gel. Hence, we cannot exclude that we identify “innocent bystanders”, i.e. proteins which have formed multiprotein complexes in the absence of our probe. However, a sequence-specific interaction of an RNA-binding protein with our DNA probe was observed for PRPF39, identified on samples #151 and #157 (rs152744A), but not on samples #150 and #156 (rs152744G) (Fig 4). As samples #151/#150 were loaded adjacently on one gel, and samples #156/#157 were loaded adjacently on a different gel (SupplFig 5), a contamination of PRPF39 between sample #151 to #157 cannot explain this duplicate finding.

Contradictory to the suggestive allele-specific interaction of PRPF39 with rs152744A is the occurrence of hnRNP species within the EMSA-PSeq samples without any probe preference. Highly expressed hnRNPs were found in almost all samples and appeared even in negative control samples obtained after the electrotransfer of proteins. The global expression levels of hnRNPs were closely related to the number of positive samples (Fig 4), even though the nuclear extracts were derived from T84 cancer cells, expecting a partial atypical protein expression, e.g. for SRSF6 which is amplified in cancer^13^. While all of the hnRNPs were represented in at least one EMSA-PSeq sample, only a third of the components of the spliceosome annotated in the KEGG database were observed among the EMSA-PSeq samples (Fig 4). Of the 40 spliceosomal proteins, 11 were found on probes for more than one SNP: The highly expressed HSPA1A was observed on all samples but #241 and #243 (rs152731). LSM2 and SRSF1 were not found on samples #150, #151, #156 and #157 (rs152744) but found on at least one of the samples for rs152730, rs152731 and a control SNP from IFNGR1. EIF4A3, EFTUD2, CTNNBL1, NCBP1, RBM8A, SNRPD1, U2AF2 and SRSF2 were absent from samples #241 and #243 (rs152731) while all of these proteins were seen in at least one sample for each of the other three SNPs. We interpret these findings as a partial probe preference of the aforementioned proteins. Nevertheless, we cannot exclude that these observations, based on the absence of specific components from mere duplicate (rs152731) or quadruplicate (rs152744) samples result from randomly associating protein complexes which do not contain the DNA probes.

**In silico analysis of the influence of rs152730, rs152731, rs152745, rs152744, rs152741 and rs152740 pre-mRNA structure and on the interaction with RNA binding proteins**

We wanted to know whether the SNPs associated with CF intrapair discordance, all of which are within the non-coding sequence of SCNN1B, may have an impact on the interaction of the pre-mRNA with RNA binding proteins (RBPs). First, we have used RNAsnp^1,2^ to compare the predicted structures of both alleles at the six SNP sites (SupplTab 1,2). While the structures of both alleles are rather similar (albeit not identical) for SNPs rs152730, rs152731, rs152745 and rs152744, the C and U allele at rs152741 as well as the A and U allele at rs152740 are likely to realize different secondary structures in vivo (SupplTab 1,2).

Next, we have used RBPmap^14^ to see whether the contrasting alleles at these six SNPs can change a known consensus binding site of an RNA binding protein (SupplFig 1). Within a 200 bp interval surrounding the SNPs, no interaction partners were predicted near rs152745, rs152741 and rs152740. In contrast, near SCNN1B variants rs152730, rs152731 and rs152744, motifs for five, 15 and two RBPs were identified by RBPmap. None of these motifs for RNA interaction partners covered the SNP position (SupplFig 1). Of note, only probes derived from these three SNPs which were predicted to occur in the vicinity of RBP sites, have provided high-molecular-weight complexes sufficiently stable to withstand different buffer compositions during electrophoresis and to be recognized after brief, partial electrotransfer on a membrane (SupplFig 5).

Finally, we have used the catRAPIDomics web server to predict protein-RNA-interactions^15^. For a 50bp segment surrounding the six SNPs, interaction for 2636 proteins were valued by the catRAPIDomics server. catRAPIDomics scores did not exceed the threshold for significant strong binding at any of the twelve analyzed sequences for any of the 2636 proteins (31632 catRAPIDomics scores < 3). However, when we compared the interaction scores for both contrasting alleles at each of the SNP sites, scores obtained for the C and the U allele at rs152744 differed by 1.0 (all other 15815 score differences: -0.3 < DELTA catRAPIDomics score < 0.3) for SRSF9. We have interpreted this finding to represent a weak SRSF9/SCNN1B-pre-mRNA interaction which is modified by the nucleotide exchange at rs152744.

Based on the catRAPIDomics prediction of differential binding of SRSF9 to rs152744, we have reviewed our EMSA-PSeq data sets for the presence of SRSF9. While SRSF9 was not detected in any of our EMSA-PSeq samples, we could identify the following interaction partners of SRSF9 by EMSA-PSeq (SupplTab 4, Fig 4):

- likely non-specific, as they were found in all native EMSA-PSeq samples but not in SDS-Gel samples or negative controls: 15 out of 70 SRSF9-Biogrid interaction partners
- on rs152730: HNRNPUL2; SRSF9-HNRNPUL1 interaction described by Hegele et al.^16^
- on rs152744: FUS; FUS-SRSF9 interaction described by Wang et al.^17^
- on rs152730 and rs152744: ELAV1; ELAV1-SRSF9 interaction described by Abdelmohsen et al.^18^
- on rs152730: MAGOH; MAGOH-SRSF9 interaction described by Singh et al.^19^
- on rs152744: EEF1B2; EEF1B2-SRSF9 interaction described by Havuginama et al.^20^
- on rs152730: SRSF1, SRSF2, SRSF3, SRSF4 and SRSF6
- on rs152731: SRSF1 and SRSF6
- on rs152744: SRSF2, SRSF3, SRSF6

Unrelated to SCNN1B, FUS was also detected within sample #245 (P65 consensus sequence used as probe), and ELAV1 was also observed attached to IFNGR1-SNP1. In summary, while SRSF9 was not observed in our EMSA-PSeq data, its binding partners FUS, EEF1B2 and three SRSF proteins have been attracted to rs152744, enabling the recruitment of SRSF9 to a protein-DNA-complex in close vicinity to the weak SRSF9-binding site altered by SNP rs152744 on the SCNN1B pre-mRNA.

| **Supplementary Table 4:** EMSA-PSeq detected interaction partners of probe NFkappaB-P65, and SCNN1B SNPs rs152730, rs152731, rs152744 | | | | | | | | | |  |
| --- | --- | --- | --- | --- | --- | --- | --- | --- | --- | --- |
| A: Probe with NFkappaB-P65 consensus sequence | | | | | | | | | |  |
| Protein  *Sample #* |  | Expression level [ppm]^1^ | Length of reference protein sequence^2^ | Mass [kDa]^3^ | calc. pI^3^ | MASCOT- Score ^4^ | Coverage ^4,5^ | Ligand^6^ | Annotation^7^ | |
| CARHSP1  *#245* | Calcium-regulated heat stable protein 1 | 132 | 147 | 7.6-  15.9 | 8.2-10.8 | 73.9 | 24.43 | R | regulation of transcription, DNA-templated; regulation of mRNA stability  *CARHSP1 is required for effective tumor necrosis factor alpha mRNA stabilization and localizes to processing bodies and exosomes. Pfeiffer JR, et al. Mol Cell Biol, 2011* | |
| CHAF1B  *#245* | Chromatin assembly factor 1 subunit B | ⎯ | 559 | 61.5 | 7.5 | 49.2 | 4.83 | C | DNA replication-dependent nucleosome assembly; regulation of transcription, DNA-templated; chromatin assembly  *Chromatin assembly factor I (CAF-I) is required for the assembly of histone octamers onto newly-replicated DNA.* | |
| EYA3  *#245* | EYA transcriptional coactivator and phosphatase 3 | ⎯ | 573 | 57.8- 62.6 | 5.0- 5.2 | 60.5 | 5.31 | TF | double-strand break repair; regulation of transcription, DNA-templated; chromatin modification  *This gene encodes a member of the eyes absent (EYA) family of proteins. The encoded protein may act as a transcriptional activator and have a role during development.* | |
| NCOA7  *#245* | Nuclear receptor coactivator 7 | 1.08 | 942 | 92.4-  106.1 | 5.2-  5.6 | 65.8 | 3.14 | TF | regulation of transcription, DNA-templated  *ERAP140 (=NCOA7) may represent a distinct class of nuclear receptor coactivators. ERAP140 is recruited by estrogen-bound ER alpha to the promoter region of endogenous ER alpha target genes in a cyclic pattern similar to that of other coactivators.* | |
| PKN1  *#245* | Serine/threonine-protein kinase N1 | 2.83 | 942 | 66.0-104.6 | 6.2-  8.4 | 85.1 | 5.31 | C | chromatin binding; protein serine/threonine kinase activity  *Protein kinase C-related kinase targets nuclear localization signals in a subset of class IIa histone deacetylases.* | |
| PSIP1  *#245* | PC4 and SFRS1 (serine/arginine-rich splicing factor 1) -interacting protein | 74.1 | 530 | 60.1 | 9.1 | 72.7 | 4.34 | C | RNA polymerase II transcription coactivator activity; chromatin binding; activating transcription factor binding; supercoiled DNA binding  *A regulation of LEDGF (=PSIP1) interaction with chromatin by cellular partners of its PWWP domain could be involved in several processes linked to LEDGF tethering properties, such as lentiviral integration, DNA repair or transcriptional regulation*. | |
| RELA  *#245* | Transcription factor p65 | 76 | 551 | 42.8-  60.2 | 5.7-  8.4 | 78.3 | 9.28 | TF | transcription factor activity, sequence-specific DNA binding; RNA polymerase II regulatory region sequence-specific DNA binding; chromatin binding  *NF-kappa-B is a ubiquitous transcription factor involved in several biological processes.* | |
| STAT3  *#245* | Signal transducer and activator of transcription 3 | 174 | 770 | 76.1-  88.0 | 6.2-  7.1 | 44.9 | 4.76 | TF | transcription factor activity, sequence-specific DNA binding; ligand-activated sequence-specific DNA binding; transcription factor binding  *In response to cytokines and growth factors, STAT family members are phosphorylated by the receptor associated kinases, and then form homo- or heterodimers that translocate to the cell nucleus where they act as transcription activators.* | |
| STAT6  *#245* | Signal transducer and activator of transcription 6 | 93.5 | 847 | 81.7-  94.1 | 6.2 | 30.5 | 3.39 | TF | regulation of transcription, DNA-templated; signal transduction; transcription, DNA-templated  *see STAT3* | |
| USP39  *#245* | U4/U6.U5 tri-snRNP-associated protein | 28.6 | 565 | 53.5-  65.3 | 8.5-  9.3 | 80.8 | 5.19 | R | spliceosomal complex assembly; mRNA processing; RNA splicing  *A novel genetic screen for snRNP assembly factors in yeast identifies a conserved protein, Sad1p (=USP39), also required for pre-mRNA splicing.* | |
| YAP1  *#245* | Yorkie homolog | 19.2 | 504 | 48.2-  54.4 | 5.0-  5.2 | 229.8 | 32.67 | C | chromatin binding; transcription coactivator activity; transcription corepressor activity; transcription regulatory region DNA binding | |
| B: Probes for EMSA-PSeq representing rs152730 | | | | | | | | | |  |
|  | | | | | | | | | |  |
| Protein  *Sample #* |  | Expression level [ppm]^1^ | Length of reference protein sequence^2^ | Mass [kDa]^3^ | calc. pI^3^ | MASCOT- Score ^4^ | Coverage ^4,5^ | Ligand^6^ | Annotation^7^ | |
| BAZ2B  *#148* | Bromodomain adjacent to zinc finger domain protein 2B | ⎯ | 2168 | 220.6-  236.4 | 6.4-  7.0 | 32.4 | 0.25 | TF | regulation of transcription, DNA-templated | |
| CHD5  *#155* | Chromodomain-helicase-DNA-binding protein 5 | 2.11 | 1954 | 120.9-  223.0 | 5.8-  6.4 | 45.5 | 2.26 | C | ATP-dependent helicase activity; chromatin binding; regulation of transcription, DNA-templated; chromatin modification | |
| DIEXF  *#155* | Digestive organ expansion factor homolog | ⎯ | 756 | 87.0 | 5.9 | 128.5 | 3.7 | R | U3 snoRNA binding; poly(A) RNA binding  *Loss of function of def selectively up-regulates Delta113p53 expression to arrest expansion growth of digestive organs in zebrafish. Chen J, et al. Genes Dev, 2005* | |
| GTF2F2  *#154,#155* | General transcription factor IIF subunit 2 | 28.6 | 249 | 28.4 | 9.2 | 50.4 | 9.24 | R | regulation of transcription, DNA-templated; mRNA splicing, via spliceosome  *Direct interaction between the subunit RAP30 of transcription factor IIF (TFIIF) and RNA polymerase subunit 5, which contributes to the association between TFIIF and RNA polymerase II. Wei W, et al. J Biol Chem, 2001* | |
| HNRNPUL2  *#155* | Heterogeneous nuclear ribonucleoprotein U-like protein 2 | 116 | 747 | 85.1 | 4.9 | 61.8 | 4.82 | R | poly(A) RNA binding  *Regulation of DNA-end resection by hnRNPU-like proteins promotes DNA double-strand break signaling and repair. Polo SE, et al. Mol Cell, 2012* | |
| PCBP4  *#148* | Poly(rC)-binding protein 4 | 5.67 | 403 | 8.0-  37.1 | 5.4-  8.7 | 30.5 | 17.39 | R | RNA binding; DNA binding; poly(A) RNA binding  *The poly(C)-binding proteins: a multiplicity of functions and a search for mechanisms. Makeyev AV, et al. RNA, 2002* | |
| PRMT6  *#154,#155* | Protein arginine N-methyltransferase 6 | ⎯ | 375 | 32.5-  41.9 | 5.0-  5.4 | 95.1 | 7.53 | C | base-excision repair; chromatin organization; regulation of transcription, DNA-templated; chromatin modification  *PRMT6 is recruited by RUNX1 and mediates asymmetric histone H3 arginine-2 dimethylation (H3R2me2a) at megakaryocytic genes in progenitor cells.* | |
| PTBP3  *#148* | Polypyrimidine tract-binding protein 3 | 102 | 552 | 8.2-  60.4 | 8.0-  9.4 | 32.4 | 6.49 | R | mRNA processing; RNA splicing  *The protein encoded by this gene binds RNA and is a regulator of cell differentiation. The encoded protein preferentially binds to poly(G) and poly(U) sequences in vitro.* | |
| REPIN1  *#154* | Replication initiator 1 | 9.15 | 567 | 16.1-  69.9 | 10.0-  11.6 | 71.3 | 21.23 | TF | DNA binding; poly(A) RNA binding  A repressor complex, AP4 transcription factor and geminin, negatively regulates expression of target genes in nonneuronal cells. Kim MY, et al. Proc Natl Acad Sci U S A, 2006 | |
| RRP36  *#148* | Ribosomal RNA processing protein 36 homolog | ⎯ | 259 | 29.1-  29.8 | 10.0-  10.1 | 42.0 | 2.37 | R | maturation of SSU-rRNA from tricistronic rRNA transcript (SSU-rRNA, 5.8S rRNA, LSU-rRNA); rRNA processing | |
| TBL1X  *#148,#149* | F-box-like/WD repeat-containing protein TBL1X | 7.46 | 577 | 57.0-  62.5 | 6.1-  6.6 | 33.1 | 1.33 | TF | transcription corepressor activity; transcription factor binding; histone binding; transcription regulatory region DNA binding  *This encoded protein is found as a subunit in corepressor SMRT (silencing mediator for retinoid and thyroid receptors) complex along with histone deacetylase 3 protein.* | |
| TBL1Y  *#148,#149* | F-box-like/WD repeat-containing protein TBL1Y | 2.12 | 522 | 39.7-  56.7 | 5.6-  5.8 | 33.1 | 1.92 | TF | transcription, DNA-templated; histone deacetylation  *This gene is highly similar to TBL1X gene in nucleotide sequence and protein sequence.* | |
| THOC1  *#149* | THO complex subunit 1 | 6.52 | 657 | 43.3-  75.6 | 4.9-  5.7 | 29.6 | 2.12 | R | regulation of DNA recombination; mRNA processing; RNA splicing; replication fork processing; regulation of DNA-templated transcription, elongation  *Human hHpr1/p84/Thoc1 regulates transcriptional elongation and physically links RNA polymerase II and RNA processing factors. Li Y et al Mol Cell Biol. 2005* | |
| TLX2  *#149* | T-cell leukemia homeobox protein 2 | ⎯ | 284 | 30.2 | 11.1 | 31.9 | 2.82 | TF | regulation of transcription, DNA-templated; sequence-specific DNA binding  *Studies of the mouse ortholog have shown that the encoded protein is crucial for the development of the enteric nervous system; in humans, loss-of-function may play a role in tumorigenesis of gastrointestinal stromal tumors.* | |
| TSR1  *#148,#149* | Pre-rRNA-processing protein TSR1 homolog | 9.41 | 804 | 91.8 | 7.4 | 35.6 | 0.62 | R | poly(A) RNA binding | |
| UTP3  *#148* | Something about silencing protein 10 | ⎯ | 479 | 54.6 | 5.6 | 39.1 | 1.25 | R | maturation of SSU-rRNA from tricistronic rRNA transcript (SSU-rRNA, 5.8S rRNA, LSU-rRNA); chromatin modification | |

| C: Probes for EMSA-PSeq representing rs152731 | | | | | | | | | |  |
| --- | --- | --- | --- | --- | --- | --- | --- | --- | --- | --- |
|  | | | | | | | | | |  |
| Protein  *Sample #* |  | Expression level [ppm]^1^ | Length of reference protein sequence^2^ | Mass [kDa]^3^ | calc. pI^3^ | MASCOT- Score ^4^ | Coverage ^4,5^ | Ligand^6^ | Annotation^7^ | |
| ESRP2  *#241,#243* | Epithelial splicing regulatory protein 2 | 6.25 | 727 | 7.3-  78.4 | 6.7-  9.6 | 91.6 | 36.92 | R | mRNA processing; RNA splicing; regulation of RNA splicing  *An ESRP-regulated splicing programme is abrogated during the epithelial-mesenchymal transition. Warzecha CC, et al. EMBO J, 2010* | |
| GAR1  *#26* | H/ACA ribonucleoprotein complex subunit 1 | 25.4 | 217 | 22.3 | 10.9 | 46.1 | 3.69 | R | poly(A) RNA binding; rRNA processing  *The box H/ACA ribonucleoprotein complex: interplay of RNA and protein structures in post-transcriptional RNA modification.* | |
| RBMXL1^8^  *#26* | RNA binding motif protein, X-linked-like-1 | 80.2 | 390 | 4.0-  42.3 | 5.1-  10.0 | 37.5 | 35.14 | R | core promoter binding; chromatin binding; RNA binding; mRNA processing; RNA splicing  *This gene represents a retrogene of RNA binding motif protein, X-linked (RBMX), which is located on chromosome X.* | |
| RNASEH2A  *#241,#243* | Ribonuclease H2 subunit A | 21.2 | 299 | 33.4 | 5.2 | 95.9 | 12.71 | R | mismatch repair; RNA catabolic process; DNA replication, removal of RNA primer | |
| VSIG8  *#243* | V-set and immunoglobulin domain-containing protein 8 | 0.43 | 414 | 43.9 | 7.2 | 92.6 | 5.56 | R | poly(A) RNA binding | |

| D: Probes for EMSA-PSeq representing rs152744 | | | | | | | | | |  |
| --- | --- | --- | --- | --- | --- | --- | --- | --- | --- | --- |
|  | | | | | | | | | |  |
| Protein  *Sample #* |  | Expression level [ppm]^1^ | Length of reference protein sequence^2^ | Mass [kDa]^3^ | calc. pI^3^ | MASCOT- Score ^4^ | Coverage ^4,5^ | Ligand^6^ | Annotation^7^ | |
| ACIN1  *#150,#151* | Apoptotic chromatin condensation inducer in the nucleus | 10.8 | 1341 | 147.3-  151.8 | 6.4-  6.7 | 77.7 | 2.38 | R | mRNA processing; RNA splicing; apoptotic chromosome condensation; negative regulation of mRNA splicing, via spliceosome  *This protein has also been shown to be a component of a splicing-dependent multiprotein exon junction complex (EJC) that is deposited at splice junctions on mRNAs, as a consequence of pre-mRNA splicing.* | |
| DDB2  *#156, #151, #157* | DNA damage-binding protein 2 | 5.27 | 427 | 26.7-  47.8 | 9.2-  9.8 | 105.9 | 11.89 | C | nucleotide-excision repair, DNA damage removal; DNA repair; nucleotide-excision repair; pyrimidine dimer repair; response to UV; histone H2A monoubiquitination; protein autoubiquitination  *This protein is the smaller subunit of a heterodimeric protein complex that participates in nucleotide excision repair, and this complex mediates the ubiquitylation of histones H3 and H4, which facilitates the cellular response to DNA damage. This subunit appears to be required for DNA binding.* | |
| DDX19B  *#151* | ATP-dependent RNA helicase DDX19B | 57.1 | 479 | 53.9-  54.5 | 6.3-  7.7 | 56.5 | 6.89 | R | ATP-dependent RNA helicase activity; RNA binding  *DEAD box proteins, characterized by the conserved motif Asp-Glu-Ala-Asp (DEAD), are putative RNA helicases. They are implicated in a number of cellular processes involving alteration of RNA secondary structure such as translation initiation, nuclear and mitochondrial splicing, and ribosome and spliceosome assembly.* | |
| EXOSC9  *#157* | Exosome complex component RRP45 | 2.31 | 439 | 39.2-  50.8 | 5.1-  5.3 | 62.8 | 5.92 | R | nuclear-transcribed mRNA catabolic process; rRNA processing; nuclear mRNA surveillance  *This gene encodes a component of the human exosome, a exoribonuclease complex which processes and degrades RNA in the nucleus and cytoplasm. This component may play a role in mRNA degradation.* | |
| PARN  *#150, #156, #157* | Poly(A)-specific ribonuclease PARN | 3.05 | 639 | 23.8-  73.4 | 5.0-  6.4 | 133.0 | 10.55 | R | nuclear-transcribed mRNA catabolic process, nonsense-mediated decay | |
| PRPF39  *#151,#157* | Pre-mRNA-processing factor 39 | 2.62 | 669 | 78.4 | 5.4 | 57.1 | 3.44 | R | mRNA processing; RNA splicing | |
| PUF60  *#151* | Poly(U)-binding-splicing factor PUF60 | 145 | 559 | 54.0-  59.8 | 5.3-  5.4 | 39.6 | 4.09 | R | transcription, DNA-templated; regulation of transcription, DNA-templated; mRNA processing; apoptotic process; RNA splicing | |
| SUPT5H  *#157* | Transcription elongation factor SPT5 | 11.4 | 1087 | 120.4-  120.9 | 5.1 | 69.7 | 3.32 | R | regulation of transcription, DNA-templated; chromatin remodeling  *hSpt5 function in transcription regulation and mRNA capping is essential for a subset of cellular and viral genes and may not be required for global gene expression.* | |

^1^ derived from: Protein Abundance Across Organisms pax-db (available at pax-db.org^7^); values given are in ppm derived from dataset "colon integrated"; data for the following proteins are not reported in the dataset “colon integrated due to very low expression levels evident from the dataset “Whole organism, SC (PeptideAtlas,Aug,2011)”: CHAF1B – 0.92 ppm; EYA3 – 0.44 ppm; BAZ2B - 0.10 ppm; DIEXF – 0.34 ppm; PRMT6 - 0.05 ppm; RRP36 – 0.82 ppm; TLX2 < 0.01 ppm; UTP3 – 0.83

^2^ from <http://www.uniprot.org>

^3^ if primary data obtained by MassSpec is consistent with multiple isoforms of one protein, range of molecular weight and calcPI is given

^4^ when protein was detected in more than one sample, best score and coverage is reported.

^5^ in case of multiple isoforms detected for one protein group, maximum coverage obtained for the smallest isoform is reported. Primary data obtained from protein sequencing, i.e. the identified peptides, MASCOT score and coverage, are provided in raw data: xls supplement R3.

^6^ hierarchical classification of primary interaction partners: TF – direct interaction with DNA / motif recognition; R – interaction with RNA; C- interaction with other DNA-binding proteins such as chromatin

^7^ Annotation was derived from: Panther - Gene ontology terms metabolic function and biological process; additional text in italic from: NCBI Gene – summary, bibliography, GeneRIF

^8^ RBMX and RBMXL1 are identified as members of one protein group in this experiment, i.e. cannot be distinguished

**E. Cell culture**

T84 were grown in Dulbeccos Modified Eagle Medium (DMEM/F12; 21331-020, Thermo Fisher Scientific, Waltham, Massachusetts, USA) supplemented with 10% fetal calf serum (FCS) and 1% of 100 X penicillin-streptavidin-glutamine-solution (10378-016, Thermo Fisher Scientific, Waltham, Massachusetts, USA) in 100 x 20 mm plates (83.1802, Sarstedt, Nümbrecht, Germany). 16HBE14o-, CFBE41o-and CFTE29o- were kindly provided by D. Gruenert^21^. Respiratory epithelial cell lines were grown on Fibronectin (354008, Corning, Corning, New York, USA) and Collagen I (354231, Corning, Corning, New York, USA) coated plates (83.3900.002, Sarstedt, Nümbrecht, Germany) with Minimal Essential Medium (MEM; 12360-038, Thermo Fisher Scientific, Waltham, Massachusetts, USA) supplemented with 10% FCS and 1% of 100 X penicillin-streptavidin-solution (14150-122, Thermo Fisher Scientific, Waltham, Massachusetts, USA) and 1% of 200mM L-Glutamine-solution (258030-081, Thermo Fisher Scientific, Waltham, Massachusetts, USA).

Storage of epithelial cell line biosamples for RNA analysis: Confluent epithelial cells were washed 2 x with 10 ml phosphate buffered saline (PBS). After removal of PBS, the cell culture dish was placed in the gaseous phase of liquid N_2_. Frozen plates were stored at -80°C.

**F. Nucleic acid analysis**

The genotyping data set described here has been employed within two projects: “Sequential association studies on cystic fibrosis sibling pairs and EMSA-captured protein sequencing indicate that the epithelial splicing regulatory protein ESRP2 regulates transcript processing of the cystic fibrosis modifier gene SCNN1B” (this work) and “Inter- and intrapair – comparison of affected sib pairs consistently identify a regulatory element within SCNN1B as a modifier of cystic fibrosis disease severity” (unpublished data, 03/2019).

Buffer chemicals and the following molecular biology grade reagents were purchased from Roth, Karlsruhe, Germany: Acrylamide (3029.1); dNTPs for PCR (K051.1, K052.1, K053.1, K050.1). The following enzymes were purchased from NE Biolabs, Ipswich, Massachusetts, USA: DNase I (#M0303S); M-MuLV RT (#M0253L); AMV LongAmp TaqRT-PCR Kit (#E5300S); all restriction enzymes for PCR-RFLP typing.

RNA from cell lines was isolated using the QIAamp RNA Blood Mini Kit (52304, Qiagen, Hilden, Germany) and the RNase-free DNase Set (79254, Qiagen, Hilden, Germany). The culture dish containing -80°C stored biosamples of epithelial cell lines was coated with 3.5 ml (T84) or 1.4 ml (16HBE14o-) freshly prepared RLT-buffer. Adherent cells were scraped from plate surface with a blue pipette tip and dissolved by pipetting the cell suspension several times. 750µl of cell suspension were transferred to the Qia-Shredder-Column provided with the QIAamp RNA Blood Mini Kit and processed according to the manufacturer`s instructions for RNA isolation and DNA removal.

M-MuLV-based cDNA synthesis was primed with p(dt)15 (Roche, Basel, Switzerland). Ribonucleases were inhibited with RNAsin (N2511, Promega, Fitchburg, Wisconsin, United States). Synthesis with M-MuLV RT was done in a 40µl reaction mix using 8 µg of RNA, 36 nmol of each dNTP, 150 pmol of oligo-dT primer, 80 U RNAsin and 100 U of M-MuLV RT. AMV synthesis was carried out according to the manufacturer`s instructions using 3µg of RNA as a template per 20µl reaction volume.

Storage of nucleic acid biosamples: genomic DNA, isolated as described elsewhere (Stanke et al., 2011) was stored at 4°C in TE buffer. RNA was stored at -80°C. cDNA was kept in H_2_O at -20°C.

Unlabelled and biotinylated oligonucleotides for use as PCR primer were purchased from BioTeZ, Berlin, Germany. PCR was carried out in 96-well-PCR plates (651550, 651590, Kremsmünster, Austria) or TubeStrips (G006, G006-FC, Kisker-Biotech, Steinfurt, Germany) on a Thermocycler primus 96 advanced (Peqlab, Erlangen, Germany).

For amplification of DNA and cDNA, the following polymerases were used: Taq DNA Polymerase (3020302200, Stratec, Birkenfeld, Germany); Goldstar DNA Polymerase (ME-0064-05, Eurogentec, Seraing, Liège, Belgium); Long-Range PCR Kit (206403, Qiagen, Hilden, Germany). If required, betain was added to enhance amplification (14300, Sigma-Aldrich, St. Louis, Missouri, USA). Amplified DNA fragments were visualized after electrophoresis with LE Agarose (840004, Biozym, Hamburg, Germany) using GelRed Nucleic Acid Strain (41003 Biotium, Hayward, CA, purchased via BioTrend, Köln, Germany).

SCNN1B transcripts were amplified with Taq polymerase in 30µl reactions whereby the composition of the reaction mix were as described for PCR-RFLP except for the template of 1 µg cDNA, estimated under the assumption that all RNA provided for transcription was converted to cDNA. Amplification using the AMV LongAmp TaqRT-PCR Kit were done according to the manufacturer`s instructions providing 0.75 µg of cDNA as a template.

To map the previously observed association signals within the SCNN1B/SCNN1G genomic region^22^, 300 candidate SNPs were selected from dbSNP at http://www.ncbi.nlm.nih.gov/projects/SNP in 2008. 60 SNPs were selected from this pool based on predicted informativity (MAF > 0.4) and position on the genomic map, aiming for a spacing of 2 kb to 10 kb between adjacent SNPs. These 60 SNPs were pre-screened *in silico* for suitability in the SNPstream high-throughput genotyping apparatus and 48 were accepted for typing in a multiplex SNPstream assay after in silico analysis the DNA sequences. Of these 48 SNPs, no data was obtained for five loci. For further six SNPs typed by the SNPstream 48plex assay, inconsistencies such as non-mendelian inheritance were noticed in five to seven families and these markers were retyped by PCR-RFLP on the entire collection of more than 300 DNA samples. Furthermore, rs168748 was genotyped by PCR-RFLP to avoid an intramarker gap of more than 10 kb between adjacent informative SNPs.

Genotyping of SNPs by PCR-RFLP in a 30µl reaction volume was done using 50 ng of genomic DNA, 0.4 U of Taq DNA Polymerase, 15 pmol of each primer, 6 nmol of each dNTP and experimentally predetermined amounts of MgCl2 (range: 50 nmol to 150 nmol), dimethylsulfoxide (up to 1µl) and betain (up to 30 nmol). The restriction digest to distinguish both alleles at a SNP site by PCR-RFLP was done on 10 to 15 µl of unpurified PCR product using 1 U of restriction enzyme. Incubation was done overnight for classical restriction enzymes such as BsaXI, HpyCH4IV or HpaII, but restricted to 2 h for time-saver enzymes provided by NE Biolabs such as BslI and DdeII.

SNPstream genotyping was carried out with the GenomeLab SNPstream high-throughput 48-plex genotyping platform from Beckman Coulter following manufacturer's instructions. Primers for the multiplex PCR and single-base extension (SBE) were designed with web-based software provided at http://www.autoprimer.com (Beckman Coulter inc. Fullerton, CA). To ensure genotyping quality, several controls were included to exclude mix-ups and other errors during genotyping. Thus, each 384-well plate contained 4 blanks to detect contamination with DNA and 4 randomly selected replicates, which are expected to yield identical genotypes for a given genetic variant. 48-plex PCR reactions were performed in 384-well plates (4titude, UK) in a volume of 5 μl using 4 ng of DNA, dNTP mix (90 µM each) , 0.5 U of AmpliTaq Gold (Perkin-Elmer, Wellesley, MA), 1 X PCR buffer II, 5mM Mg and the 48 PCR primers at a concentration of 50 nM each. Thermal cycling was performed in GeneAmp PCR system 9700 thermal cyclers (Applied Biosystems, Foster City, CA) using the following program: initial denaturation at 94°C for 1 min followed by 40 cycles of 94°C for 30 s, 55°C for 30 s, 72 °C for 1 min and 4°C final hold temperature. Following PCR, plates were centrifuged briefly and 3 μl of a 1:25 dilution of SBE Clean-Up Reagent (USB; Cleveland, OH) was added to each well. The plates were sealed and incubated for 30 min at 37 °C and at 96 °C for 10 min. After the purification step, the SBE reaction was performed using reagents and protocols specific to the SNPstream platform. In the last step every extension product was hybridized to the complementary oligonucleotides arrayed on the 384-well microplates (SNPware 48-plex Tag array plate) by incubation at 42°C for 2 hours with humidity close to 100%. The SNPware plates were imaged after washing and drying with a two-laser, two-color charged couple device-based imager (GenomeLab SNPstream array imager). The 48 individual SNPs were identified by their position and fluorescent color in each well according to the position of the tagged oligonucleotides. Sample genotype data was generated on the basis of the relative fluorescent intensities for each SNP and electronically processed for graphical review.

Microsatellite markers were amplified in a 15 µl reaction volume using 50 ng of genomic DNA, 0.25 U of Taq DNA Polymerase, 12.5 pmol of unbiotinylated and 2.5 pmol of biotinylated primer, 3 nmol of each dNTP and experimentally predetermined amounts of MgCl2 (range: 25 nmol to 150 nmol), dimethylsulfoxide (up to 1 µl) and betain (up to 30 nmol). Biotinylated PCR products were separated and transferred onto a Hybond N+ positively charged nylon membrane (RPN303B, GE Healthcare Life Sciences, Little Chalfont, United Kingdom) by direct blotting electrophoresis (GATC, Konstanz, Germany). Signals were visualized using Blocking reagent (11096176001, Roche, Basel, Switzerland), Streptavidin-AP Conjugate (1189161001, Roche, Basel, Switzerland) and CDPstar (T2305, Applied Biosystems, Waltham, Massachusetts, USA).

Signals generated by GelRed were visualized on a GelDoc XR Molecular Imager (Bio-Rad, Hercules, California, USA). CDPstar signals from microsatellite genotyping and luminol signals generated for EMSA-PSeq experiments (see below) were visualized on a DNR-MF-ChemiBIS 3.2 Bio-Imaging System (Berthold Technologies, Bad Wildbad, Germany).

Long-rang PCR products derived from genomic DNA were sequenced at Qiagen (Hilden, Germany). PCR products derived from cDNA were sequenced at the Cologne Center of Genomics (Cologne, Germany).

**Supplementary Table 5a:** Primers for polymerase chain reaction (all sequences: 5`to 3`direction); genotyping

| Targeted candidate gene | Marker | Primer A | Primer B | Restriction enzyme* |
| --- | --- | --- | --- | --- |
| *GFI1* | microsatellite | AGGTGTGTGGACAGTGTGGA | Biotin-GGGTGAGGAGAGCAGCAG |  |
| *GATA2* | microsatellite | AACCACAGGGTTTGAACGAC | Biotin-CAGCAGAAGAAGTCAAAAATTAGGA |  |
| *PAX6* | microsatellite | TGGCCTGAAATAGCCAAATC | Biotin-GGGCCCTTTGCATAGAAGA |  |
| *HNF1* | microsatellite | CCTTGTCCAAGGTCACAGGA | Biotin-GCCAGTGATTAGGGTGCAAA |  |
| *FOS* | microsatellite | GCTCACAACATGCTTGACACT | Biotin-TGGATGATCTTGAGCAGACCT |  |
| *MAFF* | microsatellite | TGAGTGATGACAATTACACATGG | Biotin-CACCTCCTGAGGTCTCTTCA |  |
| *ESRP1* | microsatellite | CGGAAGGCAGAGCTTGTAG | Biotin-ACCTGCTTCAGCCTACCAAA |  |
| *ESRP2* | ESRP2Sat1 | CCTGTGACACGGAGGTTGTA | Biotin-GGGTCCTGGGGTCAGTATTT |  |
|  | ESRP2Sat2 | ACTGGGCAACAGGGTGAG | Biotin-TGGCCAGGATGGTTTCTATC |  |
|  | rs8059575 | AGCTACCGTGCACTCAGCTC | GGTCAGGAGTTGTGGGTTGTT | BslI |
|  | rs7184821 | CCTCTCCAGGCGAGTTACC | AGCGTGAGCTCTCCAGTCCTGGGT | XcmI |
|  | rs8057119 | AGCCCTTCTGGAACAGACAA | GGGACTATCTGCCTGCTGAG | HpyCHIV |
|  | rs11043 | AGGAGCACTGCACTCCCATA | CAGATGGGCTCAAATTGGGAGCTTG | BsaXI |
|  | rs13339471 | CTCCCCAAAAATAAGCCAGCT | TTCACCATGTTCAGGCTGGT | MwoI |
| *SCNN1B* | rs7204325 | CCAGCTGGGCTTGTTAAAGA | TGGACAAAGAAAGAAGGGAAAA | XmnI |
|  | rs2887481 | TTGTTATCCCTCTTTCTGTCCAC | CACTCGAGGGCTGTCAGTTT | TstI |
|  | rs152731 | ATCCAAAAGAGAGTCCTGACC | AGATCTCGGCTCACTGCAAC | DdeI |
|  | rs152745 | CCTGATGAGGGTATCTTGTG | CTTTCTGGCTGCAACTTGGT | MboI |
|  | rs152744 | CGGTTTTCTCATCCCTGAAG | CCAGCCTCATAGCCTTGTTC | BslI |
|  | rs152741 | CCAACATGGCGAAACCTT | ATGTGTGCCTGTGCATTTGT | HpaII |
|  | rs168748 | CATGACCCCATAACCCTGTC | GCTTTTTAGGTGCCCCTGTT | MspI |
|  | rs499812 | CAGGGTTGGACATGGTCATT | CTGTCTGCAGGCACCTGAT | MwoI |
|  | rs428438 | GCCCAGCCAATTTGCTATTA | AGTTTATCTTGCTCGAGCGGACTGCCT | TstI |
|  | rs4968006 | GGGAAATGATCAAATACATCCA | AAAAACAAGGTTGAGAATTCCTT | XmnI |

* if applicable, for PCR-RFLP SNP genotyping only

**Supplementary Table 5b:** Long-Range PCR for sequencing of *SCNN1B*

| Genomic fragment | Primer A | Primer B | Size of amplicon |
| --- | --- | --- | --- |
| rs152730-rs152740 | CTTCATAAACTTTTGGCCAGGT | GTAGGGAGTTGACAGCAGGTTC | 8856 bp |
| rs152730-rs152740 | GCAGGCCAGTGCCTATTTATCG | CCTAGCCTTATGGCAGGTGTGG | 8269 bp |

**Supplementary Table 5c:** Combinatorial PCR to detect and verify alternative transcript isoforms of *SCNN1B*

| Exon 3 – ESTs BM694355 and BU730506  (Amplicon 1 in Fig 1C; see also SupplFig 4C, lanes 5 and 6) | TCTCCATCTGGAACCACACA | GCTCTGGCTTCCATCTTGTC |
| --- | --- | --- |
| Exon 1 – ESTs BM694355 and BU730506 (Amplicon 2 in Fig 1C) | GTGTCCCAGTGTCACCAAC | GCTCTGGCTTCCATCTTGTC |
| ESTs BM694355 and BU730506 – Exon 4 (Amplicon 3 in Figure 1C, not observed) | GACAAGATGGAAGCCAGAGC | AGGGCTCAGCTCCGAATAG |
| Exon 3 – Exon 4 (Amplicon 4 in Fig 1C) | TCTCCATCTGGAACCACACA | AGGGCTCAGCTCCGAATAG |
| Exon 3 – Exon 5 (data not shown) | TCTCCATCTGGAACCACACA | GTCATGCCCCAGTTGAAGAT |
| Exon 1 – Exon 4 / (Amplicon 5 in Fig 1C; see also SupplFig 4C, lane 2) | GTGTCCCAGTGTCACCAAC | AGGGCTCAGCTCCGAATAG |
| Exon 1 – Exon 5 (data not shown) | GTGTCCCAGTGTCACCAAC | GTCATGCCCCAGTTGAAGAT |
| Exon 3 – ESTs AW844136 and CV337204 (not observed, see SupplFig 4B) | TCTCCATCTGGAACCACACA | ATCCGGCTTCTCATGACATC |
| Exon 3 – EST BX465038 (not observed, see SupplFig 4B) | TCTCCATCTGGAACCACACA | CAGTTCTTTAGCTTCCCTTGG |
| EST BX465038 – Exon 5 (not observed, see SupplFig 4B) | CGCATCACTCTATTGCCTCA | GTCATGCCCCAGTTGAAGAT |
| both intronic within EST BX465038 (not observed, see SupplFig 4B) | CCCCAAGGGAAGCTAAAGAA | GGAGGGAAGATGACGATCAC |
| ESTs BM694355 and BU730506 – Exon 5 (not observed) | GACAAGATGGAAGCCAGAGC | GTCATGCCCCAGTTGAAGAT |

**G. Electrophoretic mobility shift assay – Protein Sequencing (EMSA-PSeq)**

The following molecular biology grade reagents were purchased from Sigma-Aldrich, St. Louis, Missouri, USA: phenylmethanesulfonylfluoride (PMSF; P7626), dithiothreitol (DTT; D9779), spermidine (85578), spermine (85605), ATP (A7699), BSA (A7030).

Nuclear extracts were prepared from confluent T84 cells. The protocol for preparation of nuclear extracts was derived from *Current Protocols in Molecular Biology*^23^. All centrifugation steps were done at 4°C. Cell culture dishes were rinsed with PBS and cells were removed with trypsin. The cell suspension of 5 plates was collected in a 50 ml reaction tube and incubated with 20 ml of hypotonic buffer (10 mM HEPES, 2 mM MgCl2, 10 mM KCl, 0.2 mM PMSF, 1 mM DTT, pH 7.9) at room temperature. After centrifugation at 1850 x g / 15 min, the isolation of nuclear proteins was continued in a 4°C room. The supernatant was discarded and cells were resuspended in 5 x packed cell volume of hypotonic buffer. After centrifugation at 1850 x g / 5 min, supernatant was discarded and cells were resuspended in 3 x packed cell volume of hypotonic buffer. Suspension was transferred to 1.5 ml reaction tubes, left on ice for 10 min and homogenized 10 times with a micropistill. After centrifugation at 3300 x g /15 min, sedimented nuclei were resuspended in 1/2 packed nuclear volume of low salt buffer (20 mM HEPES, 2 mM MgCl2, 20 mM KCl, 25% glycerol, 0.2 mM EDTA, 0.2 mM PMSF, 1 mM DTT, pH 7.9) supplemented with 1/1200 packed nuclear volume of diluted OmniCleave endonuclease (10 U/µl; OC7810K, Epicentre, Madison, Wisconsin, USA). 1/2 packed nuclear volume of high salt buffer (20 mM HEPES, 2 mM MgCl2, 1.2 M KCl, 25% glycerol, 0.2 mM EDTA, 0.2 mM PMSF, 1 mM DTT, pH 7.9) was added dropwise, suspension was homogenized 4 times with a micropistill and incubated for 30 min at 400 rpm on a Thermomixer (Eppendorf, Hamburg, Germany). After centrifugation at 25000 x g / 30 min, supernatant was dialysed against 20 mM HEPES, 100 mM KCl, 20% glycerol, 0.2 mM EDTA, 0.2 mM PMSF, 1 mM DTT, pH 7.9 in a Slide-A-Lyzer (MWCO 2K, #66203, Thermo Fisher Scientific, Waltham, Massachusetts, USA) for 16-18 h. Completeness of dialysis was verified with a needle probe that measures the conductivity in volumes < 100 µl (customized apparatus, Forschungswerkstätten MHH, Hannover Medical School, Hannover, Germany) whereby the conductivity of the sample after dialysis and the conductivity of the HEPES-buffer used for dialysis was compared and observed to be equal. Dialysed nuclear extract was centrifuged at 25000 x g /20 min and the supernatant was stored at -80°C. Protein content of nuclear extract was assessed by Bradford assay and integrity of nuclear proteins was verified by SDS-electrophoresis and Coomassie stain.

High molecular weight DNA to serve as a non-specific competitor DNA during EMSA assays was prepared from chicken liver (samples #241, #243, #245) and common shrimp (samples #26,#27,#28, #146, #147, #148, #149, #150, #151, #152, #153, #154, #155, #156, #157) using phenol-chloroform extraction as described by Gross-Bellard *et al.*^24^. Tissue was minced with a scalpel, transferred to a 50 ml reaction tube with 50 ml lysis buffer (50 mM Tris-HCl, 109.5 g/l saccharose, 1% w/v Triton X-100), incubated for 30 min on ice and centrifuged at 600 x g / 15 min. Supernatant was discarded and sediment was washed twice with lysis buffer. Washed sediment was resuspended in 4 ml STE-buffer (50mM Tris-HCl pH 7.5, 100mM NaCl, 1mM Na_2_EDTA) with 0.5% SDS and 0.8 g/l Proteinase K (#03115836001, Roche, Basel, Switzerland) and incubated at 56°C in a shaking water bath for 16 h. Extraction of DNA was done with 3 ml of chloroform/isoamylalcohol (1:25) and 3 ml phenol (2 times) followed by 6 ml of chloroform/isoamylalcohol (1:25). DNA was precipitated from the aqueous phase on ice with sodium acetate and 99% chilled (-20°C) ethanol. After centrifugation 25000 x g / 4°C, 5 min, the pellet was washed with 70% chilled (-20°C) ethanol. Washed pellet was dissolved in TE (10mM Tris-HCl pH 8.0, 1mM Na_2_EDTA) and stored at 4°C.

Probes for the EMSA were purchased from BioTeZ, Berlin, Germany (SupplTab 8). Double-stranded oligonucleotide probes were prepared by incubating sense and double terminally biotinylated antisense strand for 5`at 75°C and next, the heater for 1,5 ml tubes was switched off and the annealed probes were allowed to cool to room temperature overnight.

To allow binding of DNA-interacting proteins with the probes representing the SCNN1B SNPs rs152730, rs152731, rs152745, rs152744, rs152741 and rs152740 under physiological conditions, the composition of the binding buffer (Pollock, 1997) was adjusted to mimic the milleu in the nucleus (SupplTab 7). The final composition of the buffer in which the reaction between probe and nuclear extract proteins took place was: 20 mM Tris-HCl pH 7.9, 125 mM KCl, 5 mM MgCl2, 10 mM NaCl, 0.1 mM ZnCl2, 1mM EDTA, 1mM DTT, 1mM Spermidine, 1mM Spermine, 2.5 mM ATP, 300 µg/ml BSA, 10% glycerol. In a final volume of 30 µl, 200 pmol of double-stranded DNA probe, 400 pmol of unspecific competitor polydIdC, 800 ng of high molecular weight genomic DNA from chicken or shrimp and 25 µg of nuclear extract proteins were left to form DNA-protein complexes. Agents were added sequentially at 37°C as follows: the annealed dsDNA probe was incubated with spermine, spermidine, ATP and BSA for 1 h; next, glycerol, competitor dIdC and high molecular weight DNA was added for 1 h and finally, the nuclear extract proteins were included in the reaction mixture for 2 h. 4 µl of loading buffer (8 g/l orange g, 0.6 g/l bromphenolblue, 0.6 g/l xylencyanol, 230 g/l Ficoll in 20 mM Tris-HCl, 125 mM KCl, 5 mM MgCl2, 10 mM NaCl, 0.1 mM ZnCl2, 1mM EDTA) were used to load the sample immediately on a native polyacrylamide gel.

Electrophoresis and blotting were done with a Mini-Protean Tetra Cell system (165-8001; Bio-Rad, Hercules, California, USA). Gels were prepared as 7% native polyacrylamide matrix (electrophoresis buffer and glycerol additive: see SupplTab 9). Electrophoresis was carried out at 100 V at 4°C. Biotin-labeled probes were blotted by electrotransfer in a denaturing, SDS-containing buffer onto a membrane assembly whereby the gel was covered with an uncharged nylon membrane (Hybond C, RPN303E, GE Healthcare Life Sciences, Little Chalfont, United Kingdom), followed by two charged nylon membranes (Hybond N+, RPN303B, GE Healthcare Life Sciences, Little Chalfont, United Kingdom). Signals were developed using an HRP-coupled antibody directed against biotin (21127, Thermo Fisher Scientific, Waltham, Massachusetts, USA) and luminol as a substrate on Hybond C membranes or Streptavidin-AP Conjugate (1189161001, Roche, Basel, Switzerland) and CDPstar (T2305, Applied Biosystems, Waltham, Massachusetts, USA) as a substrate on Hybond N+ membranes. Depending on the electrotransfer conditions (see SupplTab 9), free unbound probes were absent from the Hybond C membrane (see Fig 3, SupplFig 4) but visible on the first Hybond N+ membrane (see SupplFig 4).

Samples containing DNA-protein complexes were excised from either A. a gel blotted partially (samples #26, #27, #28, partial electrotransfer: 60min at 80V; samples #146, #147, #148, #149, #150, #151, #152, #153, #154, #155, #156, #157, partial electrotransfer: 10min at 20mA; see SupplTab 6, SupplFig 5) or from B. an unblotted gel (samples #241, #243, #245, electrotransfer: 90min at 50V; see SupplTab 9, Fig 3) whereby visualization of the signal was done from a neighboring lane loaded with a sample duplicate. Excised gel fragments were stored at -20°C until processing for Protein Mass Spectrometry. Polyacrylamide gels were cut into pieces of < 1mm and transferred into a 500 µl reagent tube.

Excised protein bands were fragmented into small pieces and destained two times with 200µl of 50% acetonitrile (ACN), 25mM ammonium bicarbonate at 37°C for 30min. Then, gel pieces were dried using 100% acetonitrile and a vacuum centrifuge (Eppendorf). Samples were incubated with 20µl of 1% SDS for 10min at 95°C to denature the proteins. Subsequently, they were washed two times with 25mM ammonium bicarbonate, dried again and incubated with 20µl 11mM dithiotreitol for 45min at 56°C. Then, denatured samples were redried, incubated with 20µl of 100mM iodoacetamide for 15min at room temperature in the dark, washed two times with 25mM ammonium bicarbonate, and dried again. Next, 20µl of 12 ng/µl sequencing grade trypsin (Fitchburg, Wisconsin, United States) in 10% ACN, 25mM ammonium bicarbonate were added. Samples were rehydrated in trypsin solution for 1 hour on ice and then covered with 10% ACN, 25 mM ammonium bicarbonate. Digestion was performed overnight at 37°C, then peptides were extracted by adding 50µl of 50% ACN, 0,1% triflouroacetic acid (TFA) at 37°C for 1 hour. This step was repeated twice, extracts were combined and dried in a vacuum centrifuge. Dried peptide extracts were redissolved in 30 µL 2% ACN, 0.1% TFA prior to LC-MS analysis.

LC-MS analysis was done as described previously (Jochim et al., 2011; Schroder et al., 2015). Briefly, sample aliquots were injected into a nano-flow ultra-high pressure liquid chromatography system (RSLC, Thermo Fisher Scientific, Waltham, Massachusetts, USA) equipped with a trapping column (5 µm C18 particle, 2 cm length, 75 µm ID, PepMap, Thermo Fisher Scientific) and a separating column (2 µm C18 particle, 50 cm length, 75 µm ID, PepMap, Thermo Fisher Scientific). Peptide mixtures were loaded to the trapping column at a flow rate of 6 µL/min with 0.1% TFA buffer for 5 min. After switching the trapping column online with the separation column and the nano-flow pumping system, peptides were eluted with a linear gradient of buffer B (80% ACN, 0.1% formic acid) in buffer A (0.1% formic acid) from 4% to 25% in 25 min, 25% to 50% in 10 min and 50% to 90% in 5 min. Flow rate was 250 nL/min and column temperature was set to 45°C. The outlet of the LC system was directly connected to the nano-ESI source (Thermo Fisher Scientific) of an LTQ Orbitrap Velos mass spectrometer. A voltage of 1.3 kV was applied at the ESI source. Overview scans were acquired at a resolution of 60k in a mass range of m/z 300-1600 in the orbitrap. The top 10 most intensive ions of charge two or three were selected for MS/MS analysis. Raw data were processed using Proteome Discoverer software (version 1.3, Thermo Fisher Scientific) and the Mascot search algorithm. Parameters were adjusted to a mass accuracy of <5 ppm for precursor masses and 0.7 Da for fragments; two missed cleavages were allowed and acetylation at the N-terminus, deamidation at glutamine and asparagines residues, and oxidation at methionine were included as dynamic modifications. Propionamidation at cystein residues was set as fixed modification. Mascot searches were done with the human entries of the Uniprot/Swissprot data base at a false discovery rate of 0.01.

**Enrichment analysis of EMSA-PSeq profiles**

We wanted to verify if our samples of excised high molecular weight DNA-protein-complexes are enriched for DNA-binding proteins derived from nuclear extract. In order to do this, the primary data provided by MASCOT was compiled in a single file and subjected to the Database for Annotation, Visualization and Integrated Discovery (accessible at david.abcc.ncifcrf.gov^25,26^). DAVID reported that our EMSA-PSeq samples are enriched in proteins involved in biological processes such as nucleosome organization (P = 6.0 x 10^-27^) and chromatin assembly (P = 3.2 x 10^-26^) (SupplTab 6). In other words, our EMSA-Pseq datasets were recognized to be enriched in nuclear proteins by DAVID.

**Supplementary Table 6:** Gene enrichment analysis of proteins identified by mass spectrometry in 25 EMSA-PSeq samples (raw output provided by DAVID, david.abcc.ncifcrf.gov^25,26^; accessed on 05.05.2015)


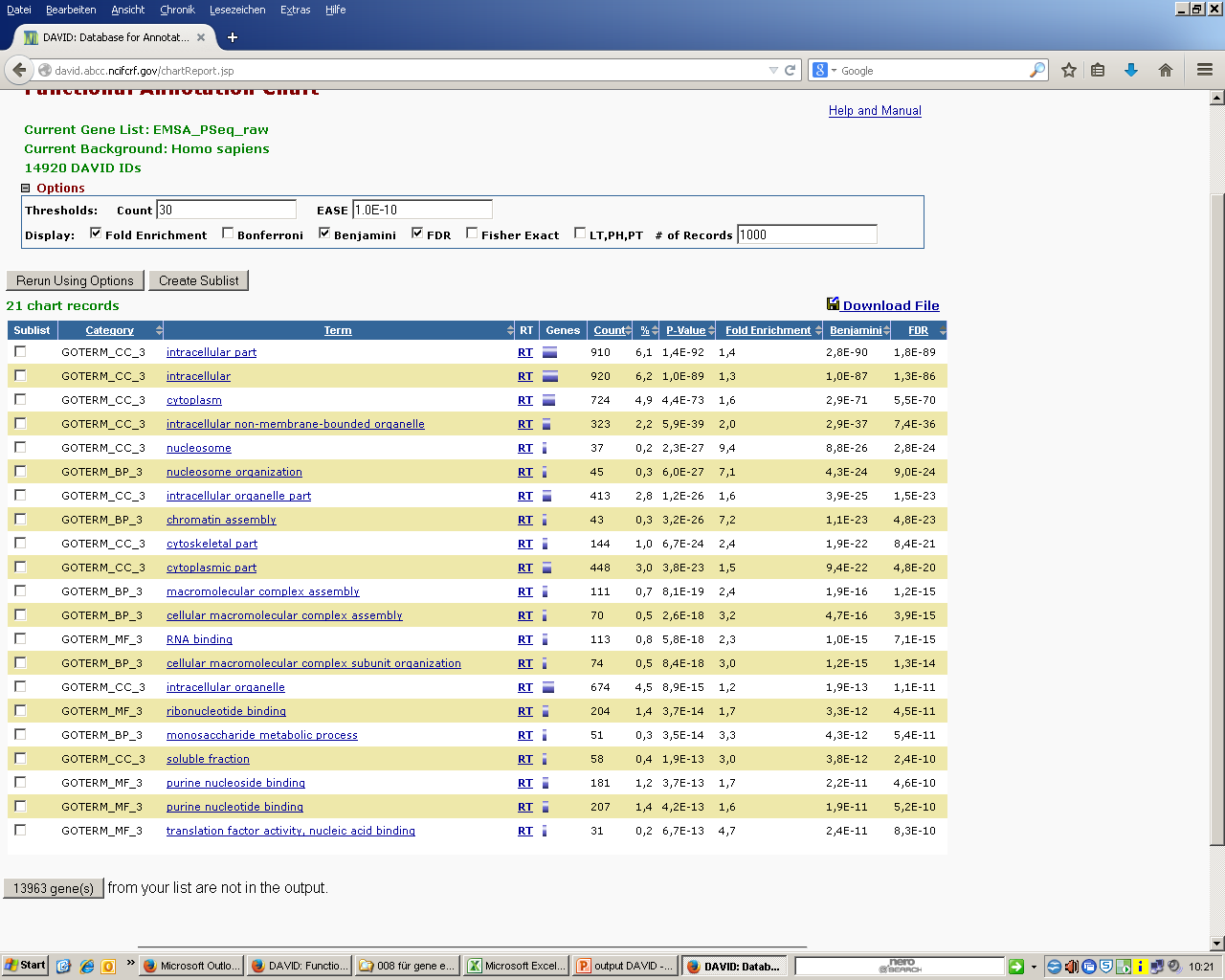


**Supplementary Table 7:** References for buffer composition, adjusted to mimic the nuclear milieu, of EMSA-PSeq binding reaction

| Ingredient | Reference |
| --- | --- |
| 20 mM Tris-HCl pH 7.9 | Pollock, 1997^27^ |
| 125 mM KCl | Century et al., 1970^28^ |
| 5 mM MgCl2 | Pollock, 1997^27^ |
| 10 mM NaCl | Century et al., 1970^28^ |
| 0.1 mM ZnCl2 | Andronikashvili et al., 1976^29^ |
| 1mM EDTA | Pollock, 1997^27^ |
| 1mM DTT | Pollock, 1997^27^ |
| 1mM Spermidine | Watanabe et al., 1991^30^ |
| 1mM Spermine | Feuerstein et al., 1990^31^;Schreck et al., 1990^32^;Watanabe et al., 1991^30^ |
| 2.5 mM ATP | Watanabe et al., 1991^30^ |
| 300 µg/ml BSA | Pollock, 1997^27^ |
| 10% glycerol | Pollock, 1997^27^ |

**Supplementary Table 8:** Probes for EMSA-PSeq (all sequences: 5`to 3`direction)

| Probe | ssDNA A | ssDNA B |
| --- | --- | --- |
| p65 consensus sequence* | AGTTGAGGGGACTTTCCCAGGC | Bio-GCCTGGGAAAGTCCCCTCAACT-Bio |
| rs152730-G | AACACAAATACAACAGTGAACAAAACAGAGTCCCT | Bio-AGGGACTCTGTTTTGTTCACTGTTGTATTTGTGTT-Bio |
| rs152730- T | AACACAAATACAACAGTTAACAAAACAGAGTCCCT | Bio-AGGGACTCTGTTTTGTTAACTGTTGTATTTGTGTT-Bio |
| rs152731-C | GGCTCACGCCTGTAATCCCAGCATTTTGAGAGGCC | Bio-GGCCTCTCAAAATGCTGGGATTACAGGCGTGAGCC-Bio |
| rs152731-T | GGCTCACGCCTGTAATCTCAGCATTTTGAGAGGCC | Bio-GGCCTCTCAAAATGCTGAGATTACAGGCGTGAGCC-Bio |
| rs152745-A | GATGAGGGGATCTTGTGATCAGCACACAGCTGTGT | Bio-ACACAGCTGTGTGCTGATCACAAGATCCCCTCATC-Bio |
| rs152745-G | GATGAGGGGATCTTGTGGTCAGCACACAGCTGTGT | Bio-ACACAGCTGTGTGCTGACCACAAGATCCCCTCATC-Bio |
| rs152744-A | TTCTCATCTCTGAAGCAAGAACAATAATCGTAGCT | Bio- AGCTACGATTATTGTTCTTGCTTCAGAGATGAGAA-Bio |
| rs152744-G | TTCTCATCTCTGAAGCAGGAACAATAATCGTAGCT | Bio- AGCTACGATTATTGTTCCTGCTTCAGAGATGAGAA-Bio |
| rs152741-C | AAAATACAAAAATTAACCGGGAGTGGTGGTGCACA | Bio- TGTGCACCACCACTCCCGGTTAATTTTTGTATTTT-Bio |
| rs152741-T | AAAATACAAAAATTAACTGGGAGTGGTGGTGCACA | Bio- TGTGCACCACCACTCCCAGTTAATTTTTGTATTTT |
| rs152740-A | GGATACCCTCTTCCTTGAGGGCCACCCCATGGCTA | Bio- TAGCCATGGGGTGGCCCTCAAGGAAGAGGGTATCC-Bio |
| rs152740-T | GGATACCCTCTTCCTTGTGGGCCACCCCATGGCTA | Bio- TAGCCATGGGGTGGCCCACAAGGAAGAGGGTATCC-Bio |
| polydIdC | ICICICICICICICICICICICICICICIC | ⎯ |

* derived from Schreck et al., 1990^32^

**Supplementary Table 9:** Properties of analyzed EMSA-PSeq samples

| Sample ^a^ | Probe | Allele | Estimated molecular weight of excised band^e^ | Gel | Separation buffer for EMSA gel | Blot prior to excision | Denaturation of sample prior to HPLC / MassSpec | MASCOT Analysis |
| --- | --- | --- | --- | --- | --- | --- | --- | --- |
| #102 | NEG CTRL. | ----- | approx. 10-25 kDa | native | Tris-Borate-EDTA | partial^f^ | no | non-stringent |
| #104 | NEG CTRL. | ----- | approx. 10-25 kDa | native | Tris-Borate-EDTA | partial^f^ | no | non-stringent |
| #56 | NEG CTRL. | ----- | approx. 10-25 kDa | native | Tris-Glycine-EDTA | partial^f^ | no | non-stringent |
| #57 | NEG CTRL. | ----- | approx. 10-25 kDa | native | Tris-Glycine-EDTA | partial^f^ | no | non-stringent |
| #240 ^c^ | NEG CTRL. | ----- | approx. 150-250 kDa | native | Tris-Borate-EDTA | yes | yes | validated |
| #242 ^d^ | NEG CTRL. | ----- | approx. 150-250 kDa | native | Tris-Borate-EDTA | yes | yes | validated |
| #244 ^b^ | NEG CTRL. | ----- | approx. 200-450 kDa | native | Tris-Borate-EDTA | yes | yes | validated |
| #245 ^b^ | NFkappaB-p65 | ----- | approx. 200-450 kDa | native | Tris-Borate-EDTA | no | yes | validated |
| #146 | IFNGR1-SNP1 | C | approx. 70-150 kDa | native | Tris-Borate-EDTA | partial^f^ | no | non-stringent |
| #152 | IFNGR1-SNP1 | C | approx. 70-150 kDa | native | Tris-Glycine-EDTA | partial^f^ | no | validated |
| #147 | IFNGR1-SNP1 | T | approx. 70-150 kDa | native | Tris-Borate-EDTA | partial^f^ | no | non-stringent |
| #153 | IFNGR1-SNP1 | T | approx. 70-150 kDa | native | Tris-Glycine-EDTA | partial^f^ | no | validated |
| #27 | IFNGR1-SNP2 | C | 60 kDa | denaturing | SDS/Tris/Glycine | partial^f^ | no | non-stringent |
| #28 | IFNGR1-SNP2 | T | 60 kDa | denaturing | SDS/Tris/Glycine | partial^f^ | no | validated |
| #148 | SCNN1B-rs152730 | G | approx. 70-150 kDa | native | Tris-Borate-EDTA | partial^f^ | no | non-stringent |
| #154 | SCNN1B-rs152730 | G | approx. 70-150 kDa | native | Tris-Glycine-EDTA | partial^f^ | no | validated |
| #149 | SCNN1B-rs152730 | T | approx. 70-150 kDa | native | Tris-Borate-EDTA | partial^f^ | no | non-stringent |
| #155 | SCNN1B-rs152730 | T | approx. 70-150 kDa | native | Tris-Glycine-EDTA | partial^f^ | no | validated |
| #241 ^c^ | SCNN1B-rs152731 | C | approx. 150-250 kDa | native | Tris-Borate-EDTA | no | yes | validated |
| #243 ^d^ | SCNN1B-rs152731 | T | approx. 150-250 kDa | native | Tris-Borate-EDTA | no | yes | validated |
| #26 | SCNN1B-rs152731 | T | 25 kDa | denaturing | SDS/Tris/Glycine | partial^f^ | no | non-stringent |
| #150 | SCNN1B-rs152744 | A | approx. 70-150 kDa | native | Tris-Borate-EDTA | partial^f^ | yes | validated |
| #156 | SCNN1B-rs152744 | A | approx. 70-150 kDa | native | Tris-Glycine-EDTA | partial^f^ | yes | validated |
| #151 | SCNN1B-rs152744 | G | approx. 70-150 kDa | native | Tris-Borate-EDTA | partial^f^ | yes | validated |
| #157 | SCNN1B-rs152744 | G | approx. 70-150 kDa | native | Tris-Glycine-EDTA | partial^f^ | yes | validated |

^a^ Please note that there are no exact technical duplicates among these samples as electrophoresis separation buffer, blotting conditions and preparation of samples for mass spectrometry varies (see Supplementary Methods for details).

^b^ Protein-DNA-complexes of sample #244, visualized after electroblotting, are displayed in the core manuscript (Fig 3). Sample #245 corresponds to the duplicate sample forwarded to protein mass spectrometry without protein loss by blotting.

^c^ Protein-DNA-complexes of sample #240, visualized after electroblotting, are displayed in the core manuscript (Fig 3). Sample #241 corresponds to the duplicate sample forwarded to protein mass spectrometry without protein loss by blotting.

^d^ Protein-DNA-complexes of sample #242 is displayed in within the core manuscript (Fig 3). Sample #243 corresponds to the duplicate sample forwarded to protein mass spectrometry without protein loss by blotting.

^e^ Size of high molecular weight complexes on native polyacrylamide gels were estimated using catalase (240 KDa; pI 5.5-6.0) and ferritin (450 kDa; pI 4.1-5.1), both purchased from Serva, Heidelberg, Germany (39064.01), as a size marker (SupplFig 6).

^f^ see SupplFig 4 and 5 for experimental conditions and samples provided after partial electrotransfer

**Supplementary Figure 4:** Optimization of conditions for partial electrotransfer

The aim of partial electrotransfer was the extraction of biotinylated probes from the high-molecular-weight DNA-protein complexes by short time and low current under denaturing conditions with the intention to extract the probe but leave most of the proteins within the gel for analysis by protein mass spectrometry. Free probes mostly passed through the Hybond C and were visualized on the subsequent Hybond N+. Please note that the electrophoresis conditions influence stability and thus detection of DNA-protein complexes as reported to be characteristic of this method^27^: an unfocussed high-molecular weight signal using the P65-consensus probe was seen only for the TBE-separated sample, but not for the TGE-separated sample (both: electrotransfer for 30min at 90 mA). Nuclear extracts were derived from T84 cells (T) or 16HBE14o- cells (H).

**Supplementary Figure 5:** Samples #146 - #157 provided for EMSA-PSeq after partial electrotransfer

**Supplementary Figure 6:** Separation of multimeric proteins with known size on a native 7% polyacrylamide / TBE gel

Under electrophoresis conditions used in the EMSA-PSeq experiments, proteins with pI in the alkaline range (cytochrome C, chymotrypsinogen A, aldolase) cannot enter the gel. The multimeric proteins catalase and ferritin both enter the polyacrylamide matrix, demonstrating that high molecular weight multiprotein complexes of 240 to 450 kDa can be observed in an EMSA-PSeq. A cautious estimate of molecular weight for complexes observed on the EMSA-PSeq probes is: NFkappaB-P65 consensus – HMW complex just barely enters gel (Fig 3) – 200-450 kDa; rs152731 – less than NFkappaB-P65 consensus HMW complex – 150-250 kDa (Fig 3); rs152730 – less than rs152731 HMW complex – 70-150 kDa (SupplFig 5); rs152744 comparable to rs152731 HMW complex – 70-150 kDa (SupplFig 5). The native 7% polyacrylamide gel was prepared from a 30% Acrylamide solution (37.5 monoacrylamide : 1 bisacrylamide; Rotiphorese30, Roth, Karlsruhe) in 1X Tris-Borate-EDTA buffer and 2.5% w/v glycerol. 10 X TBE: 108 g Tris base, 54 g boric acid, 7.44 g EDTA.

**Supplementary references**

1. Sabarinathan, R. *et al.* The RNAsnp web server: predicting SNP effects on local RNA secondary structure. *Nucleic Acids Res.* **41**, W475-479 (2013).

2. Sabarinathan, R. *et al.* RNAsnp: efficient detection of local RNA secondary structure changes induced by SNPs. *Hum. Mutat.* **34**, 546–556 (2013).

3. Cunningham, F. *et al.* Ensembl 2015. *Nucleic Acids Res.* **43**, D662-9 (2015).

4. Stanke, F. *et al.* Genes that determine immunology and inflammation modify the basic defect of impaired ion conductance in cystic fibrosis epithelia. *J. Med. Genet.* **48**, 24–31 (2011).

5. Mekus, F., Laabs, U., Veeze, H. & Tummler, B. Genes in the vicinity of CFTR modulate the cystic fibrosis phenotype in highly concordant or discordant F508del homozygous sib pairs. *Hum. Genet.* **112**, 1–11 (2003).

6. Labenski, H., Hedtfeld, S., Becker, T., Tummler, B. & Stanke, F. Initial interrogation, confirmation and fine mapping of modifying genes: STAT3, IL1B and IFNGR1 determine cystic fibrosis disease manifestation. *Eur. J. Hum. Genet. EJHG* **19**, 1281–1288 (2011).

7. Wang, M., Herrmann, C. J., Simonovic, M., Szklarczyk, D. & von Mering, C. Version 4.0 of PaxDb: Protein abundance data, integrated across model organisms, tissues, and cell-lines. *Proteomics* **15**, 3163–3168 (2015).

8. Mi, H., Muruganujan, A., Casagrande, J. T. & Thomas, P. D. Large-scale gene function analysis with the PANTHER classification system. *Nat. Protoc.* **8**, 1551–1566 (2013).

9. Mi, H., Poudel, S., Muruganujan, A., Casagrande, J. T. & Thomas, P. D. PANTHER version 10: expanded protein families and functions, and analysis tools. *Nucleic Acids Res.* **44**, D336-42 (2016).

10. Chatr-Aryamontri, A. *et al.* The BioGRID interaction database: 2015 update. *Nucleic Acids Res.* **43**, D470-478 (2015).

11. Stark, C. *et al.* BioGRID: a general repository for interaction datasets. *Nucleic Acids Res.* **34**, D535-539 (2006).

12. Kanehisa, M., Sato, Y., Kawashima, M., Furumichi, M. & Tanabe, M. KEGG as a reference resource for gene and protein annotation. *Nucleic Acids Res.* **44**, D457-462 (2016).

13. Cohen-Eliav, M. *et al.* The splicing factor SRSF6 is amplified and is an oncoprotein in lung and colon cancers. *J. Pathol.* **229**, 630–639 (2013).

14. Paz, I., Kosti, I., Ares, M., Jr, Cline, M. & Mandel-Gutfreund, Y. RBPmap: a web server for mapping binding sites of RNA-binding proteins. *Nucleic Acids Res.* **42**, W361-7 (2014).

15. Agostini, F. *et al.* catRAPID omics: a web server for large-scale prediction of protein-RNA interactions. *Bioinforma. Oxf. Engl.* **29**, 2928–2930 (2013).

16. Hegele, A. *et al.* Dynamic protein-protein interaction wiring of the human spliceosome. *Mol. Cell* **45**, 567–580 (2012).

17. Wang, J. *et al.* Toward an understanding of the protein interaction network of the human liver. *Mol. Syst. Biol.* **7**, 536 (2011).

18. Abdelmohsen, K. *et al.* Ubiquitin-mediated proteolysis of HuR by heat shock. *EMBO J.* **28**, 1271–1282 (2009).

19. Singh, G. *et al.* The cellular EJC interactome reveals higher-order mRNP structure and an EJC-SR protein nexus. *Cell* **151**, 750–764 (2012).

20. Havugimana, P. C. *et al.* A census of human soluble protein complexes. *Cell* **150**, 1068–1081 (2012).

21. Illek, B. *et al.* Cl transport in complemented CF bronchial epithelial cells correlates with CFTR mRNA expression levels. *Cell. Physiol. Biochem. Int. J. Exp. Cell. Physiol. Biochem. Pharmacol.* **22**, 57–68 (2008).

22. Stanke, F. *et al.* The TNFalpha receptor TNFRSF1A and genes encoding the amiloride-sensitive sodium channel ENaC as modulators in cystic fibrosis. *Hum. Genet.* **119**, 331–343 (2006).

23. Pollock, R. M. Determination of Protein-DNA Sequence Specificity by PCR-Assisted Binding-Site Selection. *Curr. Protoc. Mol. Biol.* **33**, 12.11.1-12.11.11 (1996).

24. Gross-Bellard, M., Oudet, P. & Chambon, P. Isolation of high-molecular-weight DNA from mammalian cells. *Eur. J. Biochem. FEBS* **36**, 32–38 (1973).

25. Huang, D. W., Sherman, B. T. & Lempicki, R. A. Systematic and integrative analysis of large gene lists using DAVID bioinformatics resources. *Nat. Protoc.* **4**, 44–57 (2009).

26. Huang, D. W., Sherman, B. T. & Lempicki, R. A. Bioinformatics enrichment tools: Paths toward the comprehensive functional analysis of large gene lists. *Nucleic Acids Res.* **37**, 1–13 (2009).

27. Pollock, R. M. DNA-Protein Interactions. in *Current Protocols in Molecular Biology* 12.0.1-12.11.11 (John Wiley and Sons, Inc., 1997).

28. Century, T. J., Fenichel, I. R. & Horowitz, S. B. The concentrations of water, sodium and potassium in the nucleus and cytoplasm of amphibian oocytes. *J. Cell Sci.* **7**, 5–13 (1970).

29. Andronikashvili, E. L., Mosulishvili, L. M., Belokobil’skiy, A. I., Kharabadze, N. E. & Shonia, N. I. Human leukaemic cells. Determination of trace elements in nucleic acids and histones by neutron-activation analyses. *Biochem. J.* **157**, 529–533 (1976).

30. Watanabe, S., Kusama-Eguchi, K., Kobayashi, H. & Igarashi, K. Estimation of polyamine binding to macromolecules and ATP in bovine lymphocytes and rat liver. *J. Biol. Chem.* **266**, 20803–20809 (1991).

31. Feuerstein, B. G., Pattabiraman, N. & Marton, L. J. Molecular mechanics of the interactions of spermine with DNA: DNA bending as a result of ligand binding. *Nucleic Acids Res.* **18**, 1271–1282 (1990).

32. Schreck, R., Zorbas, H., Winnacker, E. L. & Baeuerle, P. A. The NF-kappa B transcription factor induces DNA bending which is modulated by its. *Nucleic Acids Res.* **18**, 6497–6502 (1990).
