# Supplementary material for: Genetic information from discordant sibling pairs points to ESRP2 as a candidate trans-acting regulator of the CF modifier gene SCNN1B
Source: Sci Rep. 2020 Dec 31;10:22447. doi: 10.1038/s41598-020-79804-y (PMC7775467; doi:10.1038/s41598-020-79804-y)
Supplement: Supplementary file 2 — Supplementary information 2. [file 41598_2020_79804_MOESM2_ESM.pdf]

Supporting information for:

Genetic information from discordant sibling pairs points to ESRP2 as a candidate trans-acting regulator of the CF modifier gene SCNN1B

Tim Becker, Andreas Pich, Stephanie Tamm, Silke Hedtfeld, Mohammed Ibrahim, Janine Altmüller, Nina Dalibor, Mohammad Toliat, Sabina Janciauskiene, Burkhard Tümmler, Frauke Stanke

----- Source data for Figure 6 -----

Source data EMSA / Figure 6A,B

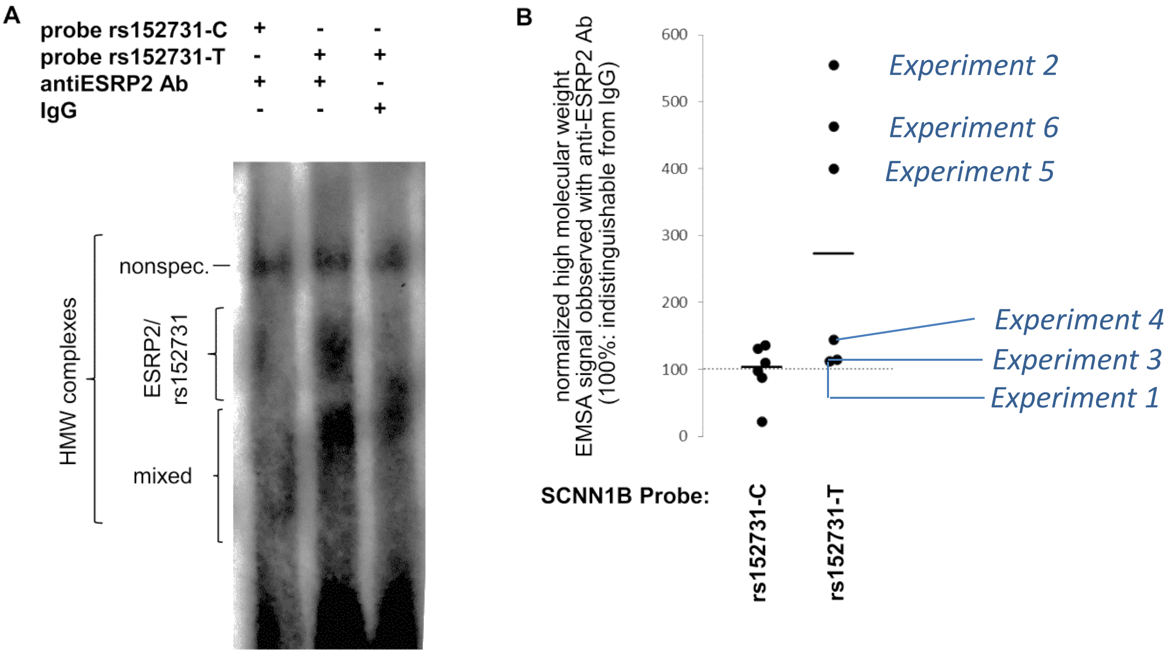

Raw data 6A is from: Experiment 6.

Nuclear extracts used in experiment have been freshly prepared for each experiment.

Dokumentation of primary data (GLP rules, Hannover medical school):
Experiment 1: Lab Book 6296, pages 46-49
Experiment 2: Lab Book 6296, pages 51-54
Experiment 3: Lab Book 6296, page 61
Experiment 4: Lab Book 6296, page 65
Experiment 5: Lab Book 6292, page 202 -205
Experiment 6: Lab Book 6292, page 206 - 210

Source data EMSA / Figure 6A,B *– technical variables*

Technical variables between experiments:

A. Source of nuclear extracts
Experiment 1: 16HBE14o-, derived from frozen cells
Experiment 2: 16HBE14o-, derived from frozen cells
Experiment 3: T84, derived from frozen cells
Experiment 4: 16HBE14o-, derived from frozen cells
Experiment 5: 16HBE14o-, derived from freshly cultured cells
Experiment 6: 16HBE14o-, derived from freshly cultured cells

B. Electrophoresis conditions
Experiment 1: 6% polyacrylamide; 250 V, 2,5 h
Experiment 2: 5% polyacrylamide; 40 V, 15 h
Experiment 3: 5% polyacrylamide; 250 V, 2,5 h
Experiment 4: 5% polyacrylamide; 300 V, 2 h
Experiment 5: 5% polyacrylamide; 16 h 40 V + 1,5 h , 100 V
Experiment 6: 5% polyacrylamide; 16 h 40 V + 1,5 h, 100 V

Influence of electrophoresis conditions on detection of ESRP2 / rs152731-T complexes by EMSA:

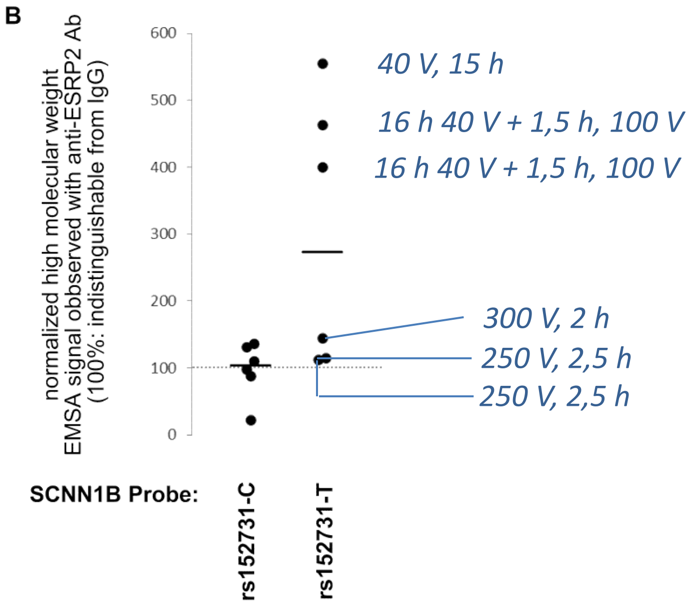

Source data EMSA / Figure 6A,B *– evaluation of lane profiles*

A. intensity distribution in the lane with anti-ESRP2-Ab is similar to the intensity distribution in the corresponding lane with IgG

a. profile shows one broad signal with one intensity maximum

- *the same high molecular weight area beween lanes is compared*
- *the position of the band is defined based on the signal observed with anti-ESRP2-Ab and the same area is evaluated for the corresponding IgG lane*
- ***Interpretation: specific and unspecific complexes have not been separated by electrophoresis***
- Experiments 1, 3

b. profile shows more than one intensity maximum

- *position of subbands is defined based on the signals observed with anti-ESRP2-Ab and the same areas are evaluated for the corresponding IgG lane*
- ***Interpretation: specific and unspecific complexes have been partially separated by electrophoresis***
- Experiments 2, 4

B. intensity distribution in lane with anti-ESRP2-Ab shows a signal that is absent from the corresponding IgG lane

- *position of the band is defined for the lane with the anti-ESRP2-Ab and the same area is evaluated for the corresponding IgG lane*
- ***Interpretation: specific and unspecific complexes have been separated by electrophoresis***
- Experiments 5,6

Source data EMSA / *Experiment 1 – uncurated data*

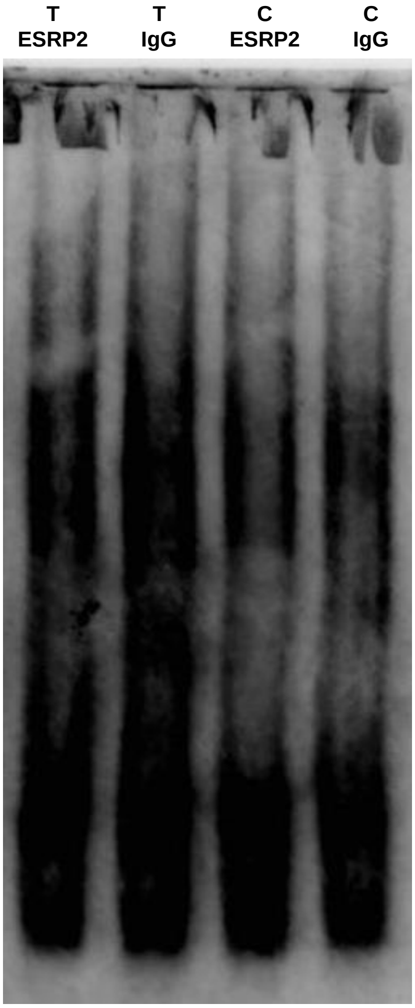

raw data

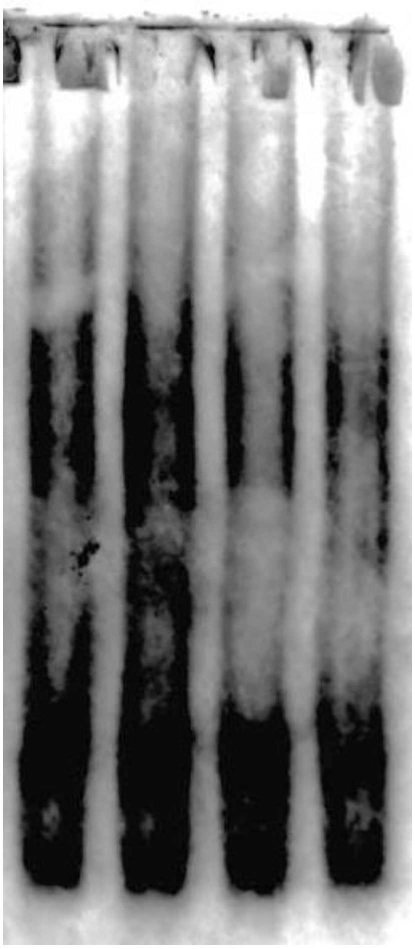

brightness and contrast adjusted for visualisation on screen

Legend:
EMSA Probe (rs152731 allele C or rs152731 allele T)
Ab (ESRP2 = anti-ESRP2-Ab; IgG = isotype control)

Source data EMSA / *Experiment 1 – lane profiles*

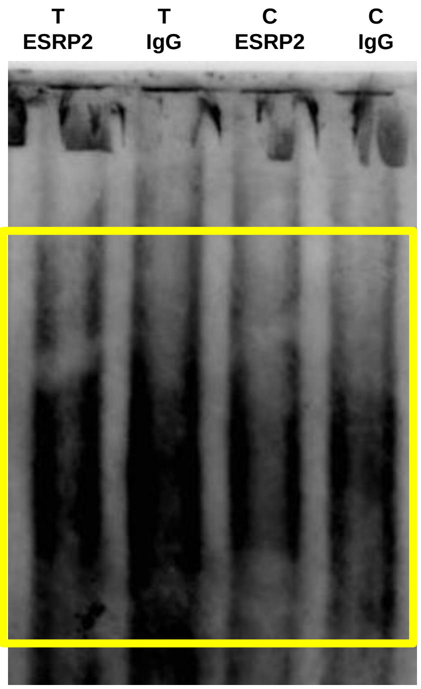

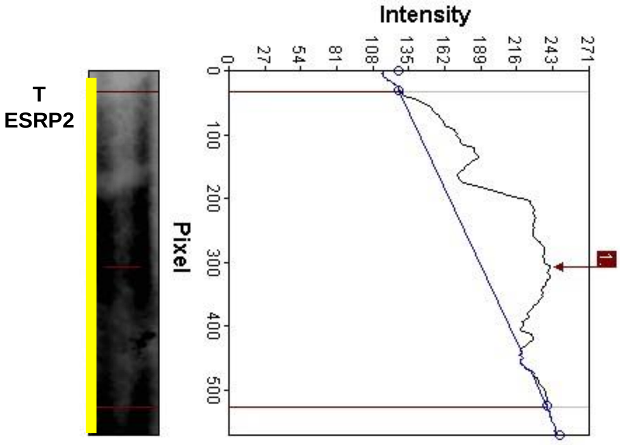

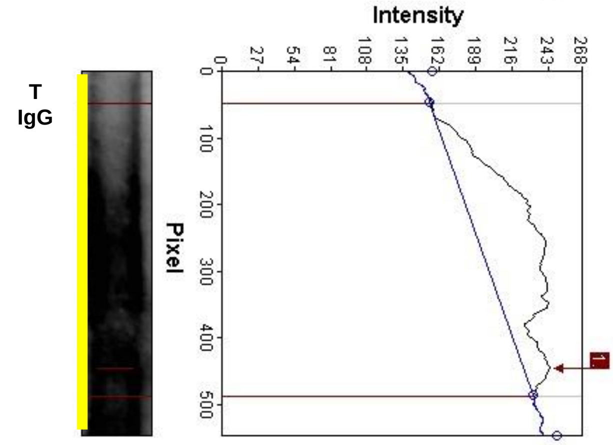

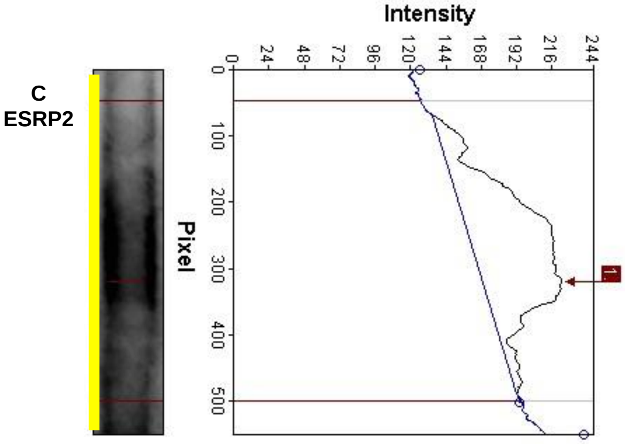

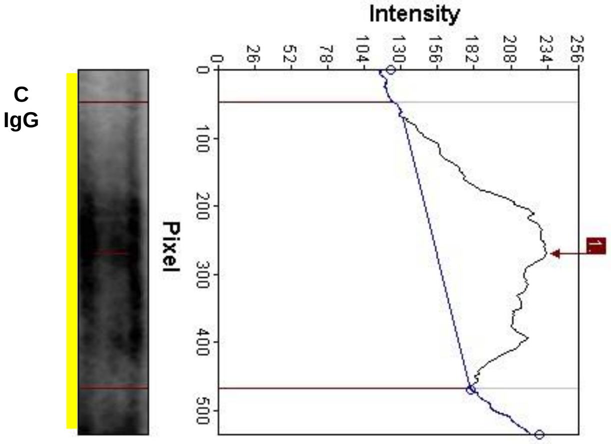

Source data EMSA / *Experiment 1 –densitometry*

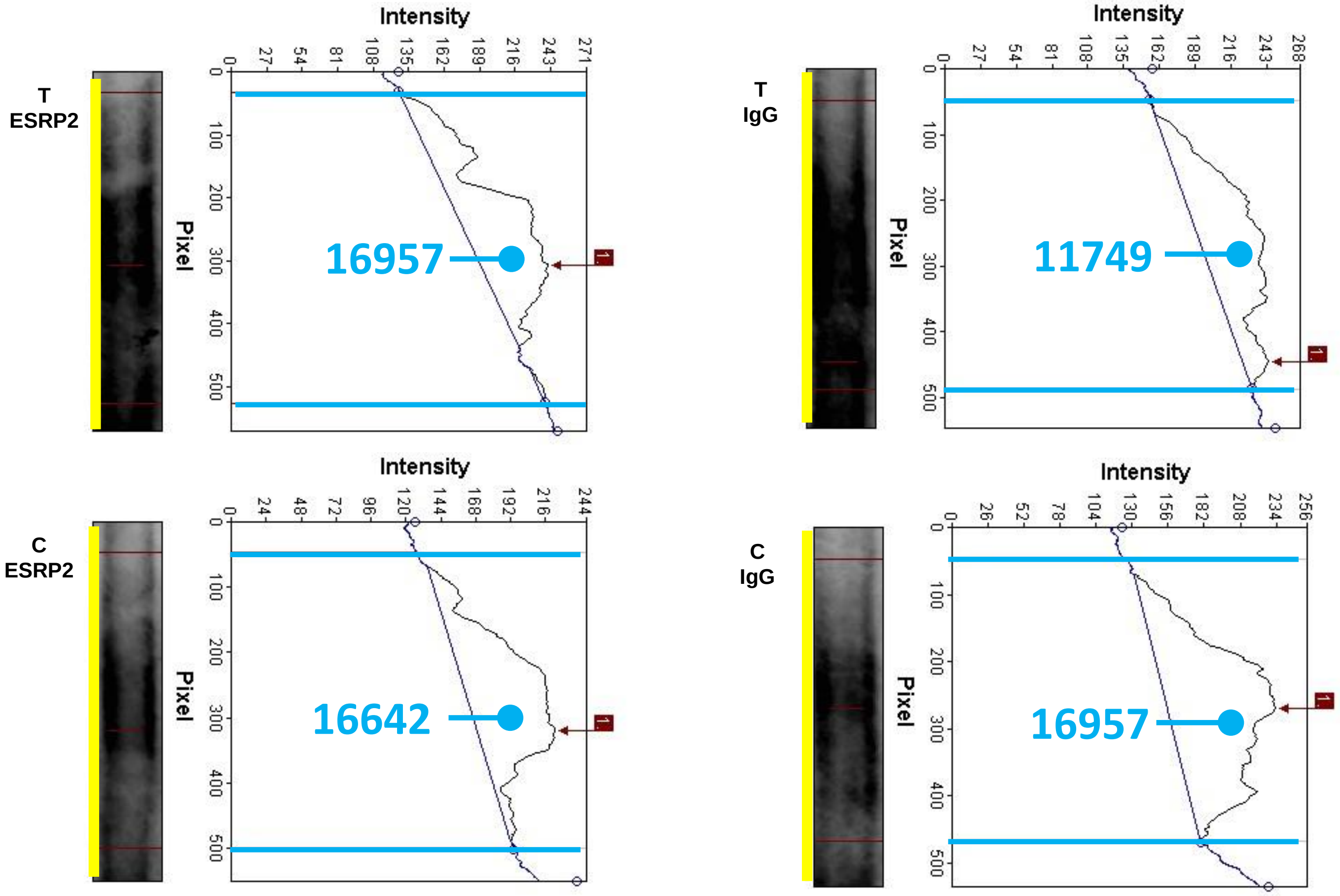

Raw volume was used to estimate ESRP2 bound on EMSA probes rs152731-T and rs152731-C. Signal intensity of the corresponding IgG lane was normalized to 100 %:

	anti-ESRP2-Ab	IgG
**rs152731-T**	144%	100%
**rs152731-C**	98%	100%

Source data EMSA / *Experiment 2 – uncurated data*

C IgG	C ESRP2	T IgG	T ESRP2

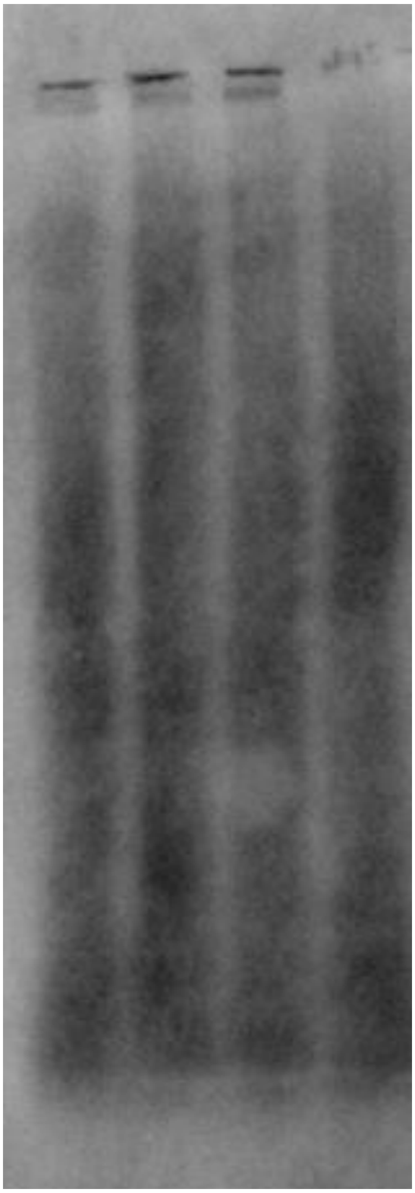

raw data

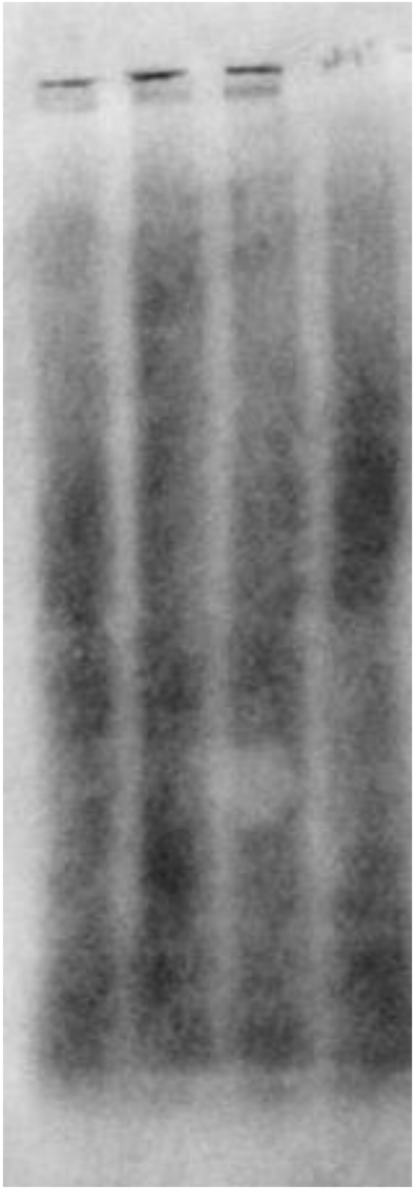

brightness and contrast adjusted for visualisation on screen

Legend:
EMSA Probe (rs152731 allele C or rs152731 allele T)
Ab (ESRP2 = anti-ESRP2-Ab; IgG = isotype control)

Source data EMSA / *Experiment 2 – lane profiles*

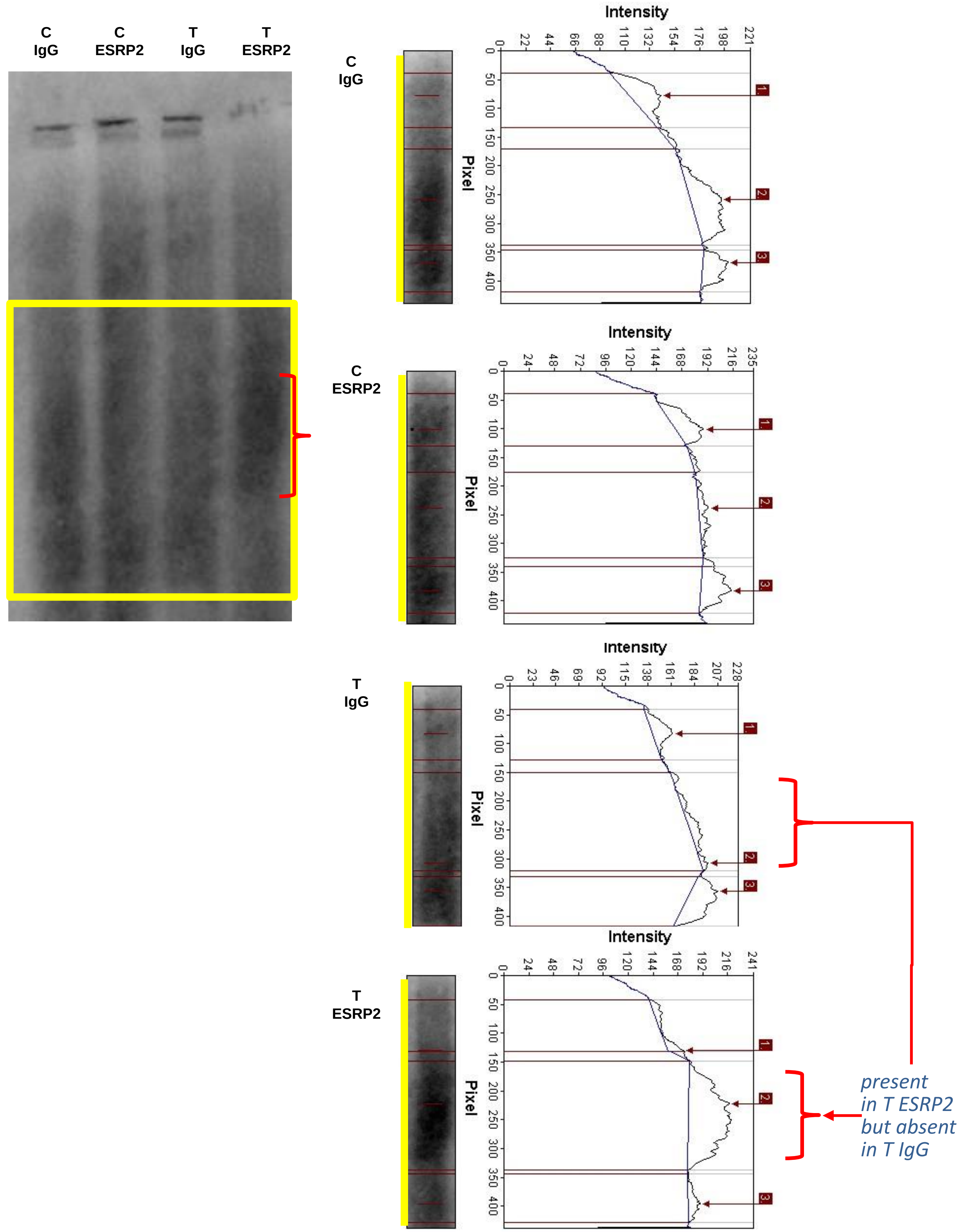

Source data EMSA / *Experiment 2 – densitometry*

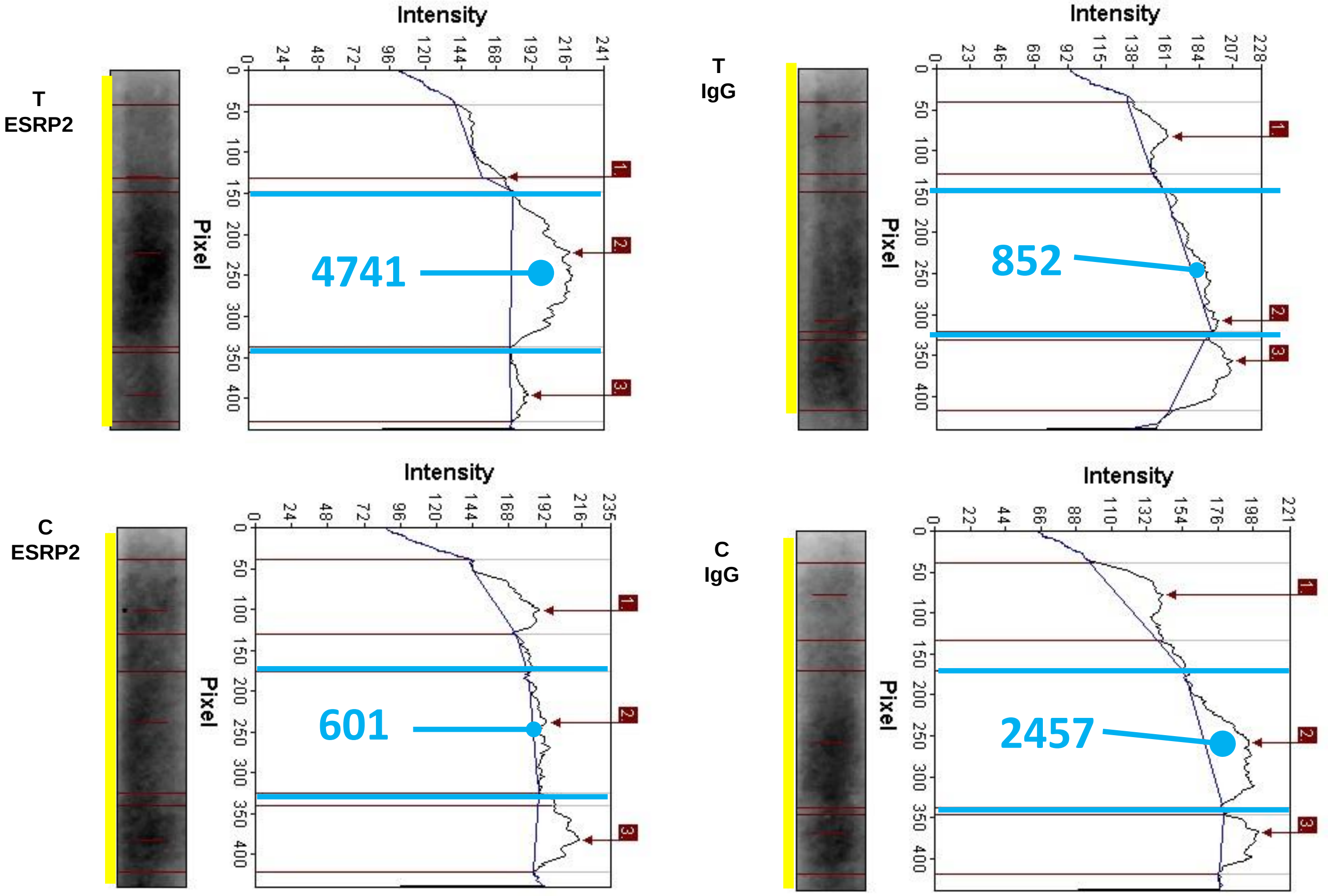

Raw volume was used to estimate ESRP2 bound on EMSA probes rs152731-T and rs152731-C. Signal intensity of the corresponding IgG lane was normalized to 100 %:

	anti-ESRP2-Ab	IgG
rs152731-T	556%	100%
rs152731-C	24%	100%

Source data EMSA / *Experiment 3 – uncurated data*

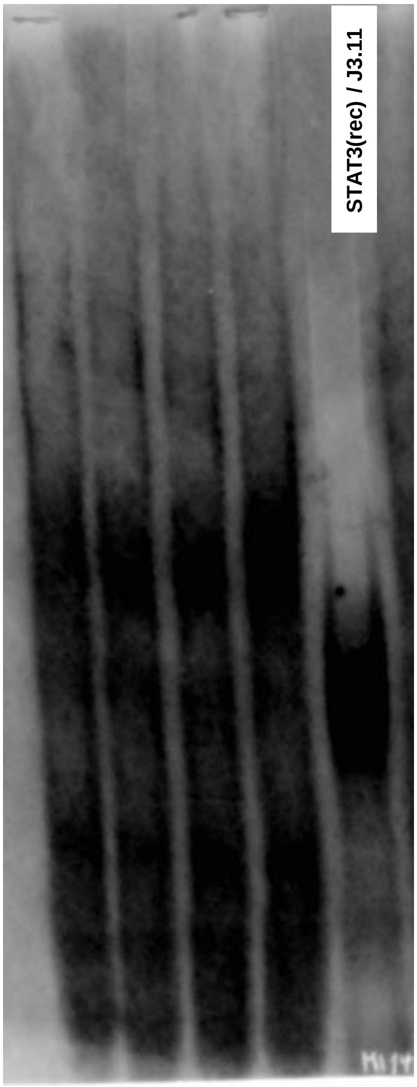

raw data

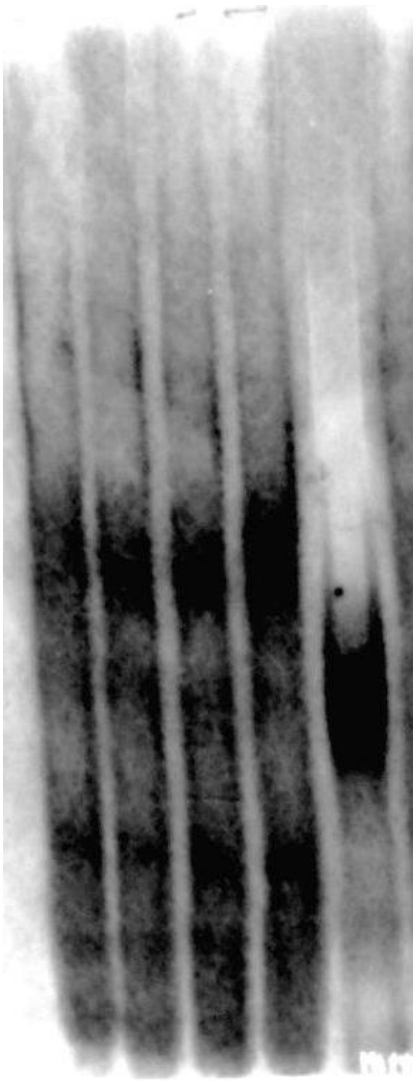

brightness and contrast adjusted for visualisation on screen

Legend:
EMSA Probe (rs152731 allele C or rs152731 allele T)
Ab (ESRP2 = anti-ESRP2-Ab; IgG = isotype control)
STAT3(rec) / J3.11: STAT3 recombinant protein & probe J3.11 (unrelated to ENaC/ESRP2)

Source data EMSA / *Experiment 3 – lane profiles*

T ESRP2 **T IgG** **C ESRP2** **C IgG**

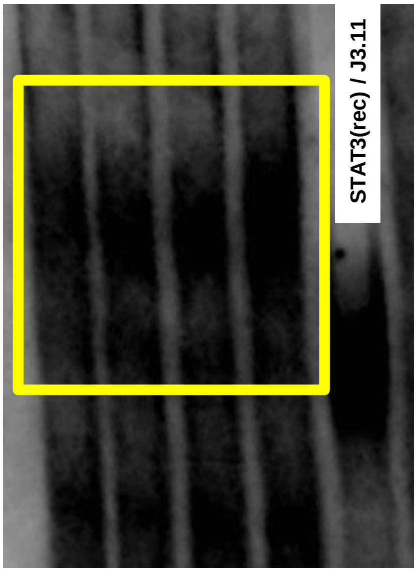

T ESRP2

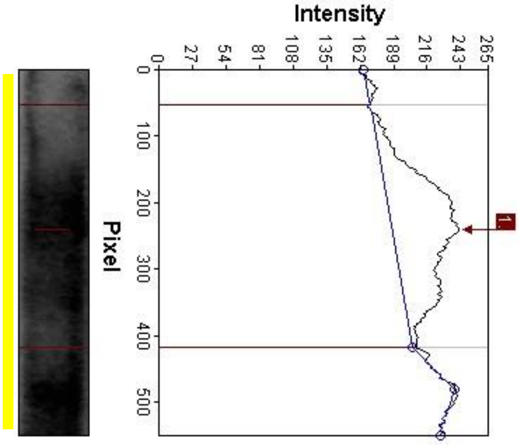

T IgG

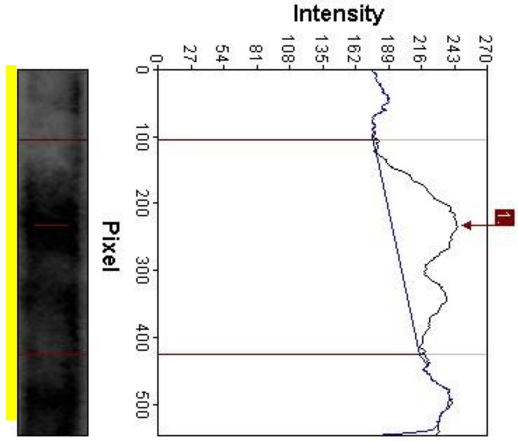

C ESRP2

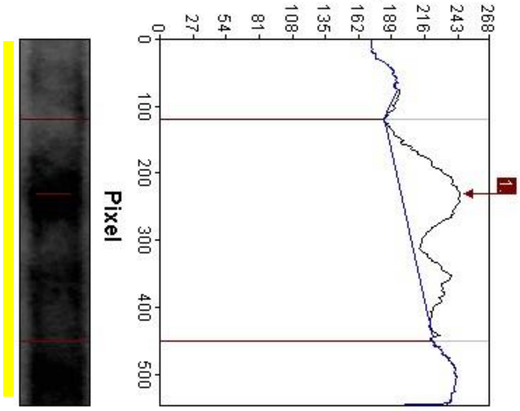

C IgG

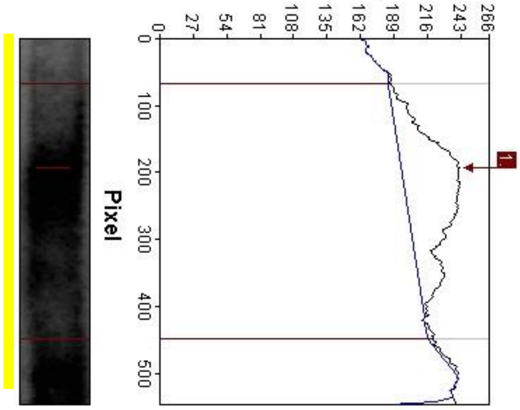

Source data EMSA / *Experiment 3 – densitometry*

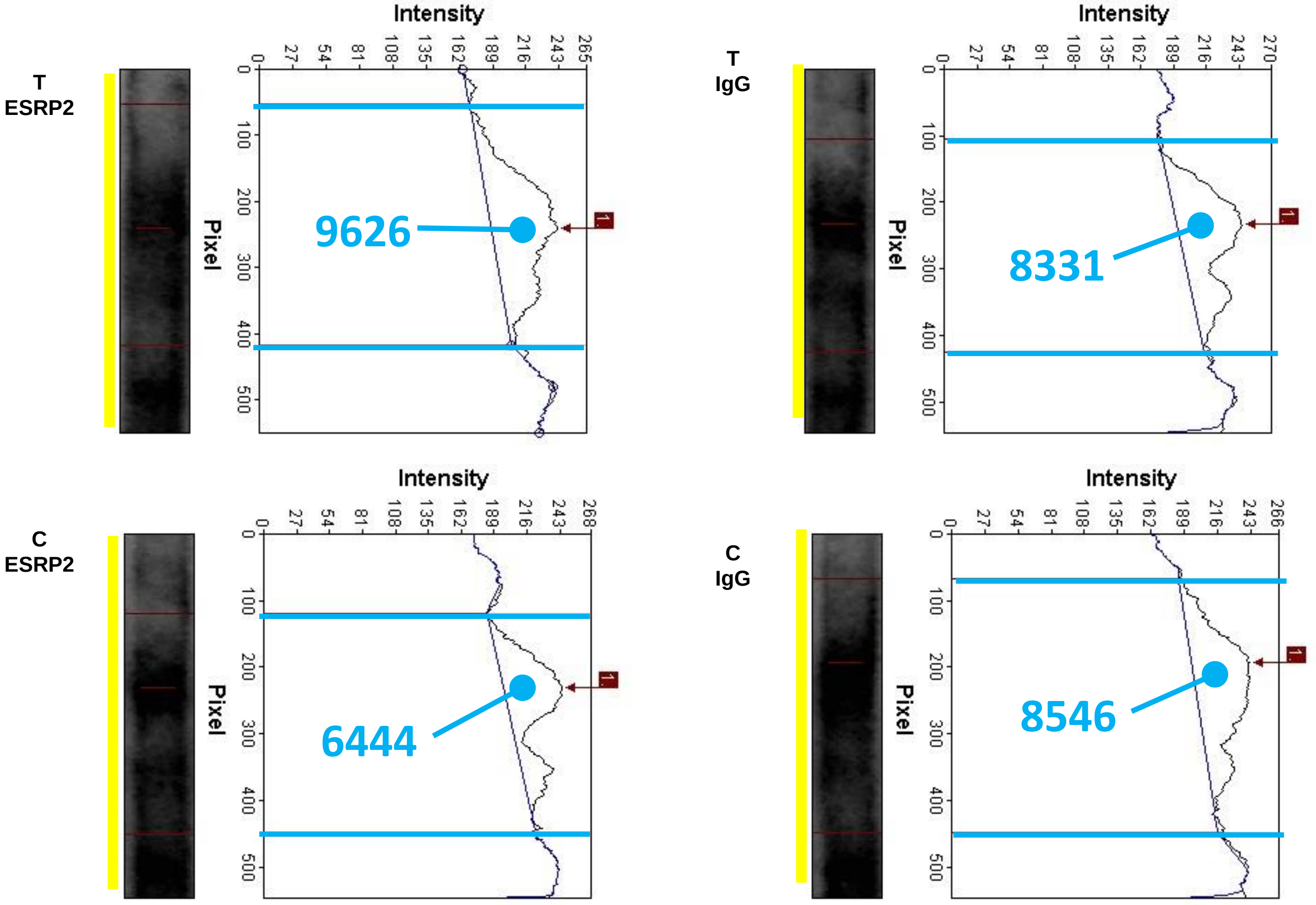

Raw volume was used to estimate ESRP2 bound on EMSA probes rs152731-T and rs152731-C. Signal intensity of the corresponding IgG lane was normalized to 100 %:

	anti-ESRP2-Ab	IgG
**rs152731-T**	115%	100%
**rs152731-C**	75%	100%

Source data EMSA / *Experiment 4 – uncurated data*

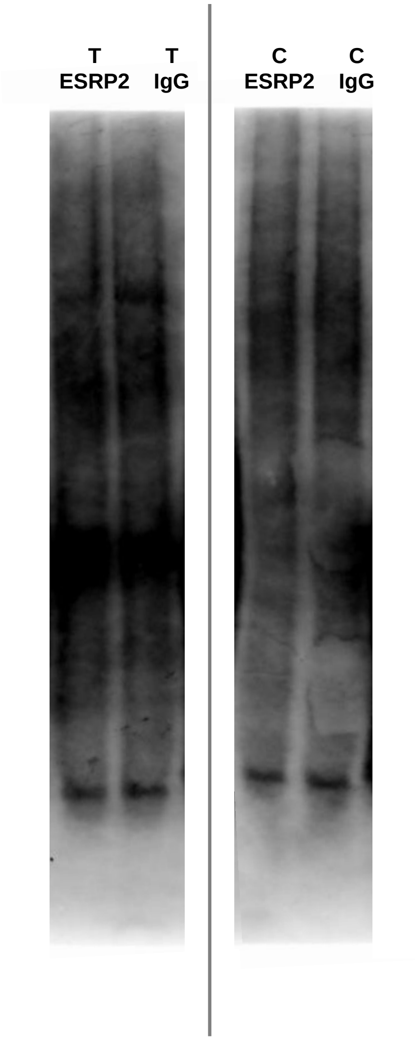

raw data

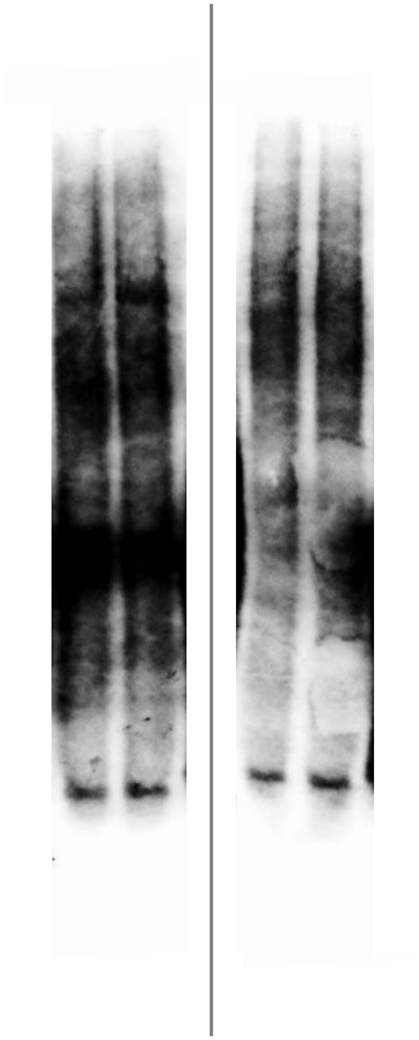

brightness and contrast adjusted for visualisation on screen

Legend:
EMSA Probe (rs152731 allele C or rs152731 allele T)
Ab (ESRP2 = anti-ESRP2-Ab; IgG = isotype control)

Source data EMSA / *Experiment 4 – lane profiles*

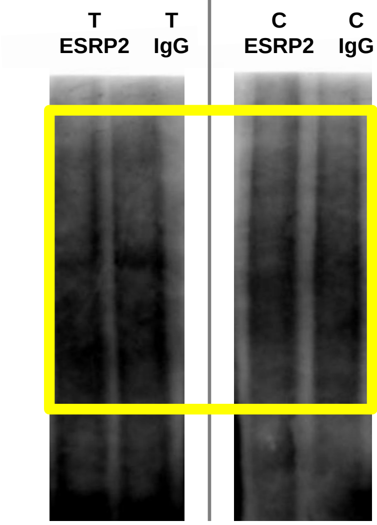

T ESRP2

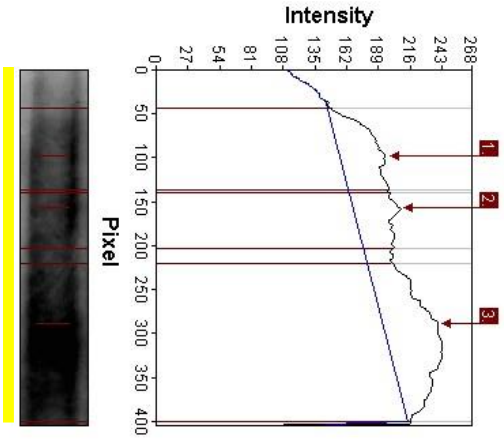

T IgG

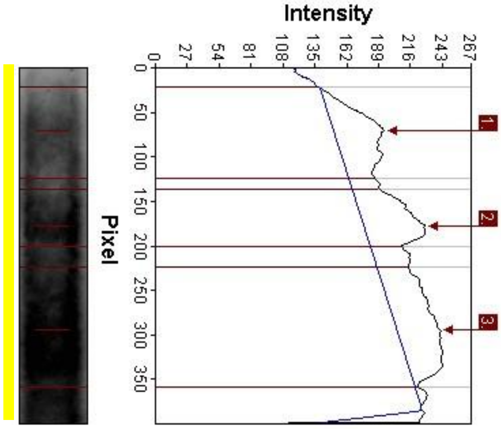

C ESRP2

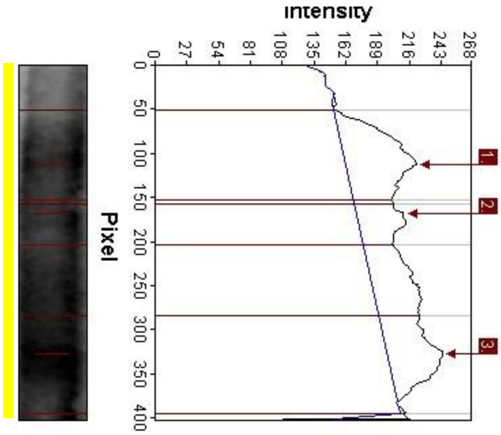

C IgG

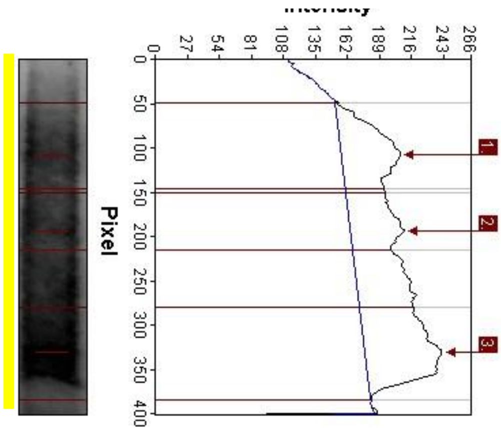

Source data EMSA / *Experiment 4 – densitometry*

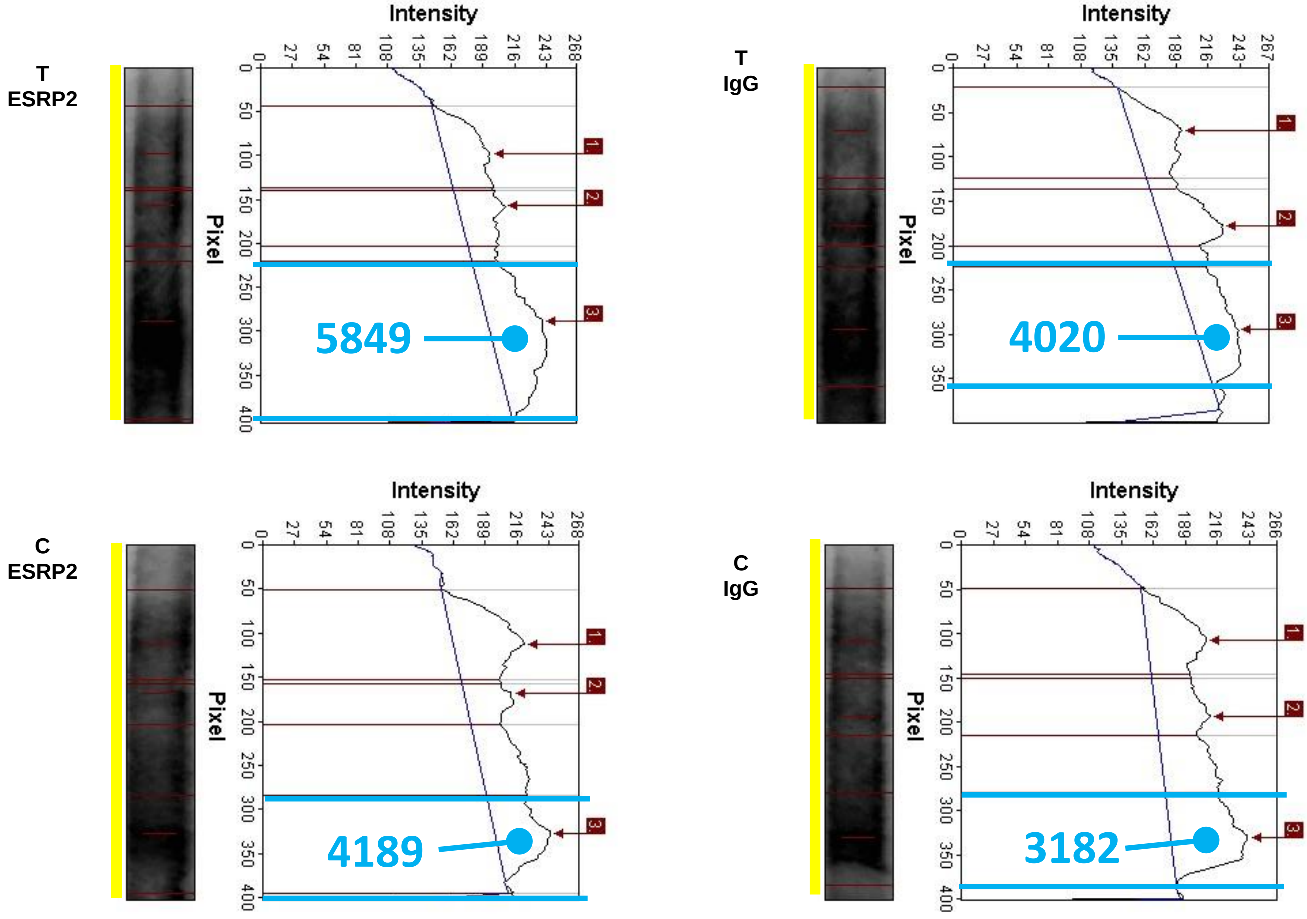

Raw volume was used to estimate ESRP2 bound on EMSA probes rs152731-T and rs152731-C. Signal intensity of the corresponding IgG lane was normalized to 100 %:

	anti-ESRP2-Ab	IgG
**rs152731-T**	145%	100%
**rs152731-C**	131%	100%

Source data EMSA / *Experiment 5 – uncurated data*

C ESRP2	T ESRP2	T IgG

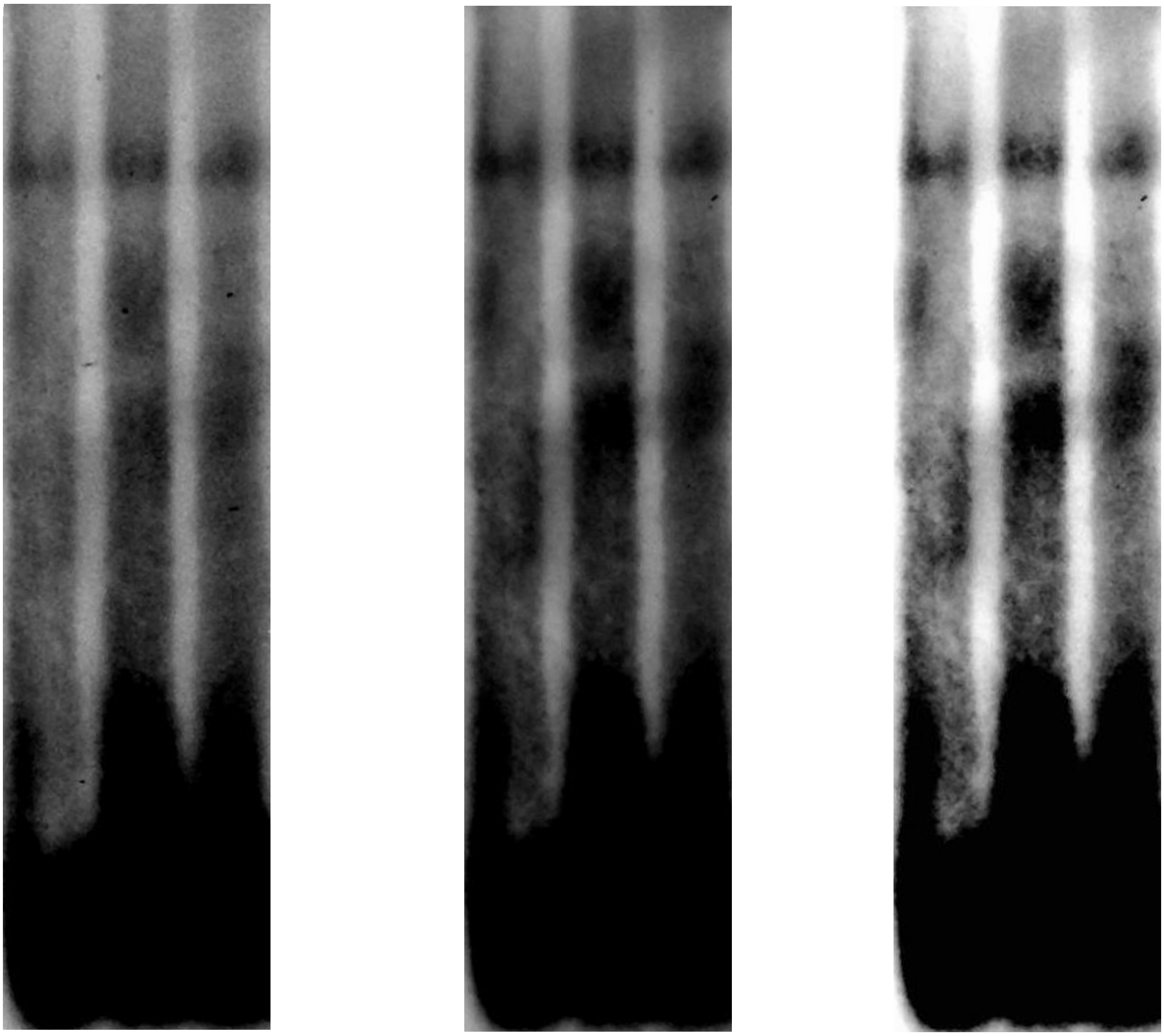

raw data 30 min exposure

raw data 60 min exposure

brightness and contrast adjusted for visualisation on screen

Legend:
EMSA Probe (rs152731 allele C or rs152731 allele T)
Ab (ESRP2 = anti-ESRP2-Ab; IgG = isotype control)

Source data EMSA / *Experiment 5 – lane profiles*

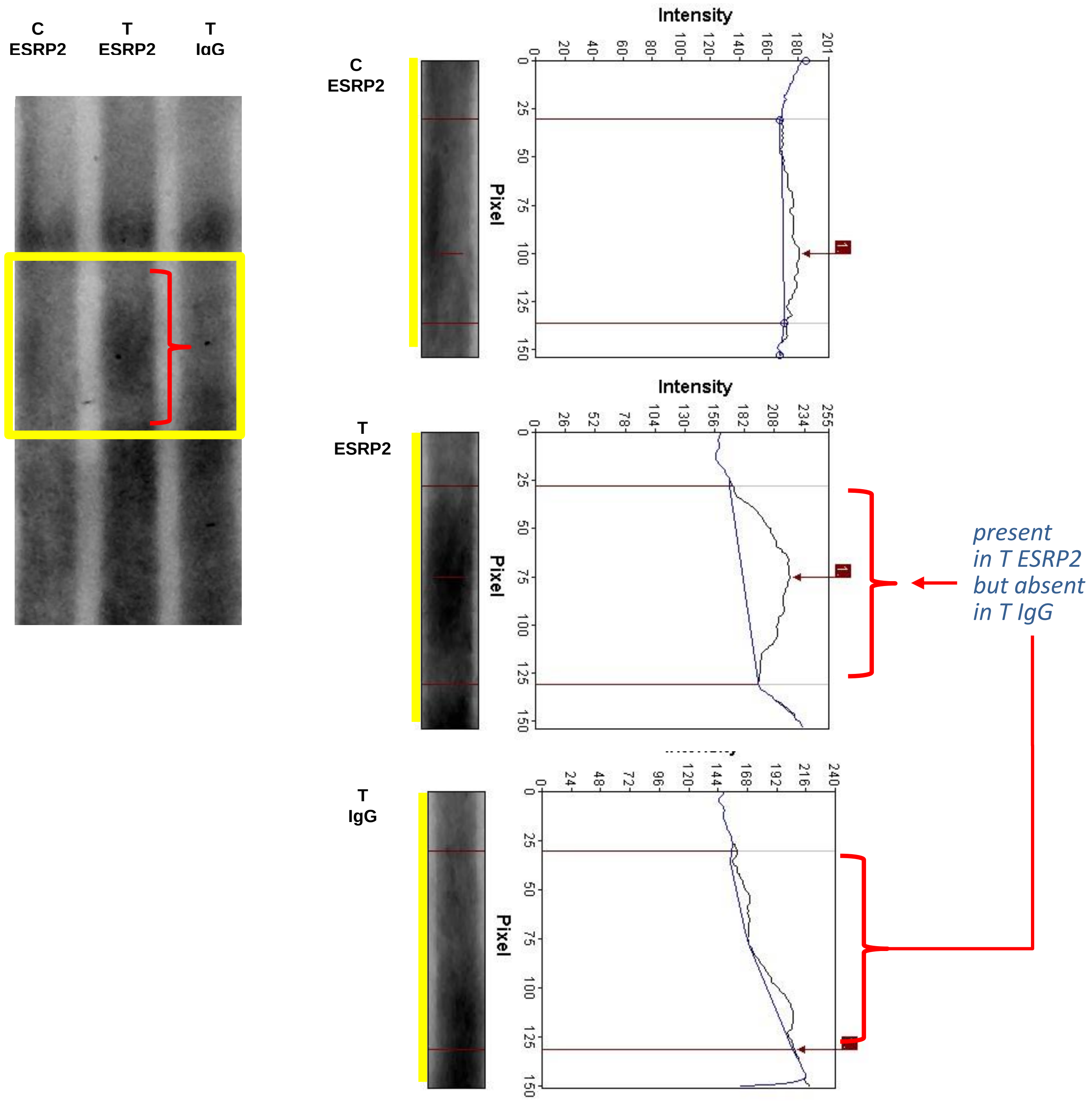

Source data EMSA / *Experiment 5 – densitometry*

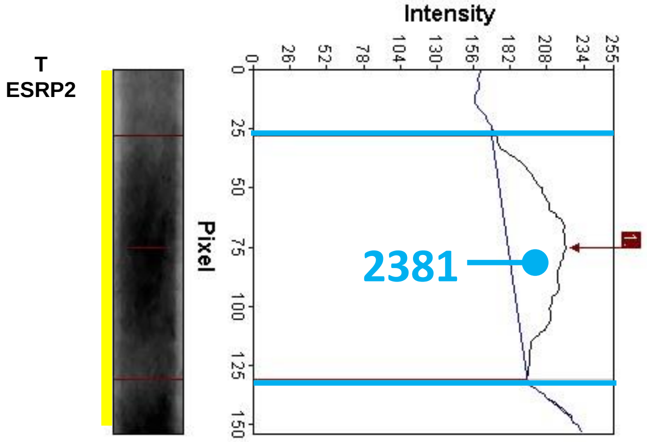

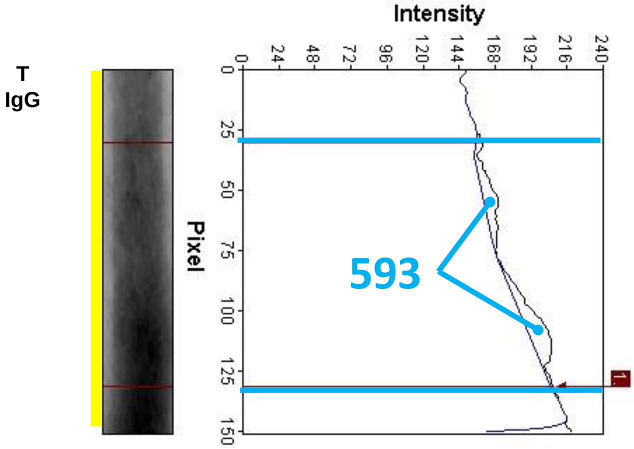

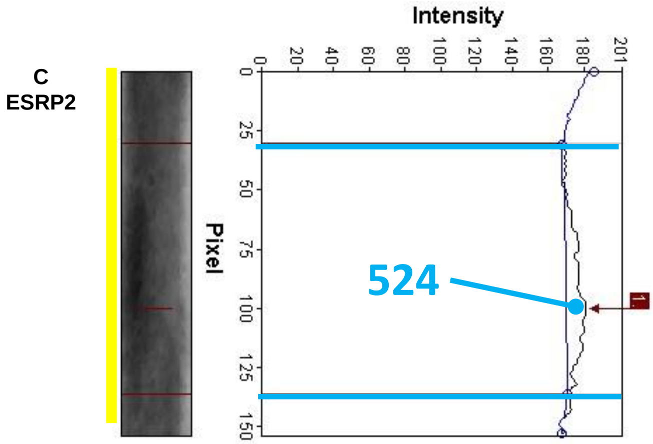

Raw volume was used to estimate ESRP2 bound on EMSA probes rs152731-T and rs152731-C. Signal intensity of the IgG lane was normalized to 100 %:

	anti-ESRP2-Ab	IgG
rs152731-T	401%	100%
rs152731-C	88%	100%

Source data EMSA / *Experiment 6 – uncurated data*

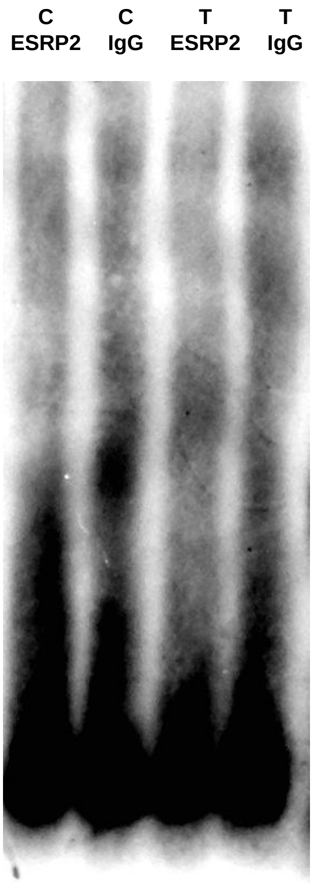

raw data
20 min exposure

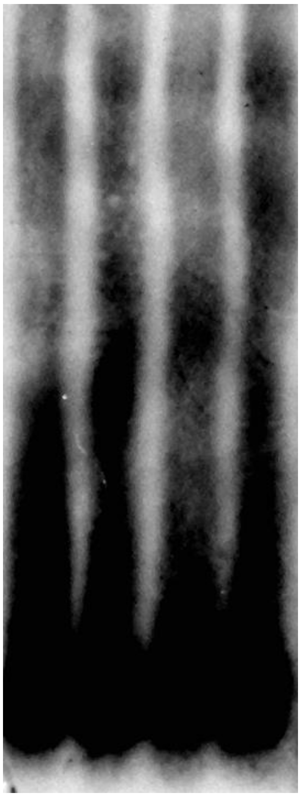

raw data
40 min exposure

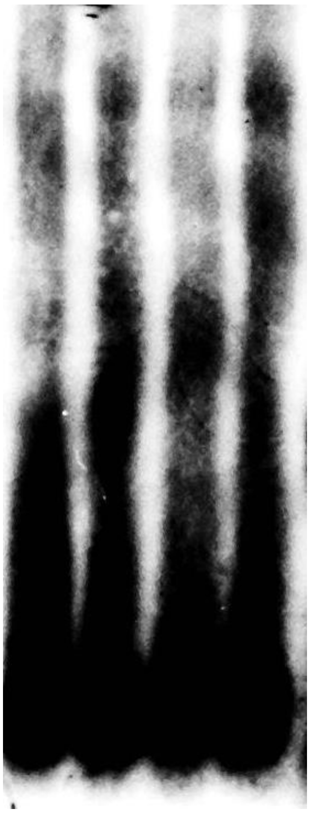

brightness and contrast adjusted for visualisation on screen

Legend:
EMSA Probe (rs152731 allele C or rs152731 allele T)
Ab (ESRP2 = anti-ESRP2-Ab; IgG = isotype control)

Source data EMSA / *Experiment 6 – lane profiles*

C ESRP2 | C IgG | T ESRP2 | T IgG

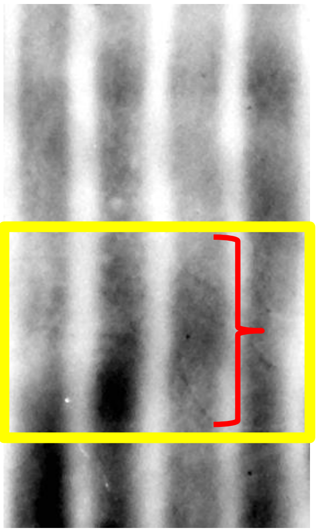

C ESRP2

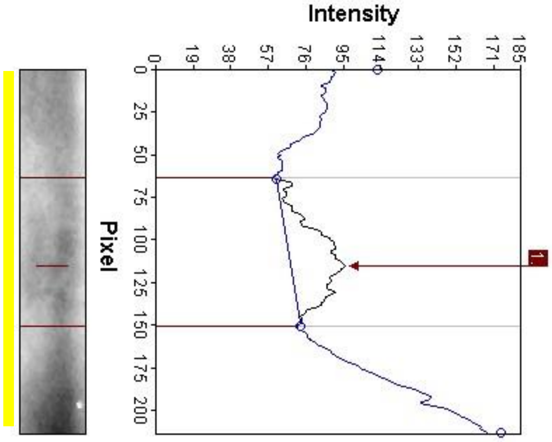

C IgG

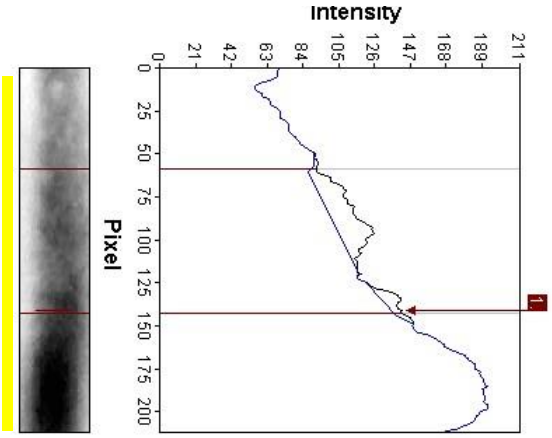

T ESRP2

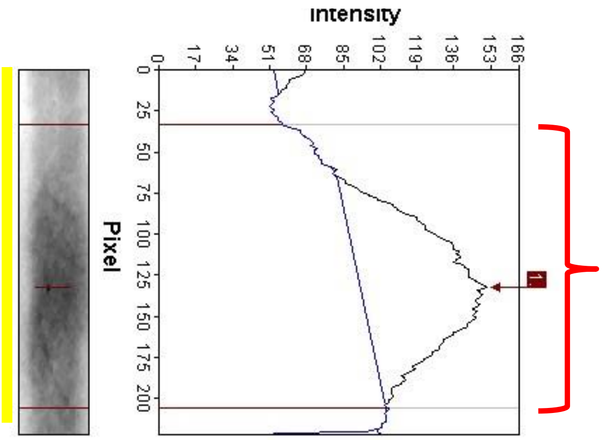

present in T ESRP2 but absent in T IgG

T IgG

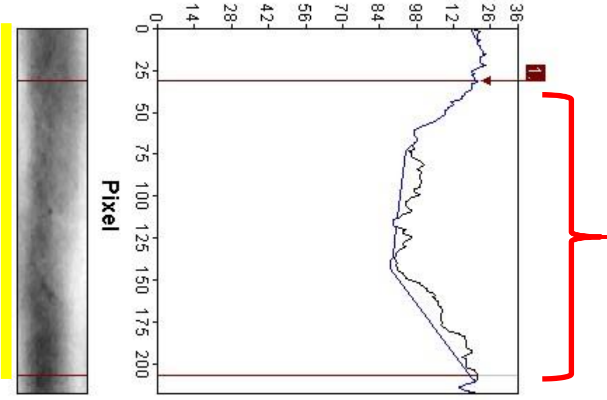

Source data EMSA / *Experiment 6 – densitometry*

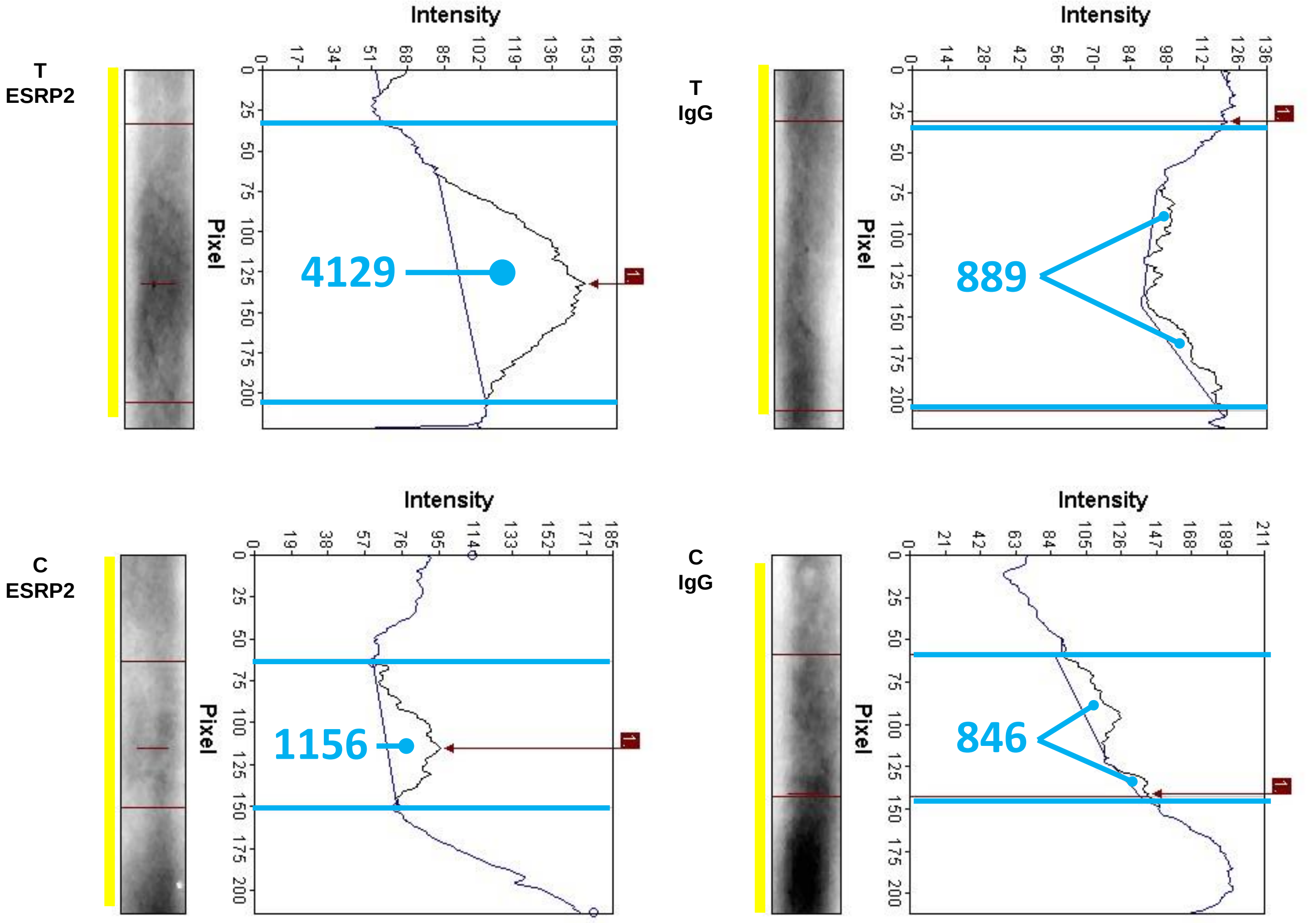

Raw volume was used to estimate ESRP2 bound on EMSA probes rs152731-T and rs152731-C. Signal intensity of the corresponding IgG lane was normalized to 100 %:

	anti-ESRP2-Ab	IgG
**rs152731-T**	464%	100%
**rs152731-C**	137%	100%

Source data co-IP / Figure 6C,D

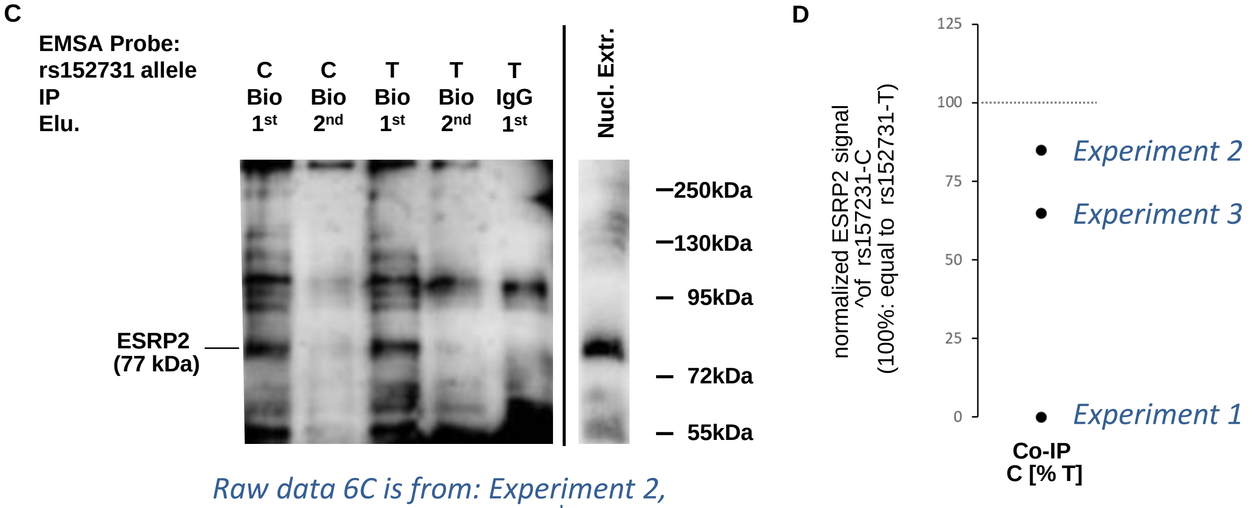

Raw data 6C is from: Experiment 2,
exposure 10 min (co-IP) | 30 sec (nuclear extract)

Nuclear extracts used in experiment 1, 2 and 3 were from T84.

Dokumentation of primary data (GLP rules, Hannover medical school):

Experiment 1: Lab Book 7452,	*pages 48-50 (Immunprecipitation)*
	pages 54-57 (ESRP2 detection)
Experiment 2: Lab Book 7452,	*pages 62-64 (Immunprecipitation)*
	pages 68-72 (ESRP2 detection)
Experiment 3: Lab Book 7452,	*pages 84-86 (Immunprecipitation)*
	pages 89-92 (ESRP2 detection)

Source data co-IP / *Experiment 1 – uncurated data*

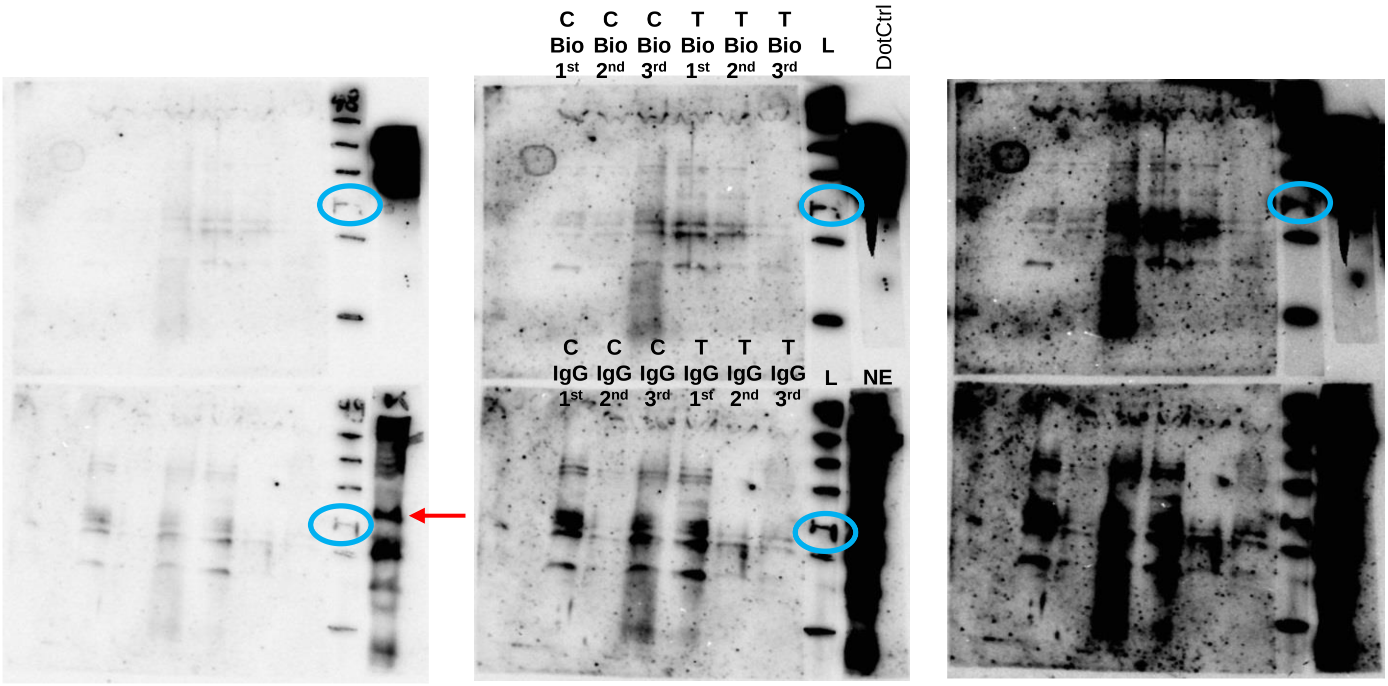

exposure 3 min *exposure 30 min* *exposure 1 h*

Legend:
EMSA Probe (rs152731 allele C or rs152731 allele T)
Ab for IP (Bio = anti-biotin-Ab; IgG = isotype control)
Eluate (1st, 2nd, 3rd)

L = Ladder ; band sizes are: 36 kDa, 55 kDa, 72 kDa, 95 kDa, 130 kDa, 250 kDa;
72 kDa is encircled in blue

NE = T84 nuclear extract, expected size of ESRP2 = 77 kDa (marked by red arrow)

DotCtrl = Dot Control; set of 10 fold dilution of biotinylated probe developed in parallel

Source data co-IP / *Experiment 1 – ESRP2 in co-IP (red box)*

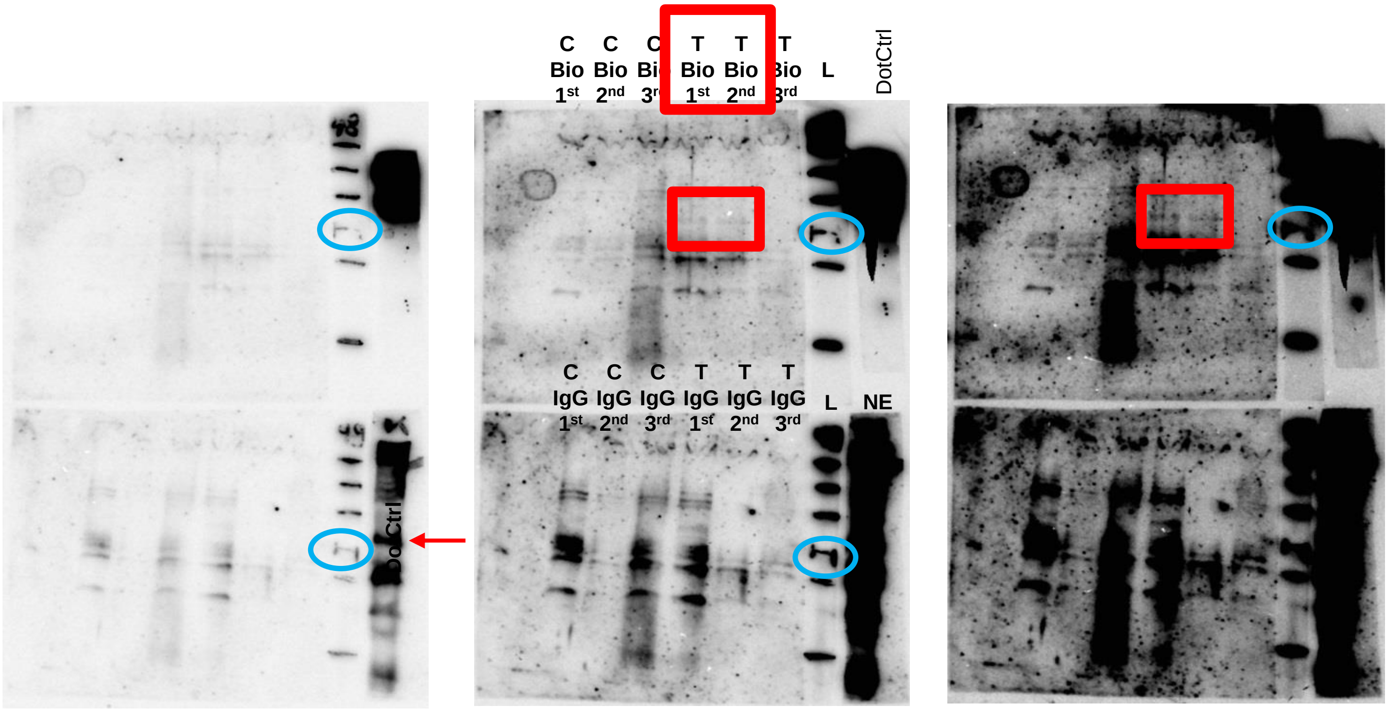

exposure 3 min *exposure 30 min* *exposure 1 h*

As no immunoreactive signal from samples with EMSA Probe rs152731 allele C was observed, experiment 1 was not evaluated by densitometry.

Source data co-IP / *Experiment 2 – uncurated data*

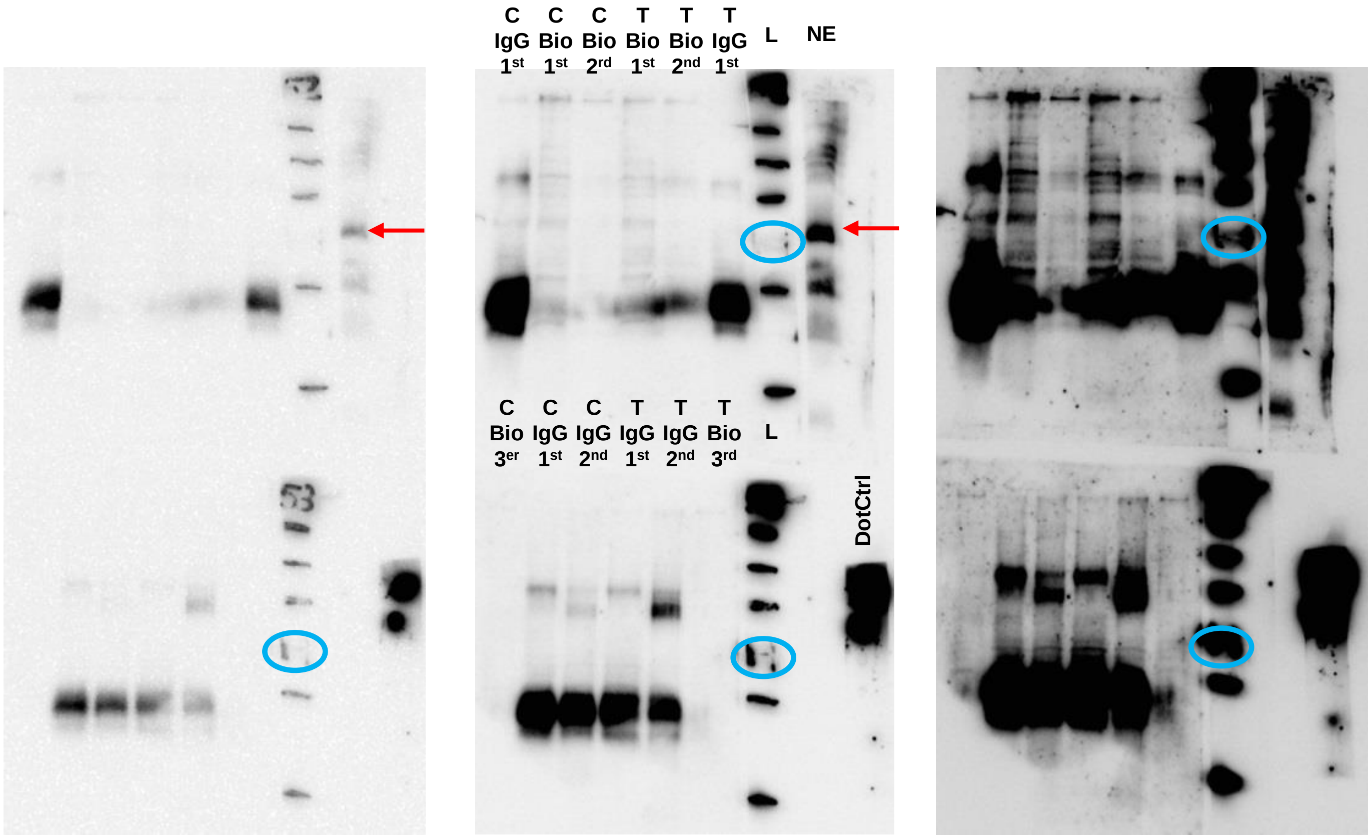

exposure 10 sec *exposure 1 min* *exposure 10 min*

Legend:
EMSA Probe (rs152731 allele C or rs152731 allele T)
Ab for IP (Bio = anti-biotin-Ab; IgG = isotype control)
Eluate (1st, 2nd, 3rd)
L = Ladder ; band sizes are: 36 kDa, 55 kDa, 72 kDa, 95 kDa, 130 kDa, 250 kDa;
72 kDa is encircled in blue
NE = T84 nuclear extract, expected size of ESRP2 = 77 kDa (marked by red arrow)
DotCtrl = Dot Control; set of 10 fold dilution of biotinylated probe developed in parallel

Source data co-IP / *Experiment 2 – ESRP2 in co-IP (red box)*

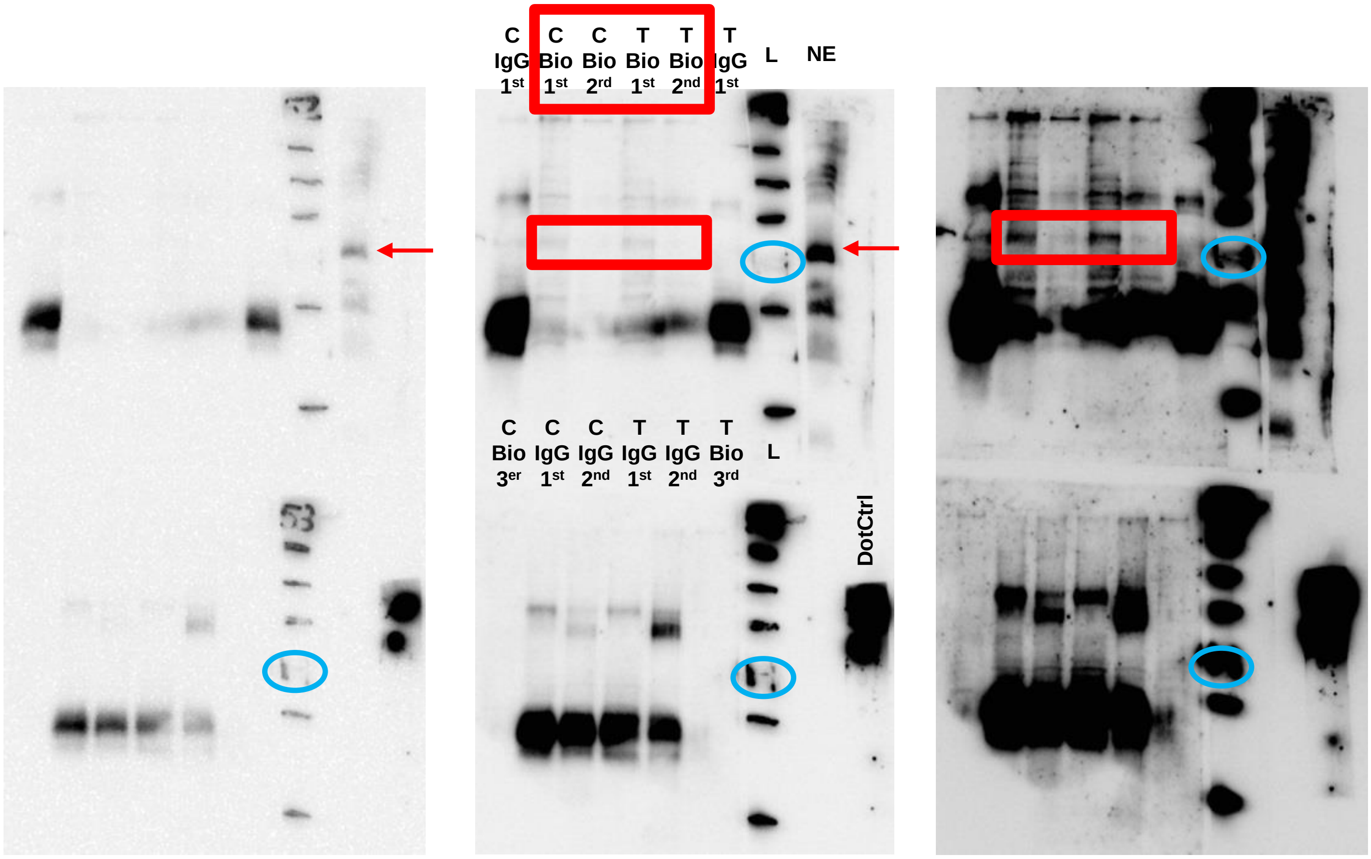

exposure 10 sec *exposure 1 min* *exposure 10 min*

Figure 6C:

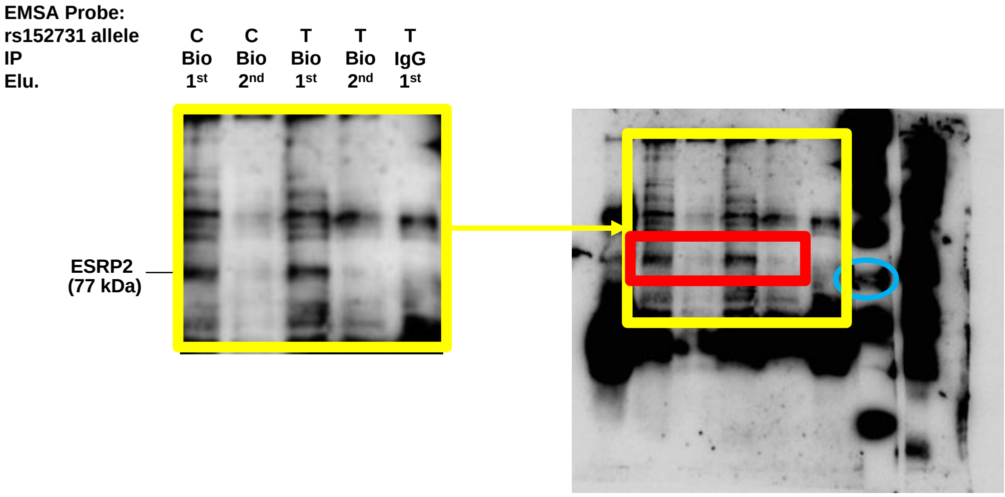

Source data co-IP / *Experiment 2 - densitometry*

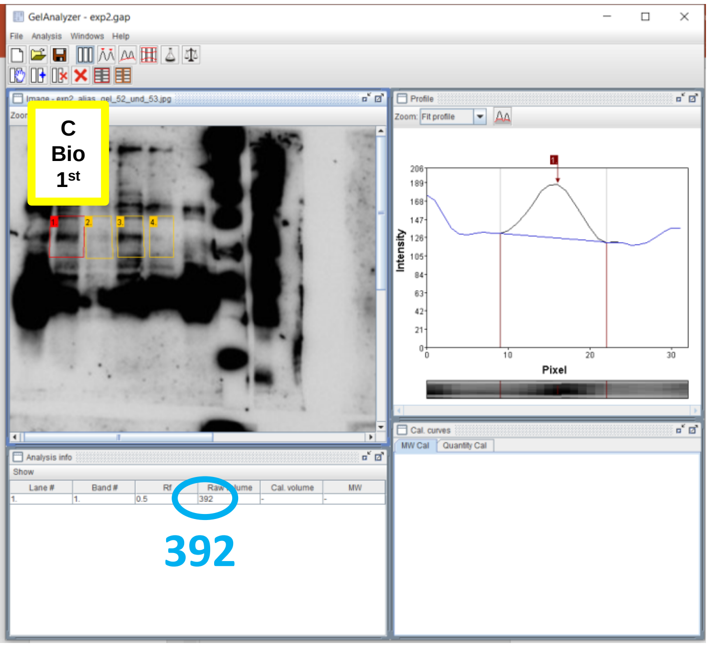

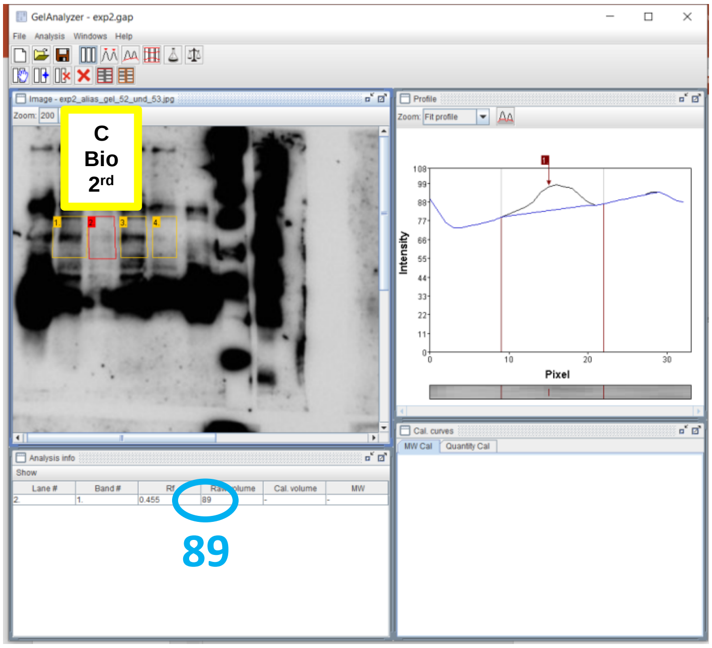

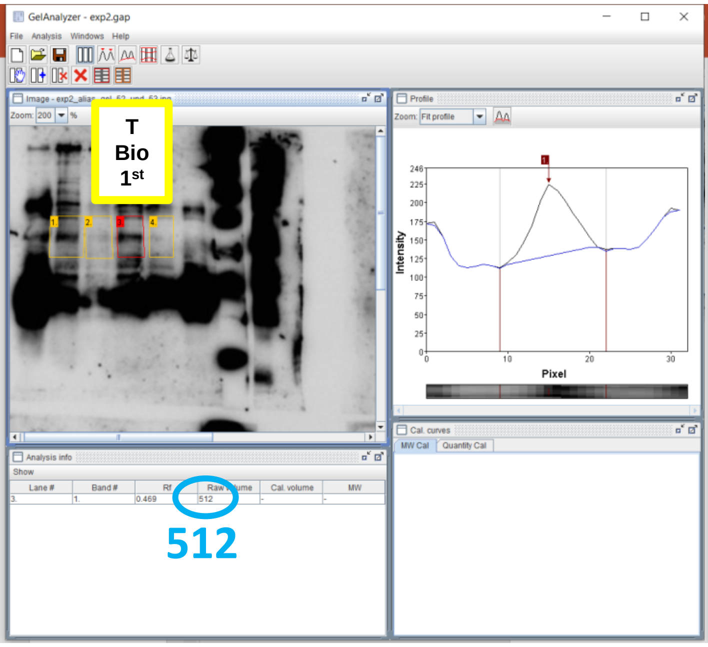

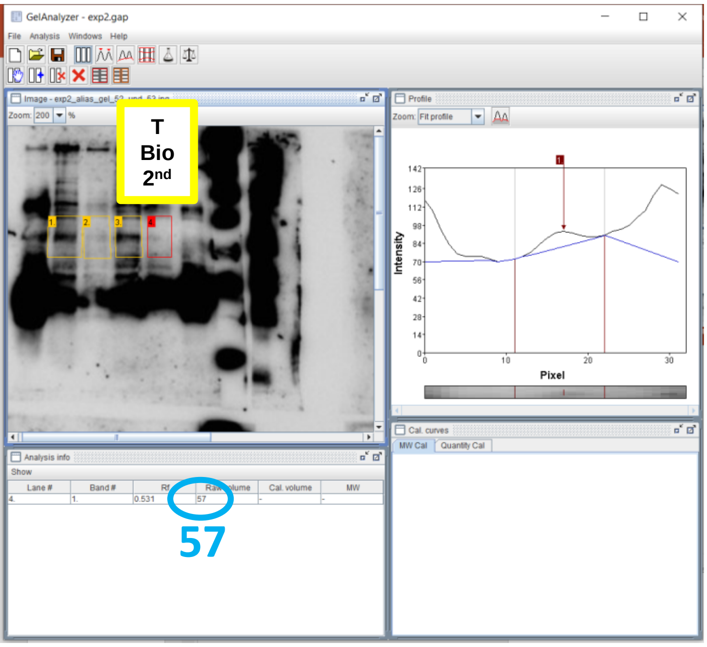

Raw volume, summarized from 1st and 2nd eluate, was used to estimate ESRP2 bound on EMSA probes.
Signal intensity of ESRP2 immunoreactive signal on EMSA Probe rs152731 allele C, expressed as % of signal intensity of ESRP2 immunoreactive signal on EMSA Probe rs152731 allele T: 85%

Software used for analysis:
GelAnalyzer 2010a (www.gelanalyzer.com) by Istvan Lazar Jr., PhD and Istvan Lazar Sr., PhD, CSc

Source data co-IP / *Experiment 3 - uncurated data*

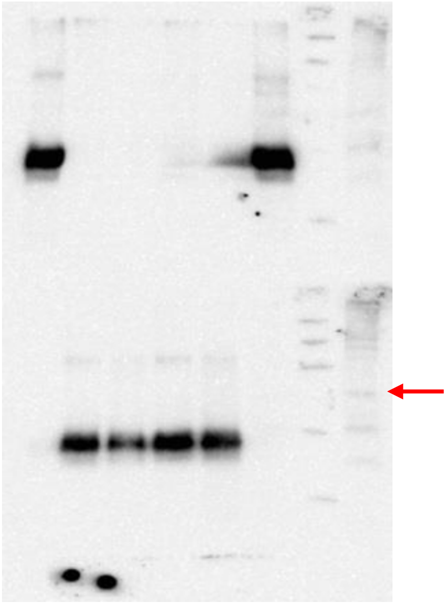

exposure 10 sec

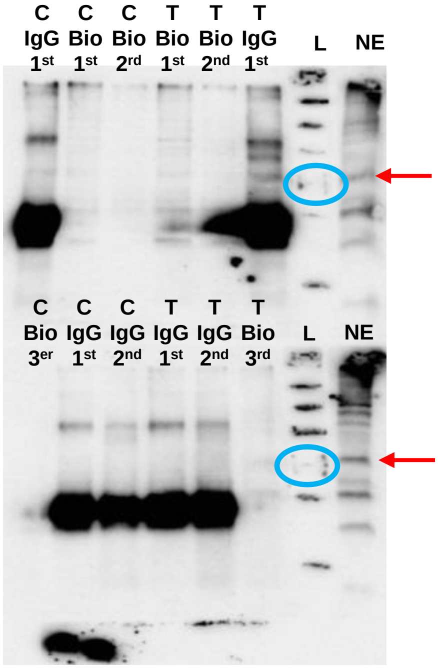

exposure 1 min

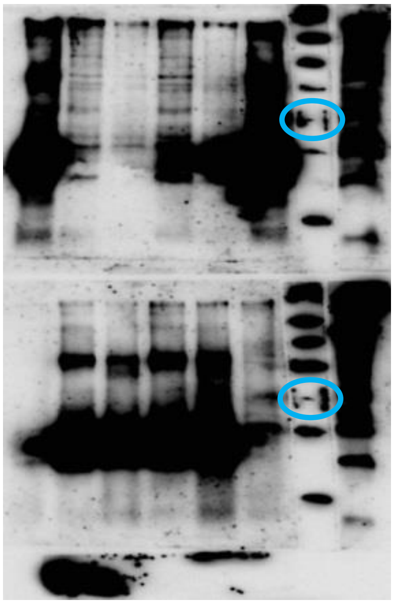

exposure 10 min

Legend:
EMSA Probe (rs152731 allele C or rs152731 allele T)
Ab for IP (Bio = anti-biotin-Ab; IgG = isotype control)
Eluate (1st, 2nd, 3rd)
L = Ladder ; band sizes are: 36 kDa, 55 kDa, 72 kDa, 95 kDa, 130 kDa, 250 kDa;
72 kDa is encircled in blue
NE = T84 nuclear extract, expected size of ESRP2 = 77 kDa (marked by red arrow)

Source data co-IP / *Experiment 3 – ESRP2 in co-IP (red box)*

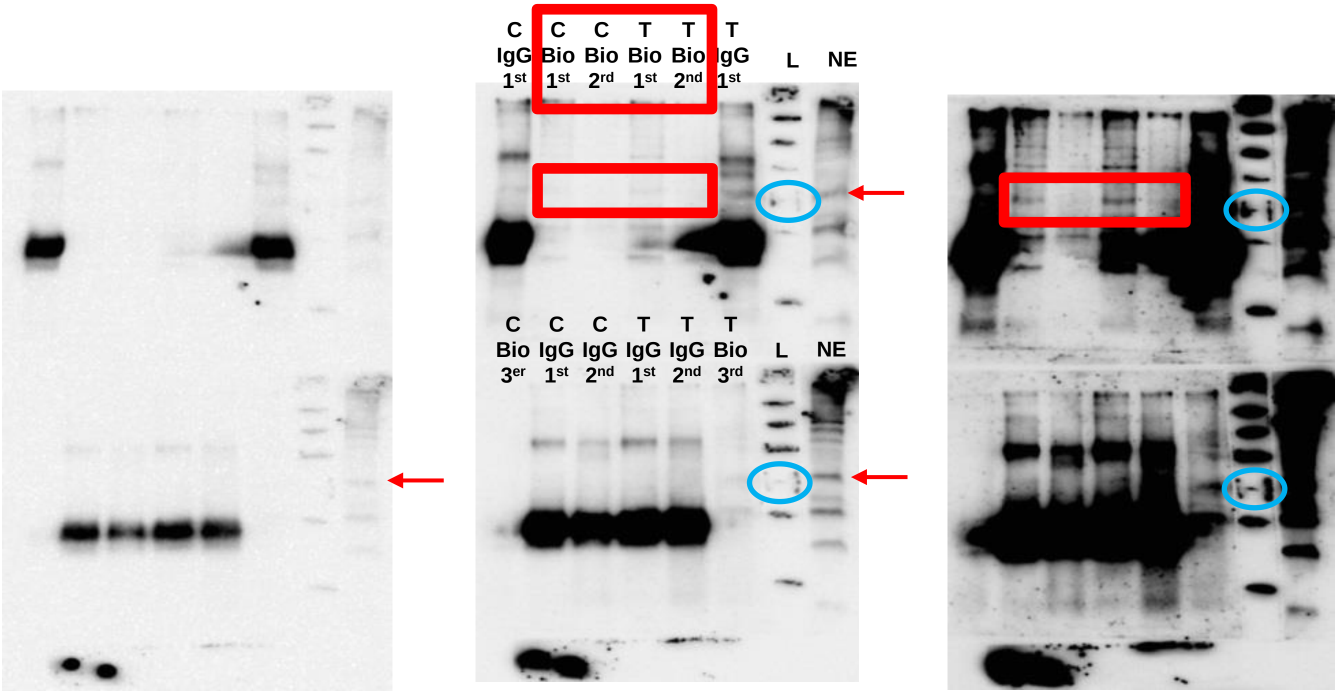

exposure 10 sec

exposure 1 min

exposure 10 min

Source data co-IP / *Experiment 3 - densitometry*

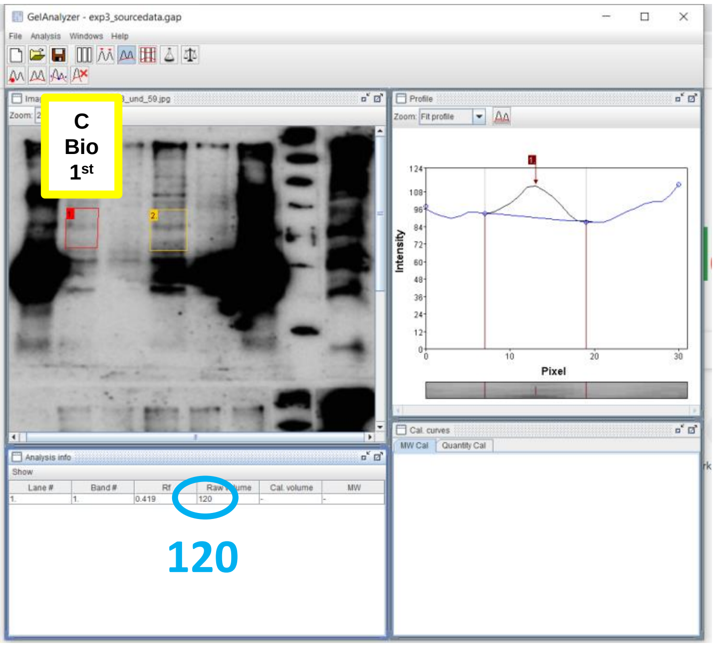

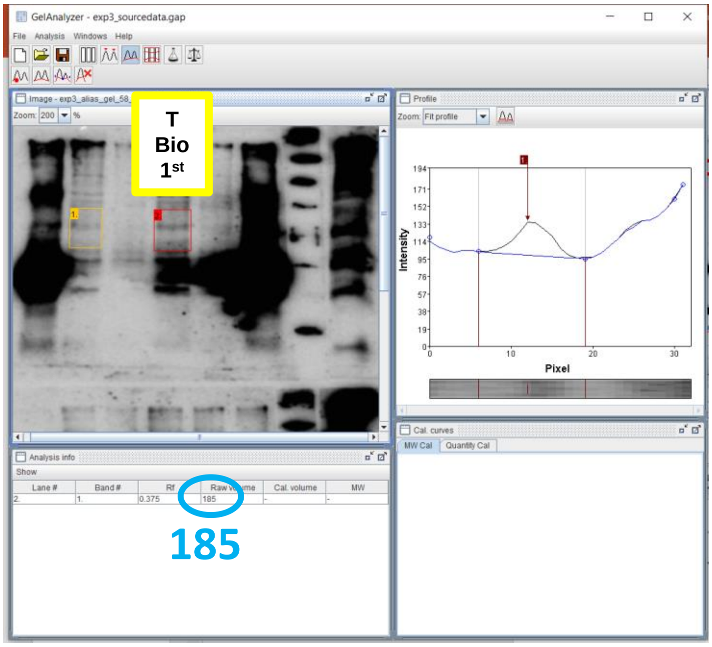

Raw volume was used to estimate ESRP2 bound on EMSA probes.
Signal intensity of ESRP2 immunoreactive signal on EMSA Probe rs152731 allele C, expressed as % of signal intensity of ESRP2 immunoreactive signal on EMSA Probe rs152731 allele T: 65%

Software used for analysis:
GelAnalyzer 2010a (www.gelanalyzer.com) by Istvan Lazar Jr., PhD and Istvan Lazar Sr., PhD, CSc
